# A Spatially Explicit Societal Life Cycle Cost Framework for Sustainable Biographite Production in the United States

**DOI:** 10.1021/acs.est.6c05424

**Published:** 2026-09-26

**Authors:** Hannah Szu-Han Wang, Jose A. Gonzalez-Aguirre, Sunkyu Park, Yuan Yao

**Affiliations:** † Center for Industrial Ecology, Yale School of the Environment, 5755 Yale University, 380 Edwards Street, New Haven, Connecticut 06511, United States; ‡ Department of Forest Biomaterials, College of Natural Resources, 6798 North Carolina State University, 2820 Faucette Dr, Raleigh, North Carolina 27607, United States; § Chemical and Environmental Engineering, Yale School of Engineering and Applied Science, 5755 Yale University, New Haven, Connecticut 06511, United States

**Keywords:** graphite, lignocellulosic residues, life cycle assessment, techno-economic analysis, societal life cycle cost, uncertainty analysis, regionalized TEA-LCA

## Abstract

Graphite demand is projected to double by 2040, yet the United States relies heavily on imports, motivating domestic biographite production from abundant lignocellulosic residues. However, large-scale biographite production is hindered by uncertain environmental and economic implications. We address the challenge by developing a spatially explicit societal life cycle cost (sLCC) framework that integrates regionalized techno-economic analysis (TEA) and life cycle assessment (LCA). We apply the method to assess U.S. biographite production across various process designs and location choices. TEA and LCA identify process pathway, electricity, catalysts, feedstocks, and byproduct upgrades as key factors influencing biographite’s sustainability performance. Biographite production in most states is cost-competitive ($4.1–5.4/kg), but its environmental advantages over market synthetic graphite vary by location and impact category. sLCC reveals that biographite production in the Eastern U.S. is ∼18% more preferable than market synthetic graphite, while benefits are limited in Pacific and Upper regions.

## Graphical abstract

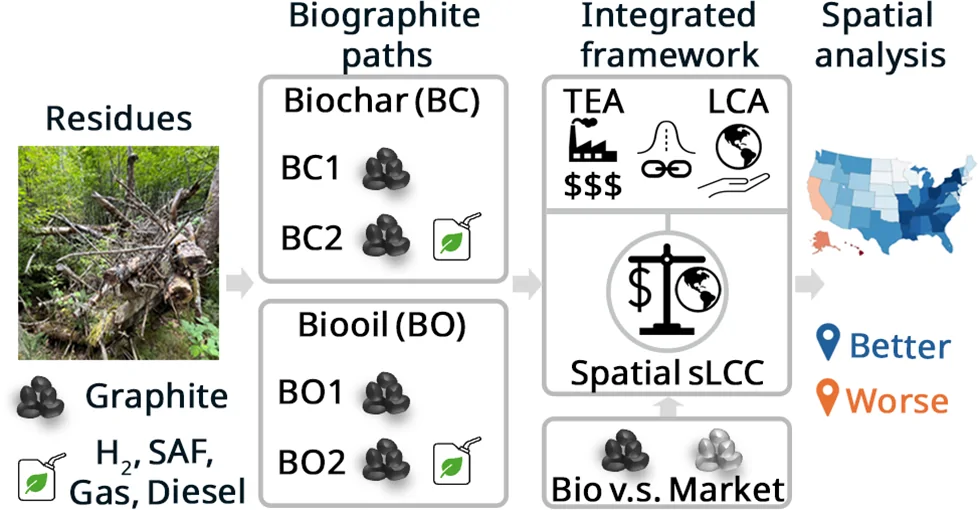

## 1 Introduction

Graphite is a critical material widely used in industries from electric vehicles (EV) and energy storage to steelmaking,^1–3^ owing to its superior thermal-chemical stability and electrical conductivity.^1,2^ Commercial battery-grade graphite is primarily supplied as natural or synthetic graphite. Natural graphite is obtained through mining and subsequent processing, whereas synthetic graphite is produced from carbonaceous feedstocks, commonly petroleum coke, through energy-intensive thermal processing that includes high-temperature graphitization. Global graphite demand is expected to double by 2040 (reaching 11 million metric tons),^4^ primarily driven by growth in China, Europe, and the U.S. However, the U.S. graphite supply chain remains vulnerable due to heavy reliance on imports.^5^ To build a resilient supply chain, exploring domestic carbonaceous feedstocks for graphite production is essential. Lignocellulosic biomass is a promising candidate due to its renewability,^6^ high carbon content, and abundance in the U.S.^7^

Biomass can be converted to graphite via two paths, often referred to as biographite.^2,8,9^ One is biochar to graphite,^8–10^ where biomass undergoes slow, oxygen-limited heating (i.e., slow pyrolysis with residence times ranging from minutes to days^11^) to produce biochar that is then graphitized. The other path is biooil to graphite,^12,13^ where biomass is rapidly heated (i.e., fast pyrolysis with ∼1 s^11^) to produce bio-oil, which is then condensed and coked to a solid carbon (biocoke) before graphitization. Graphitization in both pathways involves catalytic (1300–1700 °C)^2,8,10,12–16^ or noncatalytic high-temperature (2400–3000 °C)^17,18^ processes. Previous studies have reported promising biographite quality,^2,9^ including high carbon yield^19^ and electrochemical properties.^8,12,15^ Nevertheless, these processes are energy- and material-intensive and produce greenhouse gas (GHG) emissions.^2^ A comprehensive sustainability evaluation is necessary to understand the environmental and economic implications of deployment at scale and for process improvements.

Techno-economic analysis (TEA) and life cycle assessment (LCA) are commonly used sustainability assessment tools. Existing TEAs^14,20,21^ and LCAs for biographite^15,19,22^ are limited to a specific site, conversion pathways, or process design, making results difficult to generalize across locations and systems. Prior TEAs investigated biomass-to-graphite paths using different feedstocks and process designs,^14,20,21^ reporting production costs as low as USD ($) 1.67 kg^–1^ of graphite and minimum selling prices (MSPs) ranging from $ 3.3–11 kg^–1^ of graphite, spanning economically competitive to uneconomic cases. Previous LCAs showed that the environmental impacts of biographite are sensitive to the grid electricity sources. For example, Kulkarni et al.^22^ found that biochar precursors from Indonesia, where the electricity grid is fossil-based, can perform worse than market synthetic graphite, while using cleaner energy may reverse the result. These separate TEA and LCA studies leave knowledge gaps in potential environmental-economic trade-offs and co-benefits across different process designs and location choices. It remains unclear whether locations with low production costs also show low environmental impact, which is critical for siting decisions and supply chain development.^23,24^

To address these gaps, we develop a spatially explicit and generalizable societal life cycle cost (sLCC) framework for biographite production. This framework integrates LCA and TEA via consistent process inventories and incorporates region-specific prices and emission factors to capture spatial variability. It combines monetized LCA-derived environmental impacts and TEA-derived resource costs within a societal discounting framework to estimate the overall societal cost of biographite production (see Methods and Note S1 for details). Here, sLCC is distinct from social life cycle assessment, which evaluates social and socioeconomic impacts associated with stakeholder groups such as workers and local communities.^25^ We applied the framework to four biomass-to-graphite pathways across the U.S. to understand how process design and regional factors altogether shape the societal feasibility of biographite, and where biographite may be preferable to market synthetic/natural graphite.

## 2 Materials and Methods

We developed a spatial sLCC framework (Figure 1) to quantify the environmental and economic performance of biographite produced from lignocellulosic residues via four pathways, including two biochar (BC: BC1, BC2) and two biocoke paths (BO: BO1, BO2). This study focuses on lignocellulosic residues due to their substantial resource potential in the United States.^26,27^ The 2023 Billion-Ton report estimates a near-term forest biomass (e.g., forest wastes, logging and mill residues, small-diameter trees) potential of 27.5 million dry metric tonnes yr^–1^ (Table S1).^28^ The representative feedstock composition used for process simulation is defined separately based on literature-reported lignocellulosic feedstock properties (Table S2).^14,29–31^

**Figure 1:**
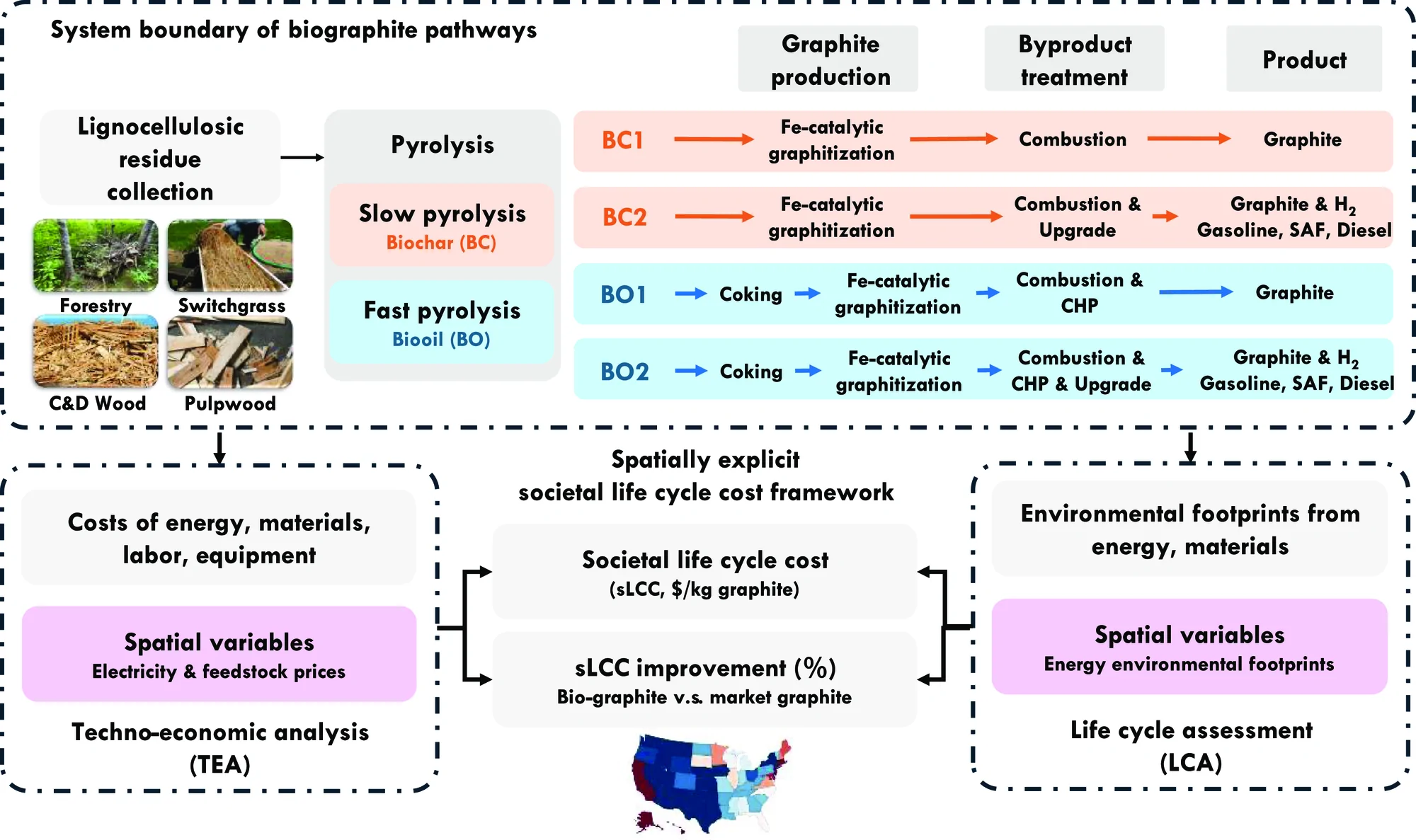
Schematic of the Societal Life Cycle Cost Framework and the system boundary of four biographite production pathways. C&D Wood: construction and demolition wood waste. Gas: gasoline. SAF: sustainable aviation fuel. Byproduct: pyrolysis vapor, condensates, noncondensable gases (NCG), and biochar (applicable to BO1 and BO2). Byproduct treatment: combustion represents byproduct combustion to generate process heat; upgrade represents upgrading byproducts through hydrotreating and NCG conversion to generate hydrocarbons (gasoline, SAF, diesel) and H_2_; CHP represents combined heat and power generation to generate electricity and process heat. Auxiliary processes, including cooling and wastewater treatment, are shared across pathways and are not shown for simplicity. Orange represents biochar (BC)-specific pathway; blue represents biocoke (BO)-specific pathway. Detailed process flow diagrams for each pathway are provided in Figures S1–S9.

The system boundary is consistent across process simulations, LCA, and TEA, including activities shown in Figure 1 and described in the following section on the process simulation. Process simulations first generate foreground material and energy inventories, which are subsequently used in LCA and TEA. Simulated streams, prices, and their emission factors are then varied in sensitivity and uncertainty analyses. Location-dependent variables are further used to regionalize LCA-TEA across the U.S. Finally, TEA and monetized LCA results are integrated to derive sLCC, and the sLCC improvement (%), defined as the difference between biographite and market graphite, is analyzed to inform region-specific decision-making. Two market graphite benchmarks are considered: market synthetic graphite and market natural graphite. Their production routes, inventory sources, and environmental impacts are summarized in Table S3.

### 2.1 Process Simulation

The process designs are based on the literature (Note S2),^12,14,32–37^ including feedstock pretreatment, biochar/biocoke production via slow/fast pyrolysis, graphitization and post-treatment, auxiliary cooling, and wastewater treatment. BC2 and BO2 include additional hydrogen production and hydrotreating processes for upgrading byproducts into valuable H_2_ and hydrocarbons, such as sustainable aviation fuel (SAF), gasoline, and diesel. BO1 and BO2 have on-site combined heat and power (CHP) generation because they have at least 56% more process condensates available for power generation than BC1 and BC2. In BC1 and BO1, noncondensable gases (NCGs) are assumed to combust fully to CO_2_, which is accounted for as biogenic CO_2_ and separately traced in Note S3. We summarize the main design for each pathway:

1. BC1: slow pyrolysis with the main product biochar and byproduct pyrolysis vapor. Biochar goes through graphitization. Pyrolysis vapor and NCGs from graphitization are combusted to provide process heat.
2. BC2: slow pyrolysis with the main product biochar and byproduct pyrolysis vapor. Pyrolysis vapor is separated into condensed oil and NCGs through condensation. NCGs are combusted for process heat, and condensed oil is hydrotreated to hydrocarbons. Biochar goes through graphitization, and NCGs from hydrotreating and graphitization are upgraded to H_2_
3. BO1: fast pyrolysis with the main product biooil and byproduct biochar. Biochar is combusted to generate energy, and biooil goes through delayed coking. Delayed coking produces biocoke and vapors, where biocoke is sent to graphitization. Vapors are condensed into oil phase, wastewater, and NCGs. NCGs from graphitization and vapor condensation are combusted to provide process heat. Whereas oil phase from condensation is used to generate electricity. Electricity generated by CHP is assumed to be used by the graphitization and post-treatment processes.
4. BO2: fast pyrolysis with main product biooil and byproduct biochar. Biochar is combusted to provide energy, and biooil goes through delayed coking. Delayed coking produces biocoke and vapors, where biocoke is used for graphitization. Vapors are condensed and hydrotreated to hydrocarbons. NCGs from condensation, hydrotreating, and graphitization are upgraded to H_2._ Electricity generated by CHP is assumed to supply the graphitization and post-treatment processes.

Process simulations are developed in Aspen Plus v11.1.^38^ Reactor models are parametrized using literature data (Note S2). Graphitization is represented as an electric furnace with post-treatment, with an electricity demand of 4.9 kWh per kilogram of graphite, following the previous study.^14^ Catalytic graphitization uses Fe-based catalysts, and the resulting biographite is purified by HCl washing.^14^ Because recycling Fe catalysts requires challenging thermal conditions and remains nonstandard,^14^ Fe catalyst recycling was not implemented. Instead, only HCl was recycled, assuming a 95% recycling rate, consistent with industrial HCl regeneration systems.^39^ Regarding heat and electricity demand, energy recovered from pyrolysis vapors, noncondensable gases, biochar, and process condensates are used first to meet on-site process heat and/or electricity demand, as applicable to each pathway. When internally recovered energy is insufficient, supplemental natural gas and/or grid electricity are added to satisfy the remaining demand, with the associated economic and environmental burdens included in the analysis. Across all pathways, there is no surplus after meeting on-site demand. Therefore, no surplus energy export or associated economic or environmental credits are modeled. Detailed system designs are in Note S2. The resulting biographite properties are described in Note S4.

### 2.2 Life Cycle Assessment (LCA)

The functional unit is 1 kg of battery-grade graphite. The system boundary is cradle-to-gate, with foreground life-cycle inventories (LCI) derived from the process models designed above. Background LCIs are collected from ecoinvent v3.9.1.^40^ State-specific emission factors for electricity, natural gas, and diesel are collected from ecoinvent v3.9.1 ^40^ and the United States Environmental Protection Agency/National Energy Technology Laboratory (EPA/NETL).^41^ Life Cycle Impact Assessment (LCIA) methods used in this study include IPCC 2021 AR6 for climate change impact^42^ and TRACI v2.1 ^43^ for other impact categories, e.g., acidification, eutrophication, freshwater ecotoxicity, ozone depletion, respiratory effects, and smog formation. Mass allocation results are provided in the main text to attribute environmental impact to biographite, and energy allocation and system expansion results are provided in Figure S10.

### 2.3 Techno-Economic Analysis (TEA)

We conduct a discounted cash flow analysis for TEA to compute the minimum selling price (MSP) of biographite that achieves a target internal rate of return (IRR) of 10% over a 30-year project lifetime. MSP is the manufacturer’s break-even price after accounting for production costs across the plant’s life cycle, and a lower MSP indicates a more competitive product.^44^ The target IRR and project lifetime follow common assumptions of biorefinery plants.^14^ The analysis includes capital costs (CAPEX), operating costs (OPEX), tax, financing plan, and revenue from byproducts as credits. OPEX includes feedstock, utilities, catalysts, chemicals, labor, maintenance, and waste disposal. CAPEX items, financing assumptions, depreciation schedule, and construction profile follow past literature.^14,30,37^ Operating hours are set to 8000 h, a common range for energy plants. The price year is set to 2025, and all assumptions and price data are documented in Notes S6–7.

### 2.4 Uncertainty and Sensitivity Analysis

To test the robustness of the LCA and TEA results, we conduct uncertainty and sensitivity analyses on the parametrized LCA and TEA models. The models consist of price-related variables (e.g., price of materials, energy, wastes, and byproducts), LCI-related variables (e.g., material, electricity, diesel, natural gas usages in each stage, pyrolysis yields, graphitization yields, byproduct amount), and emission factor-related variables (e.g., emission factors of materials, electricity, diesel, natural gas). Ranges for the variables are collected from literature if available. Otherwise, LCI-related variables vary by ± 30%, a common uncertainty range for LCIs of biomass-derived products,^45^ and price-related variables vary by ± 20%, a commonly used range in TEA literature.^14^ The data are provided in Table S4. For each iteration in the uncertainty and sensitivity analysis, the variables are sampled together and evaluated using LCA and TEA models.

We adopted a two-stage workflow following the literature.^46^ In the first stage, all variables are screened globally using Monte Carlo simulations for each environmental impact (e.g., climate change, eutrophication, acidification, freshwater ecotoxicity, respiratory effects, and smog formation) and MSP with 1000 iterations. The five most influential variables for each impact category were identified and combined to form a shortlist of influential variables. Then, the directional effects of these influential variables on LCA and TEA outcomes were evaluated using a local one-at-a-time (OAT) sensitivity analysis. Each variable was independently varied between its lower and upper uncertainty bounds while all other independent variables were held at their deterministic baseline values. In the second stage, Monte Carlo simulations for all impact categories and TEA are run by varying the influential variables (Table S4) and 5000 iterations to obtain the range of possible outcomes. Impact categories that show minimal variations are dropped (e.g., carcinogenic human health, ozone depletion) in the following spatial analysis.

### 2.5 Societal Life Cycle Cost (sLCC)

We define societal life cycle cost (sLCC) by analogy to the literature’s definition of social costs of environmental pollutants. sLCC is the present value of the social cost attributed to producing one additional kilogram of product. It integrates TEA and monetized LCA results into a single cash-flow analysis using one social discount rate. This sLCC framework addresses limitations from past life cycle cost (LCC) literature, which directly added undiscounted environmental damage costs to MSP derived from discounted cash flows.^47^ The previous zero-discounting method for environmental burdens tends to overestimate their present value,^48^ leading to overly aggressive environmental intervention.^49,50^

We adopt the Shadow Price of Capital (SPC) approach recommended in public benefit-cost analysis^51,52^ to harmonize production and environmental damage costs. SPC is a conversion factor that expresses capital investment into its consumption-equivalent value, reflecting the social cost of spending on long-lived capital, such as that in climate technology. Subsequently, the SPC-converted capital costs, operating costs, and byproduct revenues, along with environmental damage costs, are discounted with the social discounting rate. Finally, sLCC is derived as the price ($/kg) that breaks even when all social costs and benefits are considered. More details on SPC and sLCC are described in Note S1.

The damage costs of each environmental category are based on a U.S. synthesis study^53^ (provided in Table S6). For each impact category, three scenarios were examined – low, middle, and high damage cost. The middle damage-cost scenario is shown in the main text, and the other two are evaluated in Figure S11. Particularly, climate change impact damage costs are characterized by the social costs of carbon.^54–56^ Respiratory effects damage costs capture human mortality and morbidity associated with direct particulate matter emissions (PM2.5) and particulate precursors such as nitrogen oxides and sulfur oxides.^57^ Eutrophication damage costs are quantified by economic impacts resulting from nutrient addition in surface waters.^58–61^ Acidification costs are approximated by EPA SO_2_ abatement costs^62^ due to the lack of data on the economic damages caused by acid rain. Freshwater ecotoxicity captures the economic impact of chemical and heavy metal pollutants toxic to aquatic ecosystems.^53,63^ Smog formation damage costs capture the human health damages caused by smog exposure.^64^

The damage costs are converted to $2025 USD. Then, a rising damage cost schedule^65–67^ is derived for climate change, whereas damage costs for other categories stay constant across the biographite plant cycle. This damage cost assumption is commonly adopted in literature because CO_2_ is a long-lived pollutant with an increasing marginal damage schedule,^66^ and nonclimate change categories are dominated by short-lived local pollutants whose damage is treated as static because they do not follow a clear time path.^68,69^

The sLCC of environmental impact *k* for biographite (sLCC_B,k_ in $/kg) is estimated by eq 1

1 $$\mathrm{sLC}C_{B,k}=C_{B}\left(T\right)+D_{k}\left(T\right)=\frac{{\mathrm{PV}}_{\mathrm{prod}}}{PV_{q}}+\frac{{\mathrm{PV}}_{\mathrm{env},k}}{PV_{q}}$$

Where, *T*: Project year of biographite plant, e.g., 30 years;^14^ *C*_B_(*T*): Present value of SPC-adjusted production cost per kg of biographite, depending on project year *T*; *D_k_* (*T*): Present value of environmental damage cost per kg of biographite, depending on project year *T*; *k*: One of the following impact categories—climate change, eutrophication, acidification, particulate matter formation, smog formation, ecotoxicity: freshwater.; PV*_q_*: Present value of production quantity (i.e., biographite amount across years), calculated by eq 2; PV_prod_: Present value of SPC-adjusted production cost, calculated by eq 3. PV_env,*k*_: Present value of environmental damage cost for impact *k*, calculated by eq 4.

2 $$PV_{q}=\sum_{t=1}^{T}\frac{q_{t}}{{\left(1+r\right)}^{t}}$$

3 $${\mathrm{PV}}_{\mathrm{prod}}=PV_{\mathrm{cons}}+PV_{\mathrm{inv}}^{\mathrm{adj}}=PV_{\mathrm{cons}}+s\cdot PV_{\mathrm{inv}}=\left(\sum_{t=1}^{T}\frac{C_{t}^{\mathrm{cons}}}{{\left(1+r\right)}^{t}}\right)+s\cdot \left(\sum_{t=-2}^{T}\frac{C_{t}^{\mathrm{inv}}}{{\left(1+r\right)}^{t}}\right)$$

*q_t_*: Biographite production amount at year *t*. PV_cons_: Present value of consumption cost during production, including OPEX and byproduct credits from TEA. Tax and financing are excluded from the social costs analysis as recommended by the EPA, because they are transfers between agents instead of true resource consumption;^52^ PV_inv_^adj^: Present value of capital investment cost adjusted with the SPC factor. *s*: SPC factor, e.g., 1.05–1.2.^51^ 1.1 is used in the main text, and 1.05, 1.2 were examined in SI (Note S1). PV_inv_: Present value of capital investment cost during production, including CAPEX from TEA. *C*_t_^cons^: Consumption costs during production at year *t*, e.g., OPEX and byproduct credits at year *t*. *t* = 1, 2, ···, *T* project years. *C*_t_^inv^: Investment costs during production at year *t*, e.g., CAPEX at year *t*, *t* = −2, −1, 0, 1,···,*T*, reflecting that capital investment starts from the preoperational construction period^14^ (e.g., 3 years before operation starts, consistent with TEA). *r*: Social discount rate, e.g., 1–5%. 3%^70^ is used in the main text and 1%, 5% were examined in the SI (Note S1).

4 $${\mathrm{PV}}_{\mathrm{env},k}=\sum_{t=1}^{T}\frac{D_{k,t}}{{\left(1+r\right)}^{t}}=\sum_{t=1}^{T}\frac{I_{k,t}\cdot q_{t}\cdot P_{k,t}}{{\left(1+r\right)}^{t}}$$

*D*_*k*,*t*_: Damage cost of environmental impact *k* at year *t*; *I*_*k*,*t*_: Environmental impact *k* at year *t*, derived from LCA results, e.g., kg CO_2‑eq_/kg biographite, kg N_–eq_/kg biographite; *P*_*k*,*t*_: Damage cost of environmental impact *k* at year *t* for every unit of reference pollutant, where the base value at year 1 is provided in Table S6. For climate change impact, the price of 1 kg CO_2_ (denoted as *P*_cc_) rises at a rate of 1.5% (literature range from 1.3–4% ^65–67^ ), i.e., from ith year to *i* + 1 th year, *P*_cc,*i*+1_ = *P*_cc,*i*_ × (1 + 1.015). For other impact categories, the prices stay constant. For example, in eutrophication impact, the price of 1 kg N (denoted as *P*_eu_) at year *i* and year *j* is equivalent, *P*_eu,*i*_ = *P*_eu,*j*_. The base value (*P*_*k*,1_) is used in the market graphite sLCC calculation.

The sLCC of environmental impact *k* for market synthetic or natural graphite (sLCC_M,*k*_ in $/kg) is estimated by eq 5

5 $$\mathrm{sLC}C_{M,k}=C_{M}+D_{k}^{\mathrm{market}}\left(T\right)=C_{M}+\frac{{\mathrm{PV}}_{\mathrm{env},k}^{\mathrm{market}}}{PV_{q}^{\mathrm{market}}}=C_{M}+\frac{\sum_{t=1}^{T}\left(I_{k}^{\mathrm{market}}\cdot 1\cdot P_{k,t}/{\left(1+r\right)}^{t}\right)}{\sum_{t=1}^{T}\left(1/{\left(1+r\right)}^{t}\right)}$$

*C*_M_: Price of market synthetic or natural graphite. Market natural graphite: 2.8–4.5 $/kg; market synthetic graphite: 4.2–5.3 $/kg; *D*_k_^market^ (*T*): Present value of environmental damage cost per kg of market graphite, depending on project year *T*; PV_env,k_^market^: present value of environmental damage cost for impact k for 1 kg of market graphite. The calculation follows eq 4 where *I*_*k*,*t*_ = *I*_k_^market^ and *q_t_* = 1 kg graphite; *I*_k_^market^: The environmental impact (from LCA) of market synthetic and natural graphite collected from literature (see Table S3); PV_q_^market^: Present value of market graphite quantity calculated according to eq 2 with *q_t_* = 1.

Subsequently, the difference of sLCC (ΔsLCC) between biographite and market synthetic or natural graphite for impact category *k* is calculated by eq 6. sLCC improvement of biographite compared to market graphite is defined in eq 7

6 $$\Delta \mathrm{sLC}C_{k}=\mathrm{sLC}C_{B,k}-\mathrm{sLC}C_{M,k}$$

7 $$\Delta \mathrm{sLC}C_{k}\left(\%\right)=\frac{\mathrm{sLC}C_{B,k}-\mathrm{sLC}C_{M,k}}{\mathrm{sLC}C_{M,k}}\times 100$$

Finally, the arithmetic mean of sLCC improvement (Average ΔsLCC(%)) across impact categories is derived using eq 8. This averaging is mathematically equivalent to assigning equal weight to each impact category and provides a cross-impact summary of societal cost performance. The framework can accommodate stakeholder-specific weights in future applications.

8 $$\mathrm{Average}\;\Delta \mathrm{sLCC}\left(\%\right)=\frac{1}{N_{K}}\sum_{k\in K}\frac{\mathrm{sLC}C_{B,k}-\mathrm{sLC}C_{M,k}}{\mathrm{sLC}C_{M,k}}\times 100$$

*K*: The set of all impact categories, in this study *K* = {climate change, acidification, eutrophication, respiratory effects, ecotoxicity: freshwater, smog formation}; *N_K_*: Number of impact categories, i.e., 6 in this study.

## 3 Results and Discussion

### 3.1 Environmental Impacts of Biographite

The environmental impacts (Figure 2) of producing 1 kg biographite vary with pathways. Pathways that upgrade process byproducts (BC2 and BO2) to valuable coproducts, hydrocarbons and H_2_, generally have lower environmental impacts than BC1 and BO1, which do not include upgrading (Figure 2a–d). Additionally, fast pyrolysis followed by coking generates at least 56% more condensates than slow pyrolysis (Table S7), thereby increasing CHP potential for enhancing self-energy sufficiency. With these two designs (coproduct upgrading and CHP with rich condensates), BO2 has the lowest environmental impact.

**Figure 2:**
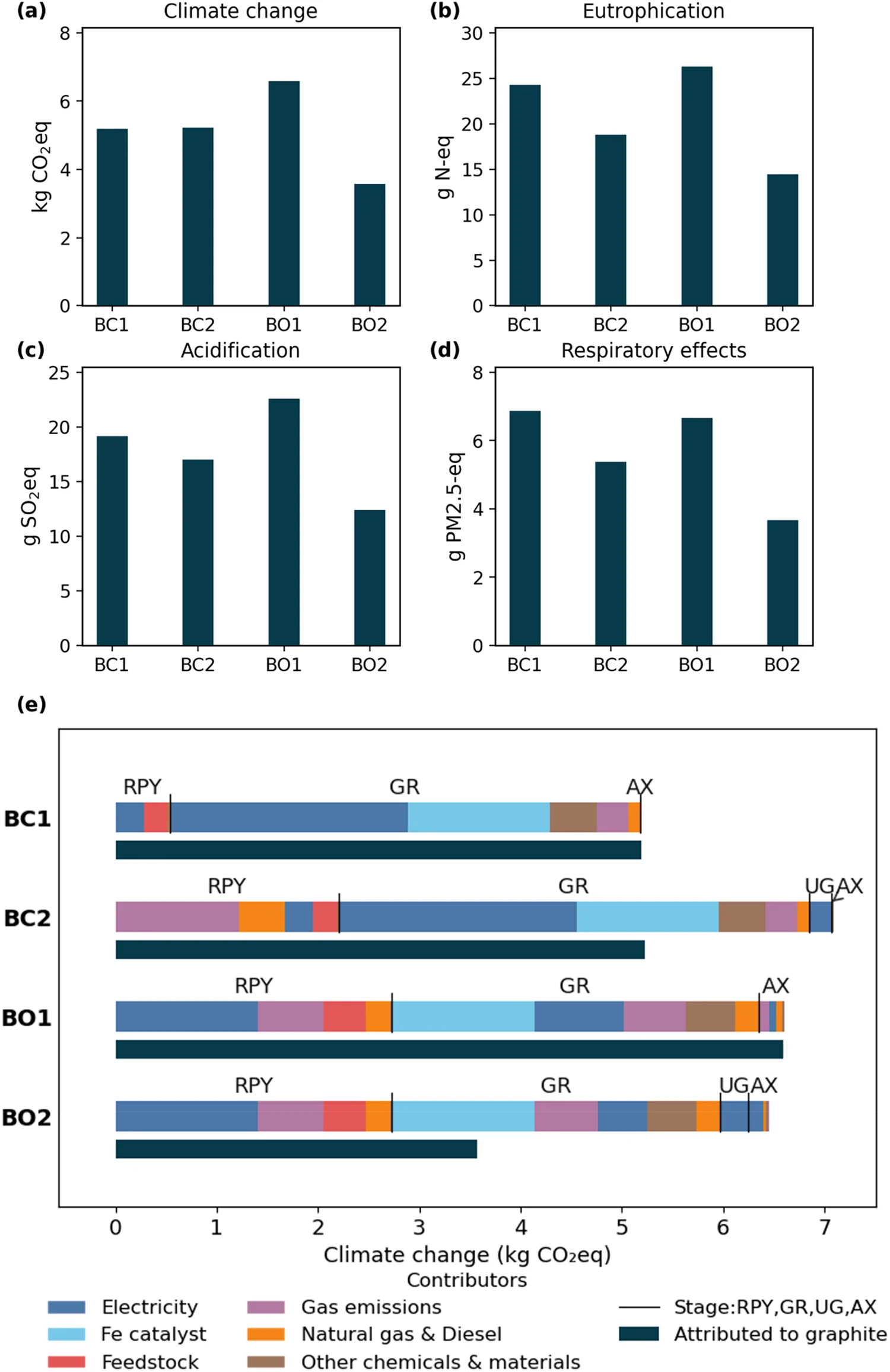
Cradle-to-gate LCA results for 1 kg biographite. Gas emissions: direct combustion emissions. Chemicals & materials (includes Fe catalyst and Other chemicals & materials), Electricity, Feedstock, Natural gas & Diesel: upstream impacts from each product supply (a)-(d) selected environmental impacts of biographite across BC1, BC2, BO1, BO2 using mass allocation. Results of other environmental impacts and allocation methods are shown in Figure S10. (e) The climate change impact, quantified by global warming potential (GWP-100 year), breakdown by energy, chemicals, materials, and direct gas emissions from different unit processes, including RPY (residue collection, pyrolysis, and coking), GR (Graphite production, including Fe-catalytic graphitization, acid washing for purification, and post-treatment such as spherization), UG (Noncondensable gas, pyrolysis vapor upgrade), and AX (other auxiliary processes, e.g., cooling, wastewater treatment). Electricity refers to the impact of grid electricity. The stacked bar charts display the results for the entire system, while the impacts allocated to graphite by mass are represented by bars labeled “attributed to graphite”. Stage breakdowns for other impact categories are provided in Figure S12.

These trends reflect a trade-off between additional process inputs and downstream burden sharing. Although upgrading byproducts requires additional energy and materials (Figure 2e), the resulting valuable coproducts can share the environmental burdens of the whole production system, and thus decrease the impacts attributed to biographite. This burden-sharing benefit is strongest in BO2, as fast pyrolysis produces more NCGs and vapors available for upgrading than slow pyrolysis.^71^ In contrast, without coproduct burden-sharing, the BO path loses its advantage, indicated by the high impact from BO1 in most categories, because it utilizes more biomass residues due to low yields and involves an energy-intensive coke production process (Figures 2e, and S12a–c,f). Figure 2 is based on mass allocation. Alternative allocation methods (energy allocation and system expansion, Figure S10) show a similar trend. Under system expansion, however, BC2 has higher environmental burdens than BC1 because the avoided burdens credited to upgraded products cannot fully offset the additional burdens from upgrading.

Across pathways, grid electricity, chemicals, and materials (e.g., Fe catalyst) are the dominant contributors, accounting for more than 50% of the environmental footprint (Figures 2e, and S12), primarily for the graphitization (in GR) and pyrolysis/coking (in RPY) processes. Direct gas emissions and upstream impact from fuel or feedstock are minor contributors, despite some exceptions. For example, gas emissions accounted for 20% of the climate change impact and natural gas and diesel accounted for 30% of smog impact in BO1, BO2, and BC2, attributing to natural gas combustion to meet additional heat requirements for pyrolysis, coking, and acid recycling, and upstream impact from natural gas production. Biomass residue (feedstock) production contributes significantly (10–25%) to eutrophication due to fertilizer use and emissions of NOx, CO, and reactive volatile organic compounds (VOCs) from agri-forestry machinery operations (Figure S12b).

In conclusion, byproduct upgrading generally reduces the environmental impacts associated with biographite, especially when byproduct yields are high, such as in BO2. The primary drivers of environmental footprints are electricity, chemicals, and materials. Additional important contributors include gas emissions from fossil-fuel combustion for climate change impact, fossil-fuel upstream production for smog impact, and biomass residue production for eutrophication and smog impact.

### 3.2 Economic Feasibility of Biographite

The economic feasibility of biographite (Figure 3) is evaluated using MSP. Overall, biographite is cost-competitive with market synthetic graphite except BO1. Market prices are based on a 2025 U.S. Geological Survey (USGS) report^72^ (details in Note S8). This competitiveness depends on pathways (Figure 3a). MSPs from BCs are 4.3–4.4 $/kg, falling at the upper end of market natural graphite.^72^ BO2 reaches $5.27 $/kg, comparable to the upper end of market synthetic graphite.^72^

**Figure 3:**
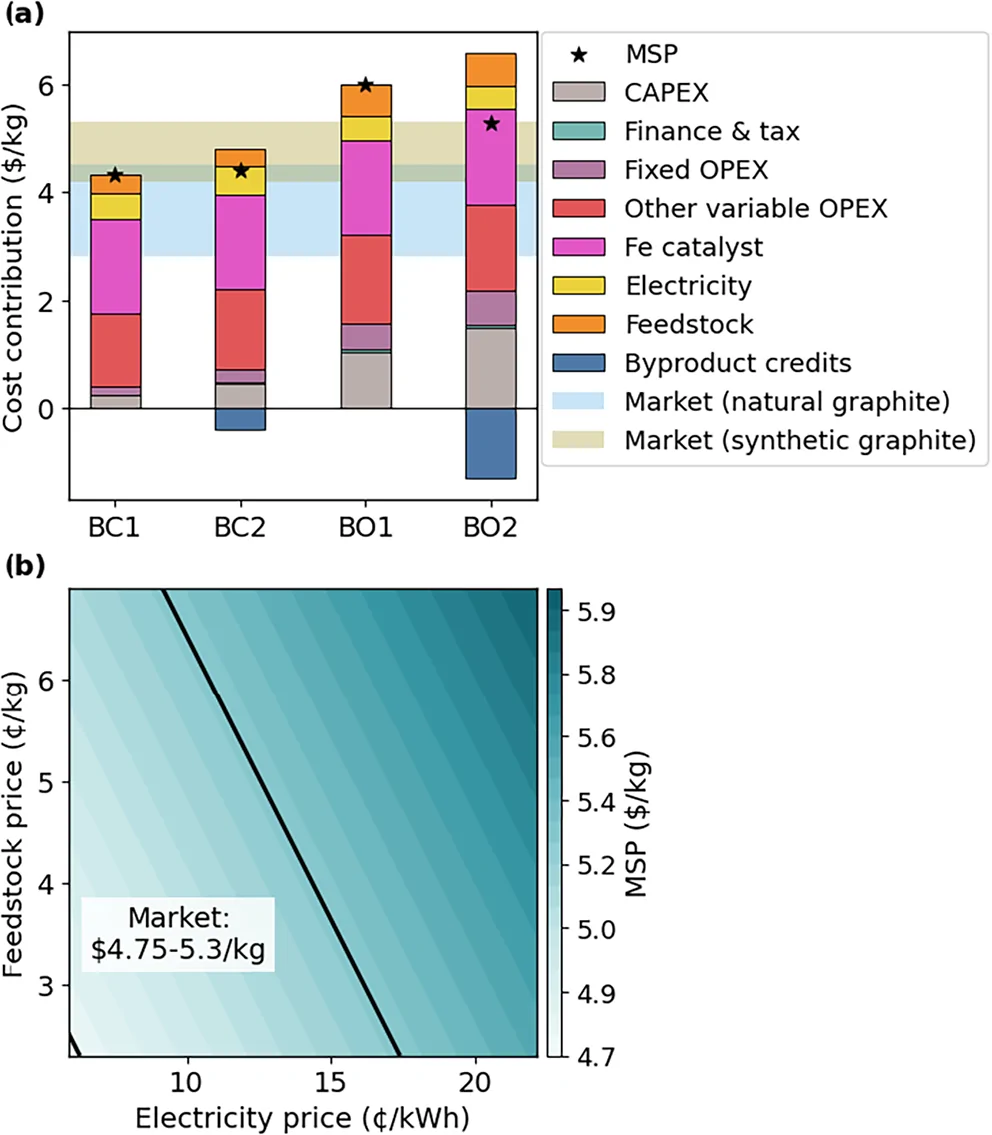
TEA results for 1 kg graphite. (a) Cost breakdown by capital (CAPEX) and operational (OPEX) costs. CAPEX: includes fixed capital investment, land costs, and working capital. Finance & tax: net contribution from debt financing, interest, and income taxes. OPEX items are listed in the following—Fixed OPEX: labor, maintenance, and insurance. Other variable OPEX: other chemicals, materials, natural gas, diesel. Fe catalyst: Fe catalyst costs, Electricity: purchased grid electricity costs, Feedstock: biomass waste costs. Byproduct credits: revenue from selling H_2_ and hydrocarbons (SAF, diesel, gasoline). Light blue and brown areas: price ranges of market natural graphite and market synthetic graphite in the U.S derived from a 2025 USGS report^72^ (details in Note S8). Market natural graphite: $2.8–4.5 per kilogram (kg) of graphite; market synthetic graphite: $4.2–5.3 per kg of graphite. (b) Variations of minimum selling price (MSP) for BO2 at different feedstock and electricity price levels. Price ranges are derived according to the prices of 2023 biomass waste^73^ and 2024–2025 electricity^74,75^ in the U.S., excluding Hawaii for visualization due to its electricity price exceeding 30 ¢/kWh. Black lines mark the price ranges for market synthetic graphite, where the lower line represents $4.75 (average price of market synthetic graphite), and the upper line represents $5.3 (upper price of market synthetic graphite).

Figure 3a further reveals the dominant cost components and factors contributing to cost variation across pathways. Fe catalyst and Other variable OPEX (e.g., other chemicals, materials, and fuels) dominate, accounting for 55–74% of the MSP. Electricity and feedstock together contribute to 17–20% of MSP. Differences between biochar and biocoke pathways are driven by CAPEX and OPEX. Specifically, BOs involve a biocoke production process that is more capital-intensive than biochar production (Table S8). The higher total OPEX for the BO than for the BC pathways is attributed to higher feedstock and maintenance (in fixed OPEX). BOs have higher feedstock costs due to lower graphite yields than those of the BCs, specifically owing to the lower biocoke yield (∼14.7%) compared to the biochar yield (∼26%). Despite the highest production costs, BO2 still achieves a competitive MSP after upgrading the vapor streams, as a lower biocoke yield is accompanied by higher-quality vapor and NCG streams that produce more coproducts (H_2_ and hydrocarbons). Generally, when comparing pathways with and without coproduct production (BO2 vs BO1; BC2 vs BC1), pathways with coproducts have higher CAPEX, but coproduct revenue offsets the increased capital investment.

We further evaluated whether the option with a higher cost but the lowest environmental impact, BO2, remains competitive under various feedstock and electricity prices (Figure 3b). Based on recent U.S. price variations in electricity and feedstock, we vary electricity prices from 5.88–22.15 US cents (¢)/kWh,^74,75^ and feedstock prices from 2.3–6.9 ¢/kg.^73^ BO2 stays below the upper bound of market synthetic graphite price ($5.3/kg) in 45% of scenarios, corresponding to when electricity is below 17 ¢/kWh across the examined feedstock range. In the Spatial Variations section, we identify locations within these price ranges.

In summary, LCA and TEA show that electricity, chemicals, materials, and feedstock are important factors in determining the sustainability of biographite. Additionally, byproduct pathways generally exhibit lower environmental and economic impacts than their no-byproduct counterparts, highlighting the importance of byproduct upgrading. Furthermore, the pathway with the lowest environmental burden (e.g., BO2) is not always the cheapest.

### 3.3 Uncertainty of the Environmental and Economic Performance of Biographite

Figure 4 evaluates the sensitivity and uncertainty of TEA and LCA results. The OAT analysis identifies electricity- and Fe-catalyst-related variables as major drivers of both MSP and environmental impacts (Figure 4a–d). Electricity price (Pc-e) strongly influences MSP, while the emission factors of grid electricity (EF-e) and Fe catalyst (EF-Fe) drive climate change impact. Variations in the consumption of Fe catalyst (LCI-Fe) and electricity for graphitization (LCI-Gre) lead to substantial fluctuations in both MSP and environmental impacts. Our findings are consistent with previous LCA^76^ and TEA^14^ studies of biomass-derived materials requiring high-temperature processing, which similarly identify electricity use as the primary driver.

**Figure 4:**
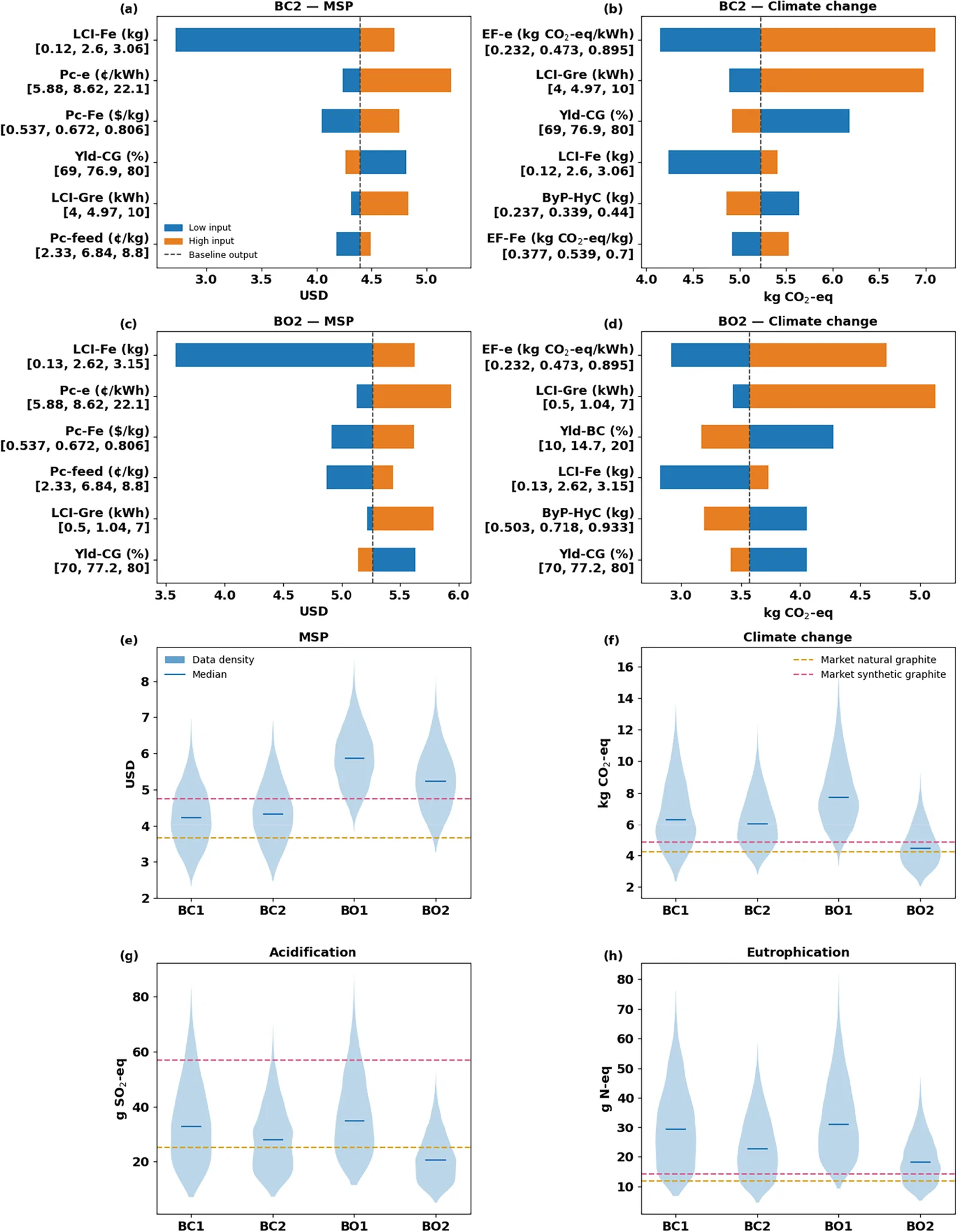
Sensitivity and uncertainty analysis of minimum selling price (MSP) and selected environmental impacts for 1 kg of graphite using mass allocation. (a–d) Top 6 most influential factors for the MSP and climate change impact of (a, b) BC2 and (c, d) BO2. Bars indicate the resulting deviation from the baseline outcome, with orange and blue representing the upper and lower input bounds, respectively. The results for other environmental impacts are shown in Figure S13. Results for BC1 and BO1 are shown in Figure S14. (e–h) The results of Monte Carlo simulations for each pathway and selected environmental impacts. The results of other environmental impacts are shown in Figure S15. LCI variables: LCI-Fe: Fe catalyst consumption (kg/kg graphite); LCI-Gre: graphitization electricity consumption (kWh/kg graphite); ByP-HyC: byproduct-hydrocarbon amount (kg/kg graphite). Price variables: Pc-e: price of electricity (cent/kWh); Pc-Fe: price of Fe catalyst ($/kg of Fe catalyst); Pc-feed: price of feedstock (cent/kg of feedstock). Emission factors (EF) units are shown according to the impact categories: EF-e: electricity environmental footprint (climate change impact unit: kg CO_2_-eq/kWh); EF-Fe: Fe catalyst environmental footprint (climate change impact unit: kg CO_2_-eq/kg). Yield variables: Yld-CG: the yield from converting biochar/biocoke to graphite (%); Yld-BC: the yield from converting biomass to biochar/biocoke. Market graphite dashed lines: ranges are marked by the average from market natural graphite (yellow) and market synthetic graphite (pink). Market graphite data are provided in Table S3. The environmental impacts of market graphite are based on ecoinvent v3.9.1,^40^ Kulkarni (2022)^22^ and Engels (2022),^79^ and market graphite price data are based on USGS (2025).^72^

Other influential factors include byproduct quantities, graphite yields, and feedstock-related variables. Greater hydrocarbon byproduct production (ByP-HyC) reduces environmental impact through increased byproduct credits, while higher biomass to char/coke yield (Yld-BC) and char/coke to graphite yield (Yld-CG) reduce the material and energy requirements per kilogram of biographite. Feedstock price variability also affects MSP. Yld-BC has a particularly strong influence on BO pathways (Figures 4d and S13–S14), especially for eutrophication, because their lower biomass-to-graphite yields make upstream biomass requirements more sensitive to changes in conversion efficiency. This observation is consistent with previous LCAs showing the importance of biomass conversion efficiency for eutrophication impact in biomass-based systems.^77,78^

Figures 4e–h and S15 show the Monte Carlo uncertainty distributions for the four biographite pathways and compare them with the average market synthetic^22^ and natural graphite^79^ (source data in Table S3). The trend is broadly consistent with the deterministic results (Figures 2 and 3). Depending on the pathways and impact categories considered, biographite can be economically and environmentally competitive with market graphite across the modeled uncertainty ranges. For MSPs (Figure 4e), the distributions for BC1, BC2, and BO2 substantially overlap with the price range of market synthetic graphite, whereas BO1 is generally less economically competitive. Biochar pathways (BC1, BC2) each have more than a 50% probability of achieving a lower MSP than market synthetic graphite.

Regarding environmental impact, biographite shows, on average, lower acidification (Figure 4g) than market synthetic graphite. This is mainly because market synthetic graphite production requires high-temperature calcination and baking, which produces substantial SO_2_ and NOx,^80^ contributing to acidification. Additionally, petroleum coke, one of the major precursors for market synthetic graphite, typically contains higher sulfur content than forestry feedstock,^81,82^ providing an additional source of SO_2_ during thermal processing. Altogether, market synthetic graphite shows a higher impact on acidification than biographite.

In contrast, biographite generally exhibits higher eutrophication and freshwater ecotoxicity (Figure S15c,e) than market graphite. Eutrophication is primarily associated with nutrient losses from biomass residue production, and freshwater ecotoxicity is strongly influenced by the Fe catalyst. For the remaining impact categories, the relative performance of biographite depends on the production pathways. For example, in climate change (Figure 4f), respiratory effects (Figure S15b), and smog formation (Figure S15f), in which electricity and natural gas are important contributors, BO2 is the most likely to achieve lower environmental burdens than market graphite due to its lowest attributed grid electricity and natural gas consumption among all biographite pathways.

In summary, the comparative conclusions for biographite pathways, as well as between biographite and market graphite, remain consistent after accounting for uncertainties. We observe that many sources of uncertainty are location-dependent (e.g., feedstock and electricity prices and energy source emission factors), suggesting the need for a spatially explicit TEA-LCA to mitigate uncertainty, as demonstrated in the following section.

### 3.4 Spatial Variations of Costs and Environmental Impacts of Biographite

Regionalized LCA and TEA reveal substantial geographic variations in biographite sustainability outcomes and its competitiveness relative to market graphite (Figures 5, and S16–S22). Biographite is generally cost-competitive with market synthetic graphite (Figure 5a,b), although its economic advantage varies by region. Based on regional groupings (Figure 5c), Central U.S. has the lowest MSPs (BC2:4.1, BO2:4.8$/kg), whereas the Pacific (California, Hawaii) and Northeast coastal (Connecticut, Massachusetts, Maine, New Hampshire, Rhode Island, Vermont) regions have the highest MSPs (BC2:5.8, BO2:6.3 $/kg). MSPs in other regions range between 4.1–5.4 $/kg and are typically below the average or at the upper price level of market synthetic graphite. The Pacific and Northeast coastal regions are exceptions, where high electricity and feedstock prices (Figure S23) drive MSPs above market prices. In contrast, biographite generally remains uncompetitive with the average price of market natural graphite ($ 3.65/kg, Figure S17a,b).

**Figure 5:**
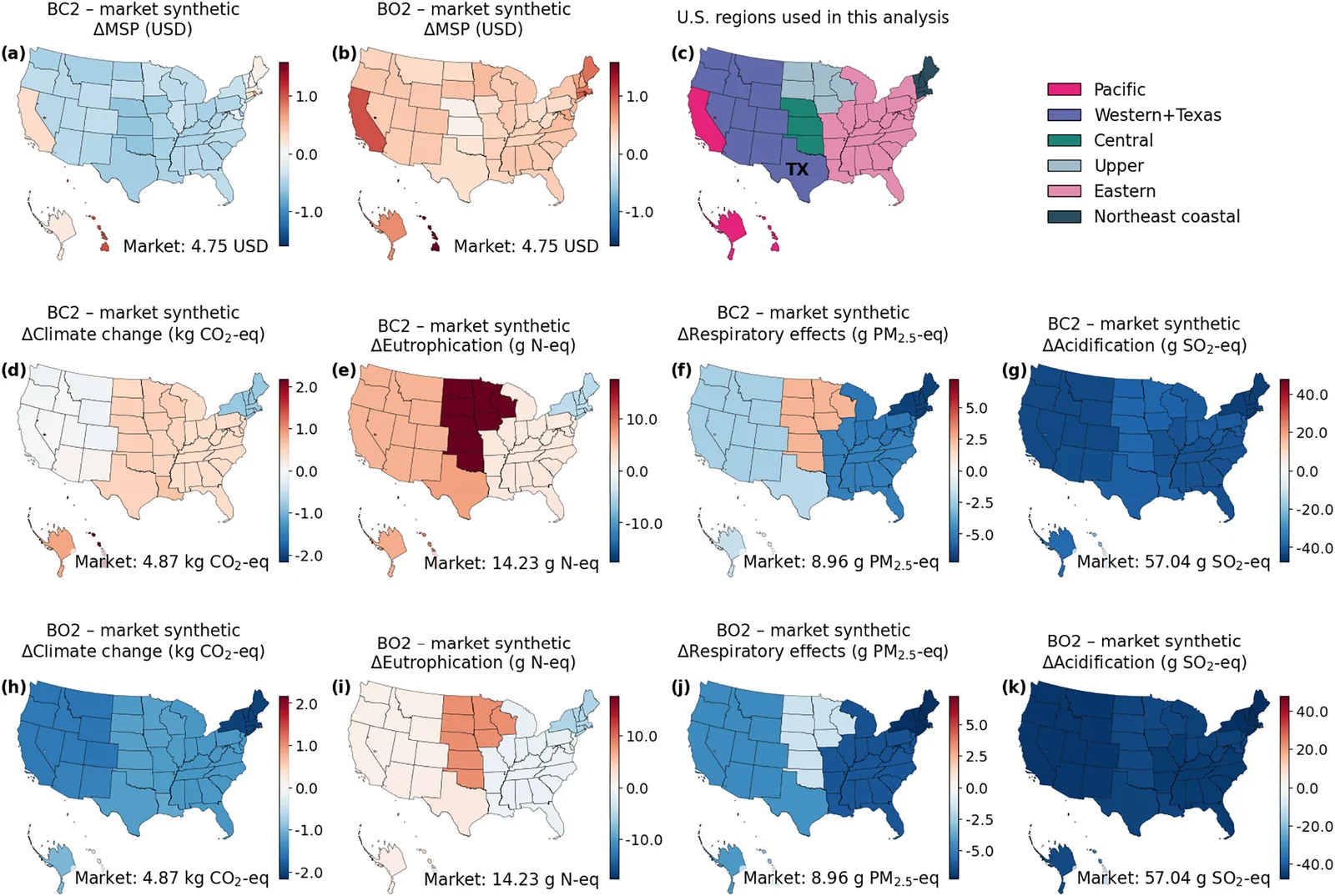
Spatial variation of Delta­(Δ) Minimum selling price (MSP) and environmental impacts for 1 kg of biographite produced from BC2 and BO2. Delta refers to the differences in MSP and environmental impact between biographite produced in each state and the average market synthetic graphite. The absolute impact values of the average market synthetic graphite are provided in each subfigure. (a, b) ΔMSP from BC2, BO2. Market synthetic graphite price range: 4.2–5.3 $/kg (c) Region definition for this study. (d–g) Delta values of each environmental impact from BC2: Δclimate change, Δeutrophication, Δrespiratory effects, Δacidification. (h–k) Delta value for each impact from BO2: Δclimate change, Δeutrophication, Δrespiratory effects, Δacidification. Other impact categories for BC2 and BO2 are shown in Figure S16. Results compared to market natural graphite are in Figures S17–S18. Additional results for BC1, BO1 compared with market synthetic graphite are in Figures S19–S20; BC1, BO1 compared with market natural graphite are in Figures S21–S22. Blue: biographite is cheaper in price, or has a lower environmental impact than market synthetic graphite. Red: biographite is more expensive or has a higher environmental impact than market synthetic graphite.

The spatial variation of environmental impact (Figure 5d–k) differs by impact category and is driven by regional energy sources. Biographite from the Central, Upper, and Eastern U.S. shows a higher climate change impact than the rest of the U.S. and can be worse than the market synthetic graphite (Figure 5d). These regions depend on fossil-based energy sources,^83^ which offset the climate benefits of using biomass feedstock, especially for BC2 (Figure 5d), which is a more electricity-intensive pathway than BO2 (Figure 5h).

Regarding eutrophication and respiratory effects, biographite from the Northeast coastal region has the lowest impact, followed by the Eastern and Western-Texas regions. Biographite made in the Northeast coastal regions most frequently shows lower impact than market synthetic graphite due to clean energy sources, characterized by hydro and nuclear power,^83^ bringing environmental advantages that outweigh the burdens associated with biomass feedstock production. Western/Texas is less favorable than the Eastern U.S. because the former relies on biomass-combusted energy and uses coal resources with a higher environmental footprint than the latter. For acidification (Figure 5g,k), spatial variation is minor because biographite production significantly reduces direct SO_2_ and NO_*x*_ compared to market synthetic graphite, regardless of regions. Similar spatial variations are observed when compared with market natural graphite (Figures S17–S18).

In summary, biographite is generally cost-competitive with market synthetic graphite except in the Northeast coastal and Pacific regions, with the Central U.S. being the cheapest. Regarding environmental impact, relative to market synthetic graphite, the Northeast coastal region is most likely to produce biographite with lower burdens, whereas the Central/Upper U.S. often leads to higher impacts. Thus, trade-offs exist: some regions produce low-cost biographite with high environmental burdens (e.g., Central and Upper U.S.), while others produce high-cost biographite with low environmental burdens (e.g., the Northeast coastal region). An indicator that integrates economic and environmental factors is essential for siting decisions for biographite plants. We address this need using sLCC in the next section.

### 3.5 Societal Life Cycle Cost (sLCC) of Biographite

Figure 6 shows the sLCC differences between biographite and market synthetic graphite (ΔsLCC) by impact category, and the average ΔsLCC across impact categories to identify where biographite is most socially attractive. Across the U.S., biographite is more societally attractive than market synthetic graphite in most states, and rarely more attractive than market natural graphite (Figures S24–S26). In the following, we focus on comparisons with the average market synthetic graphite. The largest societal gains (i.e., the most negative ΔsLCC) occur where biographite is both cheaper to produce and leads to lower damage costs relative to market synthetic graphite, e.g., in Western/Texas and the Eastern U.S.

**Figure 6:**
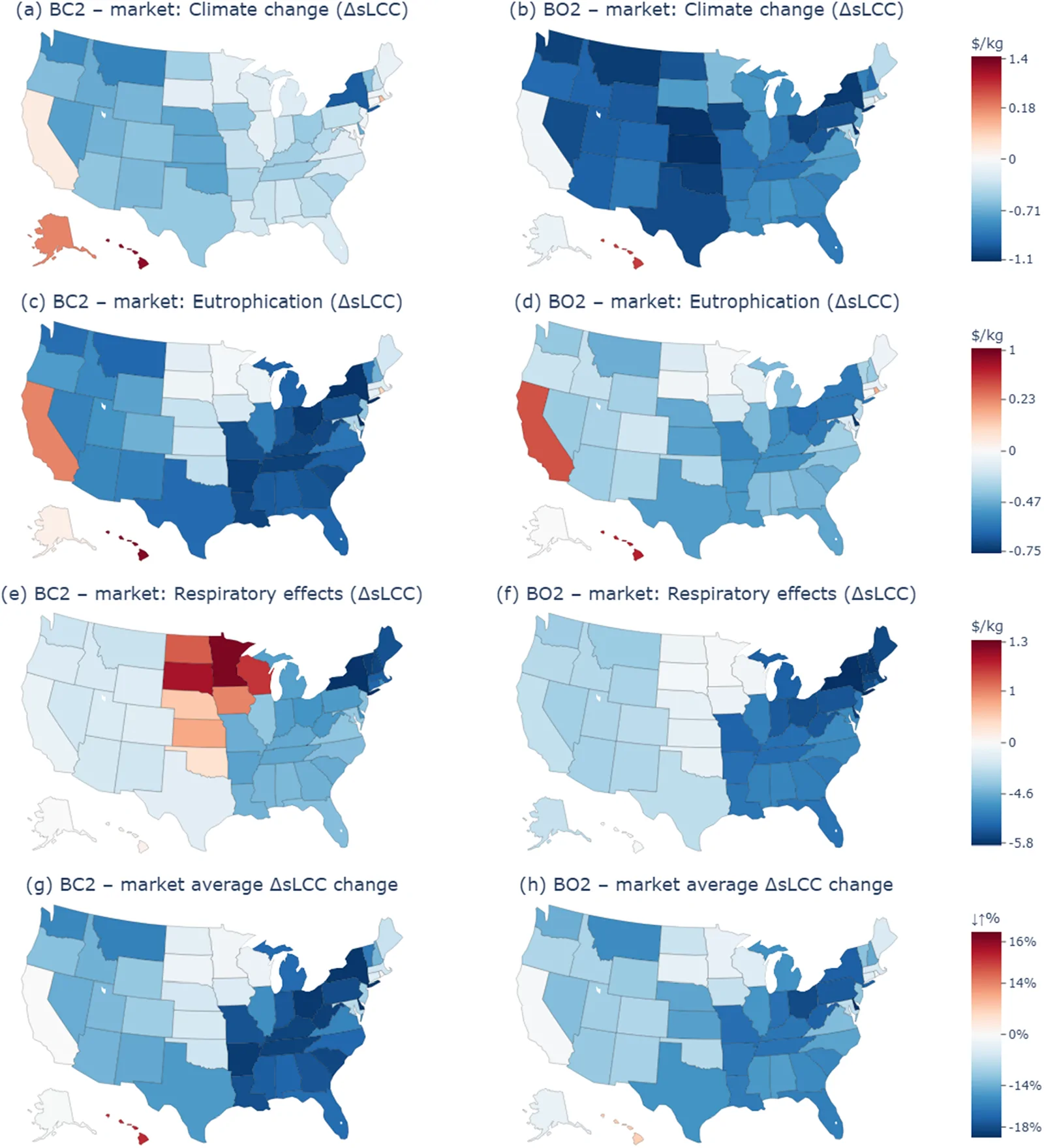
Differences between biographite’s sLCC and market synthetic graphite’s sLCC (ΔsLCC), in absolute difference ($/kg) and percentage change (%). (a–f) ΔsLCC for each environmental impact. (g, h) average ΔsLCC improvement (%) compared to market synthetic graphite. Positive (red) implies no improvement as biographite is worse than the market synthetic graphite; negative (blue) implies biographite better than the market synthetic graphite. Additional results for other impact categories are provided in Figure S24. Comparisons with the market natural graphite are provided in Figures S25–S26. Table S6 shows the damage cost for each impact category.

In contrast, in the Upper and Pacific regions, biographite often provides limited or no societal benefit relative to market synthetic graphite (Figure 6a–f), where either production or environmental damage costs are unfavorable. For example, in the Upper U.S., biographite has lower production costs than market synthetic graphite but higher environmental damage costs. The environmental disadvantages outweigh the economic advantages, particularly in respiratory effects, as particulate matter has much higher damage costs than other pollutants (Table S6). Along the Northeast coastal region, the opposite pattern dominates – high biographite production costs can diminish the environmental benefit. The Central U.S. shows mixed societal benefits across pathways and impact categories, for example, biographite sLCC is close to that of market synthetic graphite in respiratory effects, but lower in other categories, especially in the electricity-efficient pathway (e.g., BO2).

Given the distinct trends across environmental impact categories, we provide an overall summary by averaging the impact-specific ΔsLCC (Figure 6g,h). When averaged across impact categories, the Eastern regions are the most favorable in the U.S., with sLCC improvements for biographite over market synthetic graphite reaching ∼18%, followed by the Western/Texas, Central, and Northeast Coastal regions. Additionally, the Eastern regions have abundant forestry residues,^84^ making them ideal locations for biographite production. The Pacific and Upper regions gain little to no benefit from adopting biographite over market synthetic graphite.

Social cost metrics like sLCC are often sensitive to damage cost and social discounting rate assumptions.^51,52,85^ We further examine the sensitivity of sLCC to environmental damage costs, social discount rates, and the shadow price of capital. Although the absolute value of ΔsLCC varies across these assumptions, the broad spatial pattern remains consistent (Figure S11). The Eastern U.S. generally remains the most favorable region for biographite relative to market synthetic graphite, followed by the Western/Texas and Central regions. In contrast, the Pacific and Upper generally show limited societal cost advantage. These results indicate that the major regional patterns are robust to the sLCC assumptions examined, although individual locations close to the market-graphite benchmark should not be interpreted as definitively favorable or unfavorable.

In summary, we developed a spatially explicit sLCC framework that utilizes internally consistent process inventories to combine TEA and LCA. The framework coevaluates MSP and six environmental impacts, and then integrates MSP and monetized environmental damage into sLCC for regional decision-making. The framework was applied to four biographite pathways with different process designs and byproduct options. Overall, biographite pathways with byproduct production are comparable to market synthetic graphite, despite the wide price and LCI uncertainties. Sensitivity analysis reveals that sustainability outcomes are primarily driven by electricity-related factors (e.g., usage, prices, emission factors) and graphitization catalyst amount and emission factors, followed by feedstock prices and emission factors, and byproduct prices and amount.

Spatially, biographite production in most states is cost-competitive ($4.1–5.4 $/kg) with market synthetic graphite, except in the Pacific and Northeast coastal regions, due to high electricity or feedstock prices. Nevertheless, locations with economic advantages do not necessarily exhibit environmental benefits for biographite production. Therefore, sLCC was used to consider economic and environmental impacts altogether to identify locations that are societally preferable for biographite siting. The sLCC results show that most states can benefit from adopting biographite over market synthetic graphite except the Pacific and Upper U.S. Furthermore, the Eastern U.S. is the most promising siting candidate due to its low sLCC and abundance of forestry residues.

These results suggest several recommendations for the sustainable implementation of biographite in general and specifically for the U.S. From a technological perspective, upgrading byproducts to hydrocarbons and H_2_ typically improves the economic viability and environmental benefits of biographite, especially when byproducts yields are high. Furthermore, given the predominant impact of electricity, graphitization catalysts, and feedstock, improving process efficiency regarding these resources should be prioritized. For example, implementing on-site electricity generation via CHP when rich process condensates are available, improving biomass-to-graphite yield through optimized pyrolysis and graphitization conditions, and increasing the graphitization catalyst recycling rate. Establishing biographite plants in regions with low sLCC offers optimal societal benefits by balancing environmental and economic considerations, e.g., in Texas and the Eastern regions. Overall, when considering both production costs and environmental impacts, BO2 is preferred in locations with low cost and high environmental impact (e.g., central U.S.), whereas BC2 is preferred elsewhere. In contrast, when minimizing production cost alone is the primary objective, the biochar pathways are more competitive. Thus, the preferred biographite production route depends on whether the decision prioritizes production cost alone or broader societal cost.

This study has several limitations. First, we regionalized price and emission data but used the U.S. average LCIA characterization factors and environmental damage costs because consistent state-specific data are unavailable. Future work could further regionalize these parameters as suitable data becomes available. Second, the spatial analysis uses state- or region-specific point estimates and does not propagate state-specific temporal or parametric uncertainty through each spatial result. Energy prices and emission factors may vary over time, potentially affecting absolute values and the classification of locations near the market-graphite benchmark. The uncertainty and sensitivity analyses support the robustness of the broader pathway-level conclusions, while the additional sLCC sensitivity analysis shows that the major regional patterns remain consistent across the societal valuation assumptions examined. Nevertheless, individual states close to the benchmarks should not be interpreted as probabilistically classified as favorable or unfavorable. Accordingly, the spatial results are intended primarily to identify broad regional patterns and their underlying drivers. Third, the graphite quality from biochar-derived precursors needs further exploration^17,86^ due to their hard-carbon-like structures, which are challenging to graphitize. Future experimental work is necessary to explore whether additional postprocessing steps are needed to improve graphite quality derived from biochar. Finally, this study does not constrain biographite deployment according to state-specific biomass availability. As a result, the identified favorable locations reflect regional economic and environmental conditions rather than confirmed feedstock-constrained deployment potential. Future work can integrate spatially resolved biomass supply and experimentally validated feedstock suitability to evaluate resource-constrained deployment. Despite limitations, our results were based on a wide range of process inventories and yield assumptions. Thus, we expect the insights into biographite design and supply chain development to remain robust. The spatially explicit monetized LCA-TEA can be easily extended when experimental, temporal, and spatial data become available.

## Supplementary Material



## Data Availability

The deterministic LCA and TEA data underlying this study are openly available on GitHub at https://github.com/SzuHannah/Susbiographite.git.

## Notes

Societal Life Cycle Costing (sLCC) and shadow price of capital (SPC) (Note S1); process simulation (Note S2); biogenic Carbon flow (Note S3); biochar-derived and biocoke-derived biographite quality (Note S4); state-specific emission factor calculation (Note S5); techno-economic analysis—parameters (Note S6); techno-economic analysis—-discounted cash flow rate of return (DCFROR) (Note S7); -market synthetic and market natural graphite price (Note S8) (PDF)
