# Supplementary material for: A Spatially Explicit Societal Life Cycle Cost Framework for Sustainable Biographite Production in the United States
Source: Environ Sci Technol. 2026 Sep 26;60(40):28596–609. doi: 10.1021/acs.est.6c05424 (PMC13674068; doi:10.1021/acs.est.6c05424)
Supplement: Supplementary file 1 [file es6c05424_si_001.pdf]

A Spatially Explicit Societal Life Cycle Cost Framework for Sustainable Bio-Graphite Production in the United States

Hannah Szu-Han Wang[1], Jose A. Gonzalez-Aguirre[2], Sunkyu Park[2], Yuan Yao[1,3,*]

[1]Center for Industrial Ecology, Yale School of the Environment, Yale University, 380 Edwards Street, New Haven, CT 06511, USA

[2]Department of Forest Biomaterials, College of Natural Resources, North Carolina State University, 2820 Faucette Dr, Raleigh, NC 27607, USA

[3]Chemical and Environmental Engineering, Yale School of Engineering and Applied Science, Yale University, New Haven, CT 06511, USA

*corresponding author: y.yao@yale.edu

Number of Notes: 8

Number of Figures: 31

Number of Tables: 24

Table of Contents

Supporting Information – Notes 6

Note S 1 Societal Life Cycle Costing (sLCC) and Shadow Price of Capital (SPC) 6

Note S 2 Process simulation 8

Pretreatment and Char production: pyrolysis (slow pyrolysis), and condensation (Figure S 5) 8

Graphite production: Graphitization, acid washing and recycling, and bio-graphite postprocessing 9

Hydrocarbon production: 2-stage hydrodeoxygenation and fractionation 12

H_2 production 13

Wastewater management: steam generation and aqueous phase treatment 14

Cooling 14

Note S 3 Biogenic Carbon flow 18

Note S 4 Biochar-derived and Biocoke-derived bio-graphite quality 19

Note S 5 State-specific emission factor calculation 20

Note S 6 Techno-economic analysis – Parameters 21

Note S 7 Techno-economic analysis – Discounted cash flow rate of return (DCFROR) 22

Note S 8 U.S. Market synthetic and market natural graphite price 23

Supporting information – Figures 24

Figure S 1 Process flow chart for the Biochar pathway – BC1 24

Figure S 2 Process flow chart for the Biochar pathway-BC2. 25

Figure S 3 Process flow chart for the Biocoke pathway – BO1. 26

Figure S 4 Process flow chart for the Biocoke pathway – BO2 27

Figure S 5 Detailed process flow chart for unit operations in biochar production modeled in Aspen, including pretreatment, slow pyrolysis, vapor condensation 28

Figure S 6 Detailed process flow chart for unit operations in graphite production modeled in Aspen, including Fe-catalytic graphitization, acid recycling, and post-treatment, based on previous work[7] 29

Figure S 7 Detailed process flow chart for unit operations in hydrocarbon production modeled in Aspen, including two-stage hydrodeoxygenation (HDO) and fractionation 30

Figure S 8 Detailed process flow chart for unit operations in H_2 production modeled in Aspen, including reforming, water-gas-shift (WGS), and pressure swing adsorption (PSA) 31

Figure S 9 Detailed process flow chart for unit operations in Wastewater treatment modeled in Aspen, including steam generation and catalytic hydrothermal gasification (CHG) 32

Figure S 10 LCA results for 1 kg graphite across various impact categories and allocation methods for four bio-graphite pathways. 33

Figure S 11 Average sLCC change compared to market synthetic across 6 impact categories and sLCC assumptions 34

Figure S 12 LCA result breakdown for 1 kg of bio-graphite 35

Figure S 13 BC2 and BO2 sensitivity on various environmental impacts 36

Figure S 14 BC1 and BO1 sensitivity on various environmental impacts 38

Figure S 15 Monte Carlo simulation results for multiple impact categories 40

Figure S 16 Environmental impacts (from mass allocation, cradle-to-gate) for 1 kg of bio-graphite produced from BC2 and BO2 pathways and compared to the market synthetic graphite. Environmental Impact: Freshwater Ecotoxicity, Smog. 41

Figure S 17 MSP and environmental impacts (from mass allocation, cradle-to-gate) for 1 kg of bio-graphite produced from BC2 and BO2 pathways and compared to the average market natural graphite. Impact: MSP, climate change, eutrophication, respiratory effects, acidification 42

Figure S 18 Environmental impacts (from mass allocation, cradle-to-gate) for 1 kg of bio-graphite produced from BC2 and BO2 pathways and compared to the average market natural graphite. Impact: ecotoxicity, smog 43

Figure S 19 MSP and environmental impacts (from mass allocation, cradle-to-gate) for 1 kg of bio-graphite produced from BC1 and BO1 pathways and compared to the market synthetic graphite. Impact: MSP, climate change, eutrophication, respiratory effects, acidification 44

Figure S 20 Environmental impacts (from mass allocation, cradle-to-gate) for 1 kg of bio-graphite produced from BC1 and BO1 pathways and compared to the market synthetic graphite. Impact: freshwater ecotoxicity, smog 45

Figure S 21 MSP and environmental impacts (from mass allocation, cradle-to-gate) for 1 kg of bio-graphite produced from BC1 and BO1 pathways and compared to the market natural graphite. Impact: MSP, climate change, eutrophication, respiratory effects, acidification 46

Figure S 22 Environmental impacts (from mass allocation, cradle-to-gate) for 1 kg of bio-graphite produced from BC1 and BO1 pathways and compared to the market natural graphite. Impact: freshwater ecotoxicity, smog 47

Figure S 23 Electricity and biomass feedstock prices across the U.S. 48

Figure S 24 Societal life cycle costing from bio-graphite and compared to sLCC from market synthetic graphite. (scenario: middle damage cost, 3% social rate)....49

Figure S 25 Societal life cycle costing from bio-graphite and compared to sLCC from market natural graphite. (scenario: middle damage cost, 3% social rate) 50

Figure S 26 Societal life cycle costing from bio-graphite and compared to sLCC from market natural graphite. (scenario: middle damage cost, 3% social rate) 51

Figure S 27 Bio-graphite production overview across pathways. H_2 production and Hydrocarbon production only exist in BC2 and BO2. NG: natural gas. WWT: wastewater. CHP: combined heat and power generation. Proc. Cond.: process condensates 52

Figure S 28 Sensitivity analysis of various assumptions on social discounting rate, SPC, environmental damage costs for BC2 and BO2. p.p. change: percentage-point differences between scenarios. 53

Figure S 29 Dominant Natural Gas basin by state....55

Figure S 30 Electricity grid supplier by state 56

Figure S 31 Diesel PADD region by state....57

Supporting information – Tables 58

Table S 1 Billion-Ton (BT23) forest residue forecast[53] 58

Table S 2 Model Biomass Composition 59

Table S 3 Prices and environmental impacts of current market synthetic and natural graphite 61

Table S 4 Variable distributions in sensitivity analysis and Monte-Carlo Simulations....62

Table S 5 State feedstock residue prices, approximate with Industrial Wood and biomass waste price data from 2023 EIA State Energy Data System (SEDS) [79] 67

Table S 6 Societal cost of various environmental damages [3]....70

Table S 7 BC and BO paths main product yield summary, with 2000 dry t/d (≈ 83333.3 kg/h) of biomass being processed, with 416.67 kg/h N_2 providing an inert environment. Excluding catalysts and chemicals needed....71

Table S 8 Capital cost (CAPEX) from each processing stage and pathway 74

Table S 9 Model biomass structural composition derivation....76

Table S 10 Past studies on biochar and biocoke converted to bio-graphite production conditions 77

Table S 11 Proximate and ultimate analysis of biocoke obtained at 500 °C under Nitrogen atmosphere without catalyst [7] 79

Table S 12 Electricity usage of the market and bio-graphite at the graphitization stage per kg of graphite 80
Table S 13 BC paths: Modeled biochar, biooil elemental composition from slow pyrolysis and condensation 81
Table S 14 Capital investment breakdown by process sections for BC2 based on past literature [7,8,94] 82
Table S 15 TEA cash flow analysis capital investment table[7] 88
Table S 16 TEA operating costs table 90
Table S 17 TEA summary (CAPEX, OPEX, byproduct revenue) by pathway 92
Table S 18 Past studies on biochar and biocoke converted to bio-graphite material properties compared to market graphite 93
Table S 19 Modeled Pyrolysis reactions 95
Table S 20 Past studies on product yields from slow pyrolysis at around 600 ℃ for various lignocellulosic biomass 98
Table S 21 Past studies on Hydrocarbon yields and H_2 consumption for various bio-oil 101
Table S 22 Graphitization power requirement developed in Gonzalez-Aguirre et al. 2025 [7] that graphitizes 294 t/d coke 102
Table S 23 Hydrotreating H_2 and electricity consumption in past literature and BC2 103
Table S 24 Reactions in mild and deep HDO 104

Supporting Information – Notes

Note S 1 Societal Life Cycle Costing (sLCC) and Shadow Price of Capital (SPC)

Our sLCC framework is analogous to social cost metrics used in environmental economics literature. Particularly, social cost metrics such as the social cost of carbon (SCC), is the present discounted value of the economic damages caused by emitting one additional unit of a pollutant in a given year (e.g., the marginal damage caused by one additional metric ton of CO_2)[1,2]. Our sLCC framework extends this SCC concept to a unit of product by combining the social production cost of producing one kilogram of bio-graphite with the monetized environmental damages associated with its life-cycle impacts. The analogy, therefore, motivates us to examine the parameters that have commonly been identified as influential to such social metrics.

In particular, past literature suggested that a range of the following parameters should be examined[3–5]: social discounting rate, the shadow price of capital (SPC), and the environmental damage prices. Social discounting rate ranges from 1-5%, SPC ranges from 1.05-1.2, and environmental damages costs vary from low to high-cost scenarios that are shown in Table S 6 . Essentially, each factor has two levels – low v.s. high. Given that we only have three factors to vary, this setting is suitable for factorial sensitivity analysis. Factorial sensitivity analysis is a global sensitivity analysis method that utilizes the design of experiments (i.e., every combination of factor levels is examined, in our case, 2^3 combinations) to study how several inputs jointly influence a model output. To provide an overview, we examine how the social discounting rate, SPC, and the environmental damage prices affect the average percentage difference in sLCC between bio-graphite and market synthetic graphite across the six impact categories. (i.e., average ΔsLCC in %).

The results (Figure S 28) show that sLCC is most sensitive to the social discount rate, followed by environmental damage costs, while the effect of SPC is smaller. For environmental damage costs, all regions show less than 5 percentage-point change in average ΔsLCC when the damage cost scenario moves from low to high. The social discounting rate affects ΔsLCC by no more than 3 percentage-point change in BC2 and no more than 8 percentage-point change in BO2, while SPC has little effect on ΔsLCC. With the sensitivity results, we designed two additional scenarios (Figure S 11) – "high damage cost, 5% social discounting rate, SPC=1.2" and "low damage cost,

1% social discounting rate, SPC=1.05" – to verify that our bio-graphite siting conclusions stay robust regardless of these uncertainties.

Note S 2 Process simulation

Overall process for the four bio-graphite pathways (BO1, BO2, BC1, BC2) (Figure S 27) consists of the main plant and auxiliary plant. The main plant includes pretreatment of residues, biochar/biocoke production, graphitization, and hydrocarbon production. Auxiliary plant includes H_2 production, cooling, wastewater management, and combined heat and power generation (CHP). Some components are modeled in specific pathways: CHP is only modeled in BO pathways because BC pathways have much fewer process condensates (~35-55% fewer than in BOs, Table S 7) available for electricity generation, which does not justify CHP investments. Dedicated byproduct upgrade units – H_2 production and hydrocarbon production – only exist in BC2 and BO2.

The modeled biomass feedstock properties are provided in Table S 2, which represents a typical lignocellulosic waste composition that has been widely used in biomass-derived product literature [6–8], with elemental composition C (51%), H (6%), O (42%), N (0.17%), S (0.03%), and Ash (0.9%). The structural composition of the lignocellulosic biomass is estimated with the weighted average of structural compositions from pulpwood, woody residues, switchgrass, and construction and demolition waste (Table S 9). Then, the corresponding lumped chemicals that have been well studied for biomass pyrolysis modeling are derived following Debiagi (2015)[9] using Python package chemics[10] and provided in Table S 2.

1. **Biochar pathway (**Figure S 1**,** Figure S 2**)**

Pretreatment and Char production: pyrolysis (slow pyrolysis), and condensation (Figure S 5)

Overall flowsheet design is based on past literature[8,11,12]. As-received biomass is first pretreated to reduce particle size and moisture content. The dryer (S-104, modeled as a separator) reduces the incoming biomass moisture to less than 10 w.t.% [8]. The drying duty is supplied by hot flue gas recovered from pyrolysis[12], described in the following. Aligning with NREL studies, 2000 metric ton/day[8,13,14] is being processed.

The pyrolysis stage features a heating rate of around 6 °C/min, a pyrolysis temperature of 600 °C, and a residence time of 1 hour. The settings are based on typical slow pyrolysis operations and successful biochar-to-bio-graphite experimental studies (Table S 10), in which slow pyrolysis

typically refers to thermal conversion at a heating rate of less than 10°C/min[15]. The resulting biochar possesses a yield of ~ 26%, and Table S 13 depicts its elemental composition, which is expected to have less O content compared to that from lower pyrolysis temperatures[16].

During the pyrolysis stage, dry feedstock first decomposes into lumped chemicals [17,18] in R-101 (modeled with RYield). Then, the lumped chemicals undergo a series of slow pyrolysis reactions (R-102 to R-104) in an inert atmosphere provided by N_2 (flow rate: 0.5 w.t.% of the feedstock). Reactions in R-102 to R-104 are provided in Table S 19, representing primary decomposition, secondary vapor cracking, and final char conversion[19], respectively. The resulting solid-vapor mixture is then separated by S-101. Solid refers to the Biochar stream, while pyrolysis vapors compose the PY-Vapor stream.

PY-Vapor subsequently combusts to provide process heat in BC1. Whereas in BC2, PY-Vapor continues to the condensation process (Figure S 5) to recover bio-oil. Following NREL design[8], the condensation section consists of a two-stage quench system – a heavy fraction condenser (R-105) and a light fraction condenser (R-106), followed by a decanter (S-102) that separates organic bio-oil from aqueous condensates. R-105 fractionates the PY-Vapor into a heavy-oil-like stream (HFO) and a lighter and less viscous fuel oil stream (LFO). Part of the LFO is recycled as quench medium, while the remaining organic fractions are combined into the bio-oil product. After most condensable are removed, the gas stream (PY-NCG) – dominated by CO, CO_2, and light organics – is sent to a combustor to produce flue gas that provides process heat. Because a huge portion of the combustible organics in PY-Vapor is condensed into bio-oil, the heating value of PY-NCG alone is insufficient to satisfy the overall pyrolysis process energy demand. Therefore, supplemental natural gas is fired (R-108) to meet the energy requirements.

Graphite production: Graphitization, acid washing and recycling, and bio-graphite postprocessing

The process design, energy, and material usage in the graphite production follows the study developed for biocoke-derived biographite[7] (Figure S 6), consisting of graphitization, acid washing, bio-graphite postprocessing, and acid recycling. Due to the similar elemental composition of biochar and biocoke, we adopted the same graphitization process for all paths. Additionally, we

adjusted the yield and emission data based on past Fe-catalytic graphitization experiments for biochar[20,21] to account for the physical structural differences between biochar and biocoke. The acid recycling process uses spray pyrolysis, a primary commercial approach for regenerating HCl[22]. The properties of biocoke and biochar that went through the graphitization step are provided in Table S 11 and Table S 13, respectively. The resulting bio-graphite from biocoke is assumed to have the first-cycle discharge capacity of 345 mA h/g[23], while biochar-derived bio-graphite has 330 – 355 mA h/g[24–26], and other properties compared to market graphite are shown in Table S 18. While both BC and BOs can produce battery-grade graphite, BOs generally perform better.

The graphitization process is described herein. Biochar/biocoke from char/coke production undergoes a pretreatment process[7] (S-301-S-303) before graphitization. A gyratory crusher (S-301) is assumed to reduce biochar/biocoke to a median particle size (D_{50}) of 2.5 mm. The particles continue to a screening stage, modeled with two consecutive vibratory sieves (S-302, S-303), producing a final particle size of D_{50} = 26 μm to improve the homogeneity and contact between the Fe catalyst and biochar/biocoke particles during graphitization. Around 95.5% of the biochar/biocoke passed the sieving, and 4.5% were lost as fines. Subsequently, biochar/biocoke continues to the graphitization furnace. Power requirements for this pretreatment process were calculated following our previous work[7] and summarized in Table S 22.

Catalytic graphitization is performed using iron powder as the catalyst. Fe(II) compounds have been widely studied in promoting the formation of high-quality graphite crystals from biomass-derived feedstocks. The mixture of solid and Fe catalysts follows the successful literature on biographite production. For BOs, the iron catalyst is mixed with biocoke at an iron-to-fixed carbon mass ratio of 2.5[7] prior to thermal treatment. For BCs, the iron catalyst and biochar are mixed at a mass ratio of 2:1[27].

The graphitization step is modeled with a continuous rotary kiln furnace (R-301) operating under a N_2 atmosphere at 1500 ℃, such that a carbon yield of 99% can be achieved[23]. The resulting products are recorded in Table S 7, where biochar/biocoke to bio-graphite yield is roughly 77 w.t.%, aligning with past literature summarized in Table S 10. Gaseous byproducts composed of CO, H_2, CH_4, and CO_2, which were routed to H_2 production in BC2/BO2 and fully combusted as process heat in BC1/BO1.

Following graphitization, a solid quenching step is conducted in a rotary cooling drum (E-301), where cooling water is used to reduce the bio-graphite temperature from 1500 ℃ to 120℃. After cooling, the solid mixture is washed using a 30% aqueous hydrochloric acid solution to remove residual iron. The washing step is modeled with a stirred-tank, liquid-phase adiabatic reactor maintained at 50 ℃ (R-302), where Fe reacts with HCl to produce H_2 and ferrous chloride ($FeCl_2$). The effluent is then cooled to 30 ℃, and centrifuges and filtered to yield bio-graphite with a purity of 99.95 wt%. The resulting spent aqueous $FeCl_2$/HCl stream is directed to the acid recycling system.

The bio-graphite post-processing unit comprises three main steps: drying (E-302), milling, and shaping (S-305). Drying is performed using an air dryer operating at 85 °C to reduce the moisture content to below 0.05 wt%. Following drying, the bio-graphite is shaped into spherical particles using an impact mill. This spherical bio-graphite is the final product of the system, which can be used as an anode or electrode material for downstream applications. The full electricity consumption of the graphitization step is based on literature [7] and recommendations from experts, and is reported in Table S 22.

The acid recycling system is modeled with pyrohydrolysis, a commonly used approach to regenerate HCl from spent HCl/$FeCl_2$ liquor. The system consists of a preconcentrator (S-306) that reduces water content, and a spray roaster (R-303) that regenerates HCl. Spray roaster operates at 600-800℃ (700℃ is used in this study), and HCl is regenerated through the following reaction: $4FeCl_2 + 4H_2O + O_2$ à $2Fe_2O_3 + 8HCl$. Fe_2O_3 can be sold, but for a conservative estimation, we did not assign economic value.

HCl gas subsequently continues to an absorber (S-308), along with makeup water, to compose the regenerated HCl with a concentration of around 15wt%. Makeup HCl is then added to recycle back to the graphite washing system. The absorber purges are diverted to a neutralizer (R-304, R-305), and the resulting brine continues to wastewater treatment, and the $Fe(OH)_3$ is disposed. Overall, 95% of HCl is recycled, aligned with industry standards.

Byproduct upgrading

BC2 employed two upgrade units: H_2 production that upgrades non-condensable gas (NCG) and a hydrotreating and fractionation unit that upgrades condensed biooil from slow pyrolysis.

Hydrocarbon production: 2-stage hydrodeoxygenation and fractionation

Two-stage hydrodeoxygenation (HDO)

We modeled hydrocarbon production as a two-stage HDO (Figure S 7), followed by fractionation, based on past literature[8,28,29]. The process consists of mild HDO and deep HDO. Specifically, bio-oil from slow pyrolysis/condensation and H_2 are pressurized and preheated before entering mild HDO. Mild HDO is designed for low-severity hydrotreating to stabilize bio-oil by hydrogenating the most reactive oxygenates (e.g., carbonyl/carboxyl functionalities) and partially deoxygenating the oil, which reduces bio-oil acidity[8,29]. Mild HDO reactor (R-201) is set at 200 ℃ (typically 180 - 250℃), 10 MPa, and NiMo/CoMo catalysts are assumed[8,30]. Subsequently, the outlet stream enters the 1st separator (S-201), where the Mild HDO effluent is cooled and flashed into gas, aqueous, and organic phases. Part of the gas phase is returned as recycled H_2 and, along with the organic phase, continues to the deep HDO reactor (R-202). Deep HDO is designed for high-severity hydrotreating to reduce Oxygen content in bio-oil down to <2 w.t.%[8,31], and the reactor is set at 400 ℃ (typically 350 – 425 ℃), 15 MPa, and NiMo/CoMo catalysts are assumed[8,29]. Under these severe hydrotreating conditions, hydrodeoxygenation occurs alongside hydrogenation and hydrocracking reactions, which can convert a fraction of the heavier upgraded oil into lighter-range hydrocarbons [8,31]. The effluent from deep HDO is then separated into gas, aqueous, and oil phases with a separator (S-202). The gas streams from both separators are collected and cleaned up before being purged out (called HDO-NCG) to the H_2 production section. The aqueous streams from both separators are collected (called HDO-WWT) and treated in the wastewater section. The organic product (called HDO-OIL) is sent to the fractionation stage. Reactions in mild and deep HDO reactors are provided in Table S 24. The modeled H_2 usage is approximately 0.056 kg H_2/kg biooil, within the literature range of 0.025 – 0.096 kg H_2/kg biooil (Table S 23). All H_2 is supplied by internal H_2 production.

Fractionation

HDO-OIL from the 2-stage HDO process continues to the fractionation section[8] (Figure S 7), which consists of a conventional two-column distillation setup (S-205, S-206) to generate three

hydrocarbon streams: gasoline, sustainable aviation fuel (SAF), and diesel. In the first column, the HDO-OIL is flashed and fractionated into a light gasoline-range overhead (called GASL) and a heavier middle-distillate bottoms stream (called HEAVY). The HEAVY stream is then fed to a second column, which performs a kerosene split to recover a SAF-range product (called SAF) and a diesel-range product. This fractionation setup is consistent with biorefinery practices. It leverages the fact that the boiling-point distribution of HDO-OIL lies largely within the gasoline/jet/diesel ranges and can be separated using standard atmospheric fractionators without additional chemical reactions. The resulting bio-oil to hydrocarbon yield is 66.6%, which is similar to previous literature that also hydrotreats biooil with 20-30% oxygen content (Table S 21). The hydrocarbon distribution among gasoline, diesel, SAF-range products is: 60.5%, 8.23%, and 31.25%, respectively.

H_2 production

H_2 production process (Figure S 8) utilizes steam from wastewater treatment, NCGs from hydrotreating (HDO-NCG) and graphitization (GR-NCG), and Ni/Al_2O_3 catalyst[7] to produce H_2 for on-site hydrotreating demand or export to the market. This design is based on past literature[7,30,32,33], consisting of steam reforming, water-gas shift (WGS), and pressure swing adsorption (PSA). Specifically, NCGs and steam are pressurized (C-401) to 16 bar (typically 10-30 bar[8,34]) and preheated before entering the reformer unit . The reformer (R-401) operates at ~ 909 °C [33](typically 800-900°C[14,34]), 16 bar, where the inlet stream is converted to an outlet stream consisting of H_2, CO, CO_2, CH_4, and H_2O. Subsequently, the stream, along with steam generated from wastewater treatment (WWTSTM-Steam), passes through a 2-stage WGS with catalysts: high-temperature WGS (R-402) and low-temperature WGS (R-403)[33]. High-temperature (310-450 °C[32]) WGS converts CO, H_2O into CO_2, H_2; low-temperature (200-250°C[32]) WGS further reduces the rest of CO into CO_2 and H_2. After WGS, the stream continues to PSA (S-402), which aims to separate high-purity H_2 from CO, CO_2, CH_4, and N_2. The PSA outputs include H_2, off-gas, and condensates. Condensates are sent to the wastewater treatment unit, off-gas is subsequently combusted to provide process heat[30], and H_2 is sent to hydrotreating or sold if excess.

Auxiliary processes

Wastewater management: steam generation and aqueous phase treatment

Wastewater management (Figure S 9) treats aqueous effluents from pyrolysis, hydrotreating (HDO), graphitization, and H_2 production. The design is based on past literature[8,30,35,36], consisting of a wastewater boiler, a catalytic hydrothermal gasification (CHG) unit, a reverse osmosis (RO) unit, and a regenerative thermal oxidizer (RTO).

The aqueous phase from the pyrolysis quench loop (PY-WWT) is typically routed to a waste-heat boiler to recover energy as process steam[30]. PY-WWT is first sent to a waste-heat boiler system (S-501), where it generates steam for water-gas shift reactors in H_2 production[30]. Subsequently, the remaining PY-WWT from this boiler, together with makeup water, is routed to the second boiler (S-502), which produces higher-quality steam, leveraged by the reformer unit in H_2 production.

The wastewater streams from HDO and H_2 production together have a chemical oxygen demand (COD) of 373 g/L. Given the high COD, these streams are sent to a CHG unit that removes dissolved organic matter and reduces the toxicity in wastewater[37]. Before CHG, a flasher (S-503) was used to flash out light organics. Then, the stream continues to CHG (R-501), which operates at 400 ℃, 21 MPa, with Ni/Al_2O_3 catalysts. The CHG effluent is cooled and separated into gas and aqueous phases (S-504). The gas phase is rich in fuel gas (i.e., CH_4), and is therefore sent to combustion to recover as process heat. The aqueous phase mainly consists of water and is routed to the RO unit (S-505) together with the wastewater from graphitization (Gr-WWT).

The RO unit removes residual organics, producing a water stream that is suitable for internal reuse. The RO concentrate, along with blowdown from the steam boiler and any remaining light organics is ultimately directed to the RTO unit (R-504). RTO is the final module that removes residual volatile organics, achieving more than 95-99% removal efficiency of VOCs and hazardous air pollutants[38], and releasing flue gas that is subsequently cooled and vented.

Cooling

Flue gases from H_2 production and the regenerative thermal oxidizer (RTO, from wastewater treatment) are cooled by a plant-wide cooling water (CW) loop. Following NREL and PNNL

design cases[8,13], the cooling water is supplied by a mechanical-draft cooling tower operated over a 90-110°F window, meaning that cooling water leaves the tower at 90°F and returns from the processes at 110°F before being re-cooled. Total heat rejected to the CW loop, Q_{tot}(kW), is taken from Aspen simulations. In the following, we describe the calculation for the amount of makeup water needed and the cooling-tower electricity demand.

Cooling-water circulation requirement

The required CW circulation rate ($\dot{m_{cw}}$) is calculated by the following energy balance:

$$\dot{m_{cw}} = \frac{Q_{tot}}{c_{p,w}\Delta T_{cw}}$$

Where,

$c_{p,w}$: specific heat of liquid water, ≈4.18 kJ kg^{-1}K^{-1} at 20°C[39].

ΔT_{cw}: cooling water temperature rise, equals to 110°F – 90 °F= 20 °F ≈11.1 K

For volumetric flow:

$$\dot{V}_{cw} = \frac{\dot{m}_{cw}}{\rho_w}$$

Where,

ρ_w: the density of liquid water (≈995 kg m^{-3} at ~30°C)[39]

Cooling-tower makeup water

Cooling-tower makeup water is determined by evaporation losses (due to cooling), blowdown (intentional discharge to control salt buildup), and optionally, drift (small droplet losses). Evaporation ($\dot{m}_{evap}$) is estimated from the portion of cooling duty rejected as latent heat:

$$\dot{m}_{evap} = \frac{f_{lat}Q_{tot}}{h_{fg}}$$

Where

h_{fg}: latent heat of vaporization of water at representative tower conditions[40], e.g., h_{fg}=2429.8 kJ kg^{-1} at 30°C

f_{lat}: latent fraction of tower heat rejection, e.g., 0.75 – 1[41]

Blowdown ($\dot{m}_{blowdown}$) is calculated from $\dot{m}_{evap}$ and cycles of concentration (CoC):

$$\dot{m}_{blowdown} = \frac{\dot{m}_{evap}}{CoC - 1}$$

Where CoC is typically 4 or 5[41,42]

Subsequently, makeup water ($\dot{m}_{makeup}$) is:

$$\dot{m}_{makeup} = \dot{m}_{evap} + \dot{m}_{blowdown} + \dot{m}_{drift} \approx \dot{m}_{evap} + \dot{m}_{blowdown}$$

Where $\dot{m}_{drift}$ is optional; if included, previous NREL report estimates drift as 0.2% of the circulating CW flow [42](i.e.,$\dot{V}_{drift} = 0.002\dot{V}_{cw}$)

Cooling-tower electricity demand

Cooling-tower electricity demand (P_{elect}) consists of pumping power (P_{pump}) and cooling-tower fan power (P_{fan}).

$$P_{elect} = P_{pump} + P_{fan}$$

P_{pump} is calculated with:

$$P_{pump} = \frac{Q_{tot} g H}{\eta_{pump} \eta_{motor}}$$

Where,

g: gravity constant=9.81 m/s^{-2}

H: pump head in meters, calculated with $H = \frac{\Delta P}{\rho_w g}$, and ρ_w=995 kg/m^3 is water density, ΔP=179,352 Pa is cooling water pump head[43]

η_{pump}: 0.65, typical centrifugal pump efficiency[44]

η_{motor}: 0.9, typical industrial motor efficiency [45]

On the other hand, cooling-tower fan power (P_{fan}) is estimated as a fraction of the heat rejected:

$$P_{fan} = \alpha_{fan} Q_{tot}$$

Where

α_{fan}: fan power intensity of a cooling tower=0.01[46,47]

2. **Biocoke pathway (**Figure S 3**,** Figure S 4**)**

Biocoke to bio-graphite pathways are based on the literature[7]. The main differences between biochar pathways and biocoke pathways lie in the graphite precursor and the inclusion of combined heat and power generation (CHP). In BO1 and BO2, biocokes are used as graphite precursors. Detailed unit operations are provided in the supporting information of Gonzalez-Auirre et al. (2025)[7]. Simplified overviews for BO1 and BO2 are provided in Figure S 3 and Figure S 4, respectively. In BO1, all non-condensable gases are combusted for process heat, whereas in BO2, non-condensable gases are routed to H_2 production. Additionally, the coke vapor from coking is routed to CHP in BO1, whereas in BO2, the coke vapor is upgraded to hydrocarbons.

Note S 3 Biogenic Carbon flow

Biogenic carbon flow is separately traced for all pathways. BC2's biogenic carbon flow is provided below as a representative case. HydroC refers to hydrocarbon. Unit: kg/sec

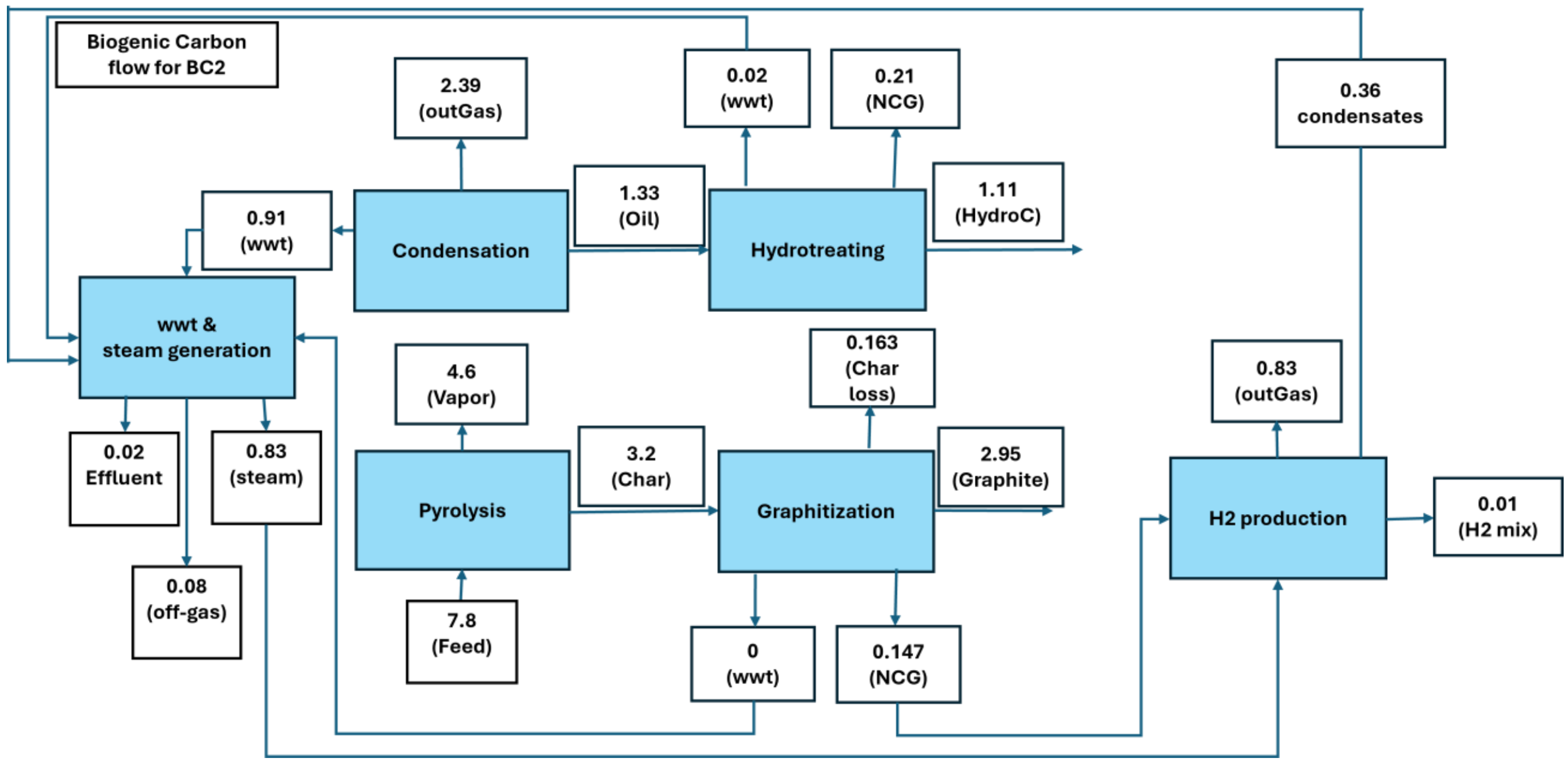

Note S 4 Biochar-derived and Biocoke-derived bio-graphite quality

Lignocellulosic biomass has been successfully converted into bio-graphite through iron-catalytic graphitization via biochar or biocoke as the intermediate precursor. The resulting bio-graphite performance varies, and Table S 18 provides a summary. We describe each property herein:

1. Specific Capacity: Market synthetic graphite typical possess 300 – 360 mAh/g. Biocoke-derived bio-graphite has reached 370 mAh/g, and biochar-derived bio-graphite has reached 357 mAh/g.
2. Initial Coulombic Efficiency (ICE): Industrial targets for battery anodes are more than 90%. Earlier biochar-derived bio-graphite struggled with low ICE (67-77%) due to high specific surface areas, optimized biocoke and sieved biochar precursors can now achieve 90.2 – 92.3% ICE, potentially matching commercial requirements. Where sieved biochar study observed that sieved biomass to 63 – 105 µm or 105 – 149 µm can achieve ICE 90.7% and 92.3%, respectively.
3. Crystallinity and Purity: Market synthetic graphite has ~94% graphitization degree, whereas bio-graphite can achieve a graphitization degree of more than 91%. After rigorous acid washing, the carbon purity reaches 99.95%, meeting the requirements for battery applications.

Note S 5 State-specific emission factor calculation

Emission factors for state-specific energy source – electricity, natural gas, and diesel – was derived as follows, and the assigned energy source for each state is visualized in Figure S 29, Figure S 30, and Figure S 31:

1. Electricity: ecoinvent v3.9.1 regional electricity location code follows North American Electric Reliability Council (NERC) sub-regions geospatial code. Particularly, subregions for U.S. electricity grid include US-ASCC, US-HICC, US-MRO, US-NPCC, US-RFC, US-SERC, US-TRE, US-WECC - covering Alaska, Hawaii, Midwest/Great Plains, New England/New York, Mid-Atlantic/Great Lakes corridor, Southeast/Florida, Texas, Western Interconnection, respectively. We mapped each state to its corresponding subregions using geometries defined from ecoinvent official document [48].
2. Natural gas: there are 14 basins across the U.S., including Anadarko, Appalachian, Arkla, Arkoma, East Texas, Fort Worth, Green River, Gulf, Permian, Piceance, San Juan, South Oklahoma, Strawn, and Uinta. Using facility-level records from the NETL dataset [49] that link each facility to its upstream basin, we assign a representative basin to each state by taking the basin that appears most frequently among all gas-supply facilities located within that state. Subsequently, the emission factors of natural gas (per MJ) supply from each basin are calculated for each state using the basin-specific LCIs from the NETL-EPA collaborative repository [49].
3. Diesel: Petroleum Administration for Defense Districts (PADDs) are used to spatialize diesel emission factors. There are 5 PADD regions: PADD1, PADD2, PADD3, PADD4, PADD5, covering states in the East Coast, Midwest, Gulf Coast, Rocky Mountain, and West Coast, respectively. The state to PADD region mapping is officially provided in many EIA analysis [50] and provided in the NETL repository. Emission factors for each PADD regions are subsequently calculated for each state.

Note S 6 Techno-economic analysis – Parameters

Parameters in TEA follow existing literature on nth-of-a-kind biorefinery from NREL and PNNL. The discounted cash flow analysis includes capital costs (CAPEX), operating costs (OPEX), tax, and financing plans. Land and working capital are assumed to be recoverable at the end of the project lifetime.

Table S 8 shows the summary of the capital cost data by processing area, which is calculated by equipment purchase cost multiplied by installation factor. A breakdown list of equipment purchase cost is shown in Table S 14, where the capital equipment lists follow previous literature and are adjusted to account for the capacity and stream flows from our bio-graphite plant.

Main terms that are used in these tables:

(1) Total installed cost: for each scenario, the total installed cost is the summation of all rows in "Installed capital cost" column, as in Table S 8
(2) Total direct cost (TDC): TDC is the summation of total installed cost, warehouse cost, site development cost, and additional piping cost, which are all presented as a percentage of inside-battery-limits (ISBL) installed capital, as specified in Table S 15.
(3) Total indirect cost (TIC): TIC is the summation of field cost, home office and construction costs, contingency, prorated expenses, and other indirect costs, which are calculated as percentages of TDC, and are specified in Table S 15
(4) Fixed Capital Investment (FCI): the summation of TDC and TIC, i.e., FCI = TDC + TIC

Overall,

$$CAPEX = FCI + working\ capital + land\ cost$$

OPEX are provided in Table S 16, including variable operating costs: feedstock, utilities, catalysts, and chemicals price data; fixed operating costs: labor costs, maintenance, and property insurance. A summary of OPEX, CAPEX by bio-graphite pathway is provided in Table S 17

Note S 7 Techno-economic analysis – Discounted cash flow rate of return (DCFROR)

With discount rate (or target internal rate of return (IRR)) r, the net present value (NPV) of a product at price P:

$$\mathrm{NPV(P)} = \sum_{\mathrm{t=t_0}}^{\mathrm{T}} \frac{\mathrm{CF_t(P)}}{\mathrm{(1+r)^t}}$$

Where $\mathrm{CF_t(P)}$ is the net cash flow in year t that depends on the product price P

$$\mathrm{CF_t(P) = P \times Q_t + B_t - C_t^{CAPEX} - C_t^{OPEX} + F_t - T_t(P)}$$

Where,

$\mathrm{Q_t}$: bio-graphite production at year t (unit: kg)

P: bio-graphite product price (unit: $/kg)

$\mathrm{B_t}$: by-product revenue at year t (unit: $)

$\mathrm{C_t^{OPEX}}$: operating costs at year t (OPEX, unit: $)

$\mathrm{C_t^{CAPEX}}$: capital costs at year t (CAPEX, unit: $)

F_t: net financing cash flow, including debt proceeds, principal repayment, and interest (unit: $)

$\mathrm{T_t(P)}$: income tax payment at year t, depending on product revenue (P)

Thus, minimum selling price (MSP) is defined by:

$$\mathrm{MSP = P^* \; s.t. NPV(P^*)} = \sum_{\mathrm{t=t_0}}^{\mathrm{T}} \frac{\mathrm{P^*Q_t + B_t - C_t^{CAPEX} - C_t^{OPEX} + F_t - T_t(P^*)}}{\mathrm{(1+r)^t}} = 0$$

Where r is target internal rate of return (IRR) = 10% . Practically, the minimum selling price $\mathrm{P^*}$ was solved numerically using a root-finding algorithm[51] such that the project NPV equals zero at the target IRR 10%.

Note S 8 U.S. Market synthetic and market natural graphite price

A range of graphite prices was derived using data from the latest U.S. Geological Survey (USGS) report in 2025 which summarized graphite data from 2021-2022[52]. For natural graphite, USGS reported that uncoated spherical graphite has a price range of $2,800 – 3,000 / metric ton (t), and natural spherical purified graphite imported to the U.S. has a price range of $3,930 – 4,480/t. Based on these data, we use a price range of $2.8–4.5/kg for battery-grade natural graphite.

For synthetic graphite, the USGS[52] reported that imported synthetic graphite battery material is priced at $5,520/t in 2021 and $5,130/t in 2022. When including non-battery material, the U.S. synthetic graphite trade values in 2022 suggest that imported synthetic graphite has a unit value $4,210 /t, and exported synthetic graphite is $5,820/t. Thus, we use a price range of $4.2–5.3/kg for battery-grade synthetic graphite. $5.3/kg is the rounded average of imported synthetic graphite prices from 2021 and 2022.

Supporting information – Figures

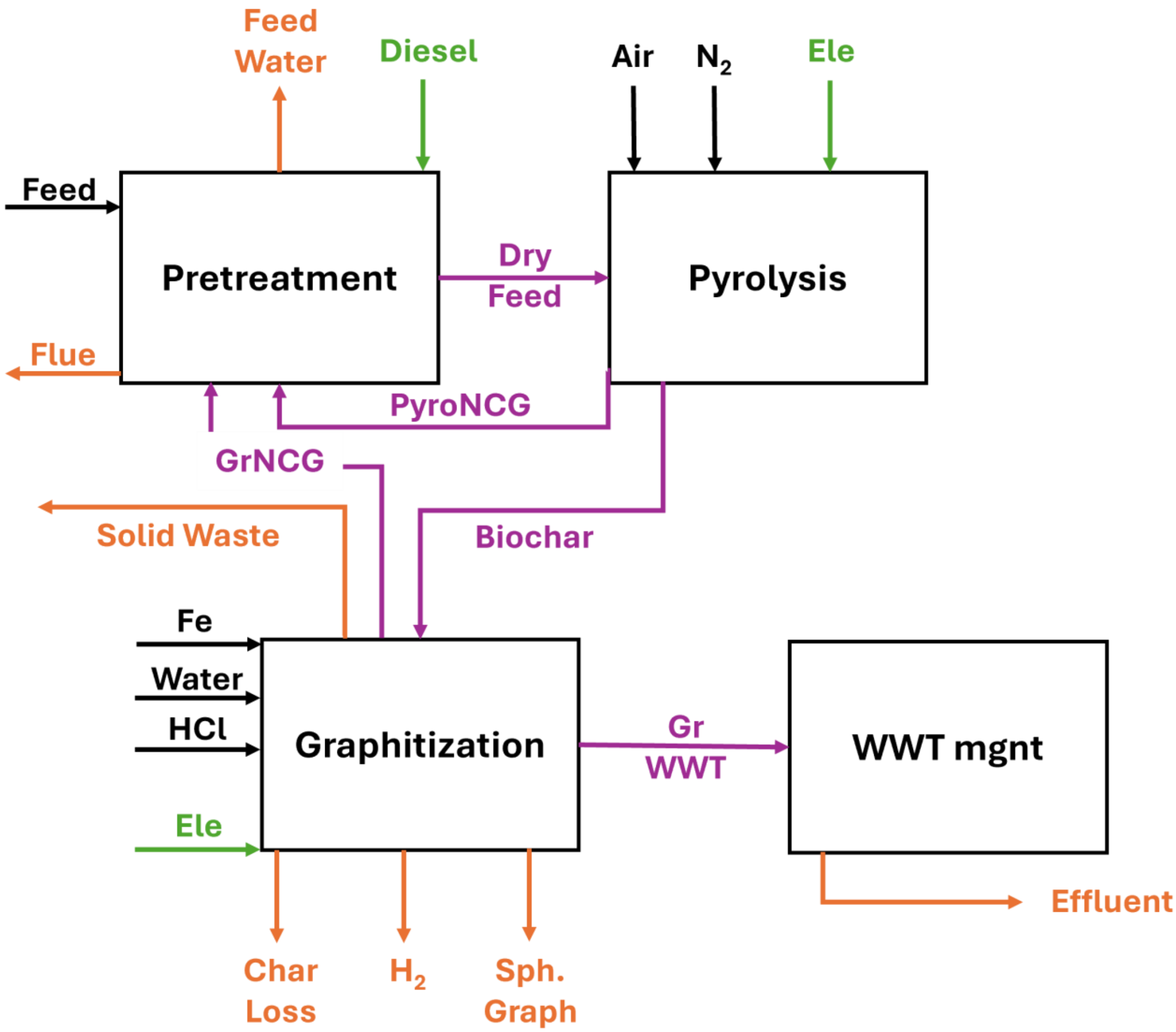

Figure S 1 Process flow chart for the Biochar pathway – BC1.

Feed: feedstock. Ele: electricity. Sph. Graph: spherical graphite. PyroNCG: non-condensable gases from pyrolysis. GrNCG: non-condensable gases from graphitization. GrWWT: wastewater from graphite production. WWT mgnt: wastewater management.

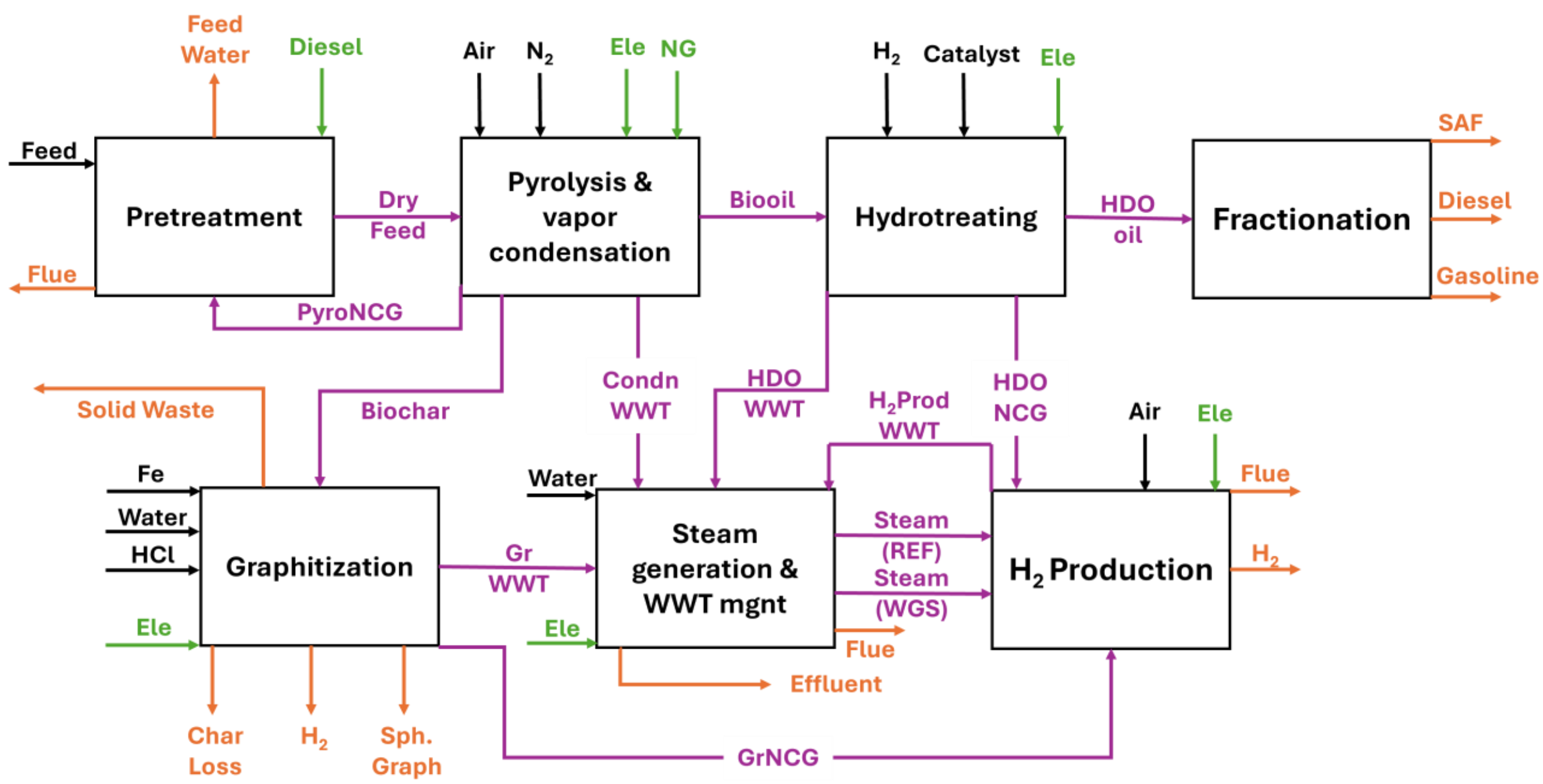

Figure S 2 Process flow chart for the Biochar pathway-BC2.

Feed: feedstock. Ele: electricity. Sph. Graph: spherical graphite. PyroNCG: non-condensable gases from pyrolysis. CondnWWT: wastewater from condensation. HDOoil: oil from hydrotreating. HDONCG: non-condensable gases from hydrotreating. HDOWWT: wastewater from hydrotreating. GrNCG: non-condensable gases from graphitization. GrWWT: wastewater from graphite production. WWT mgnt: wastewater management. Steam (REF): steam for reforming. Steam (WGS): steam for water-gas-shift.

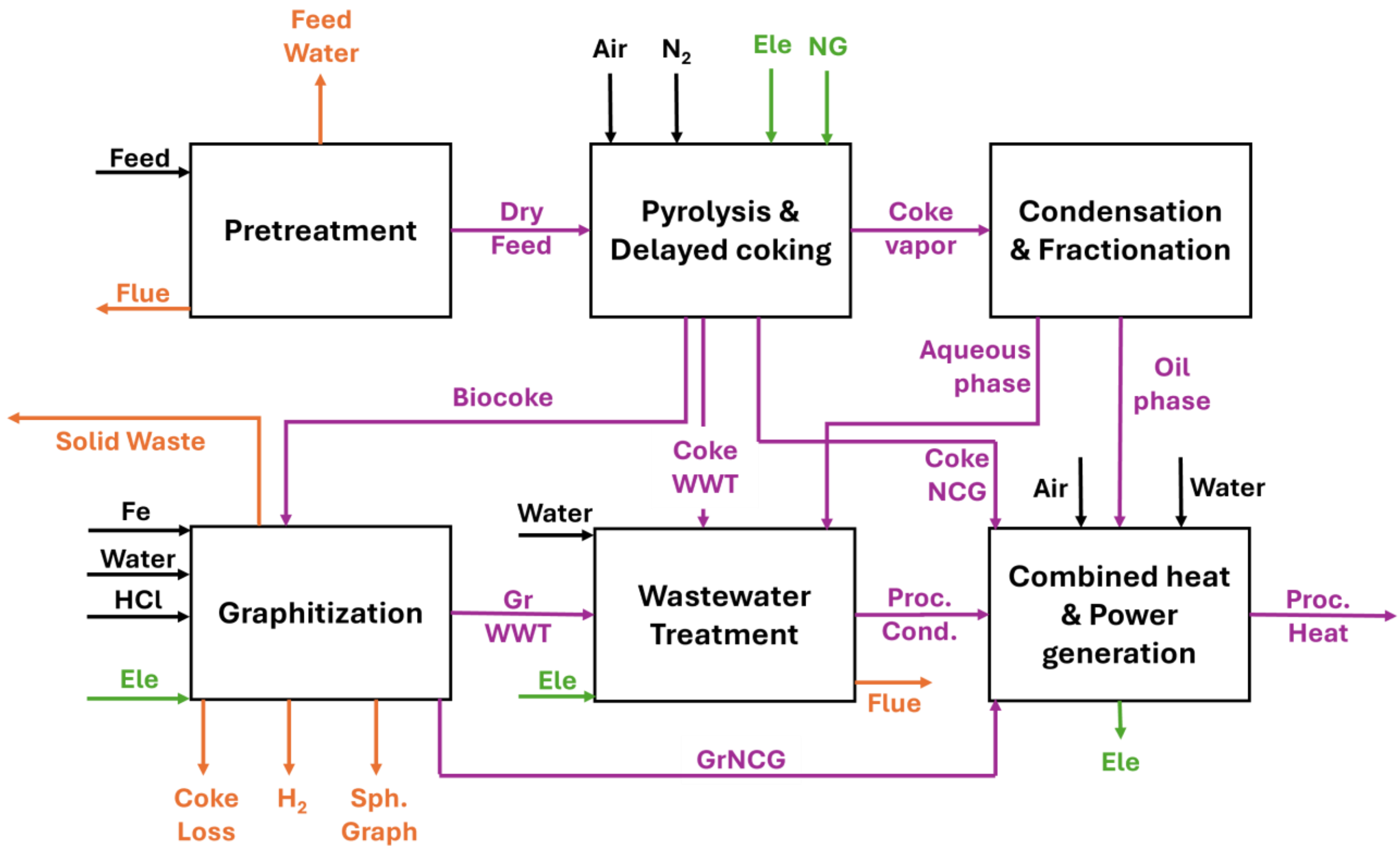

Figure S 3 Process flow chart for the Biocoke pathway – BO1.

Feed: feedstock. Ele: electricity. Sph. Graph: spherical graphite. CokeNCG: non-condensable gases from coking. CokeWWT: wastewater from coking. GrNCG: non-condensable gases from graphitization. GrWWT: wastewater from graphite production. WWT mgnt: wastewater management. Proc. Heat: process heat. Proc. Cond.: process condensates

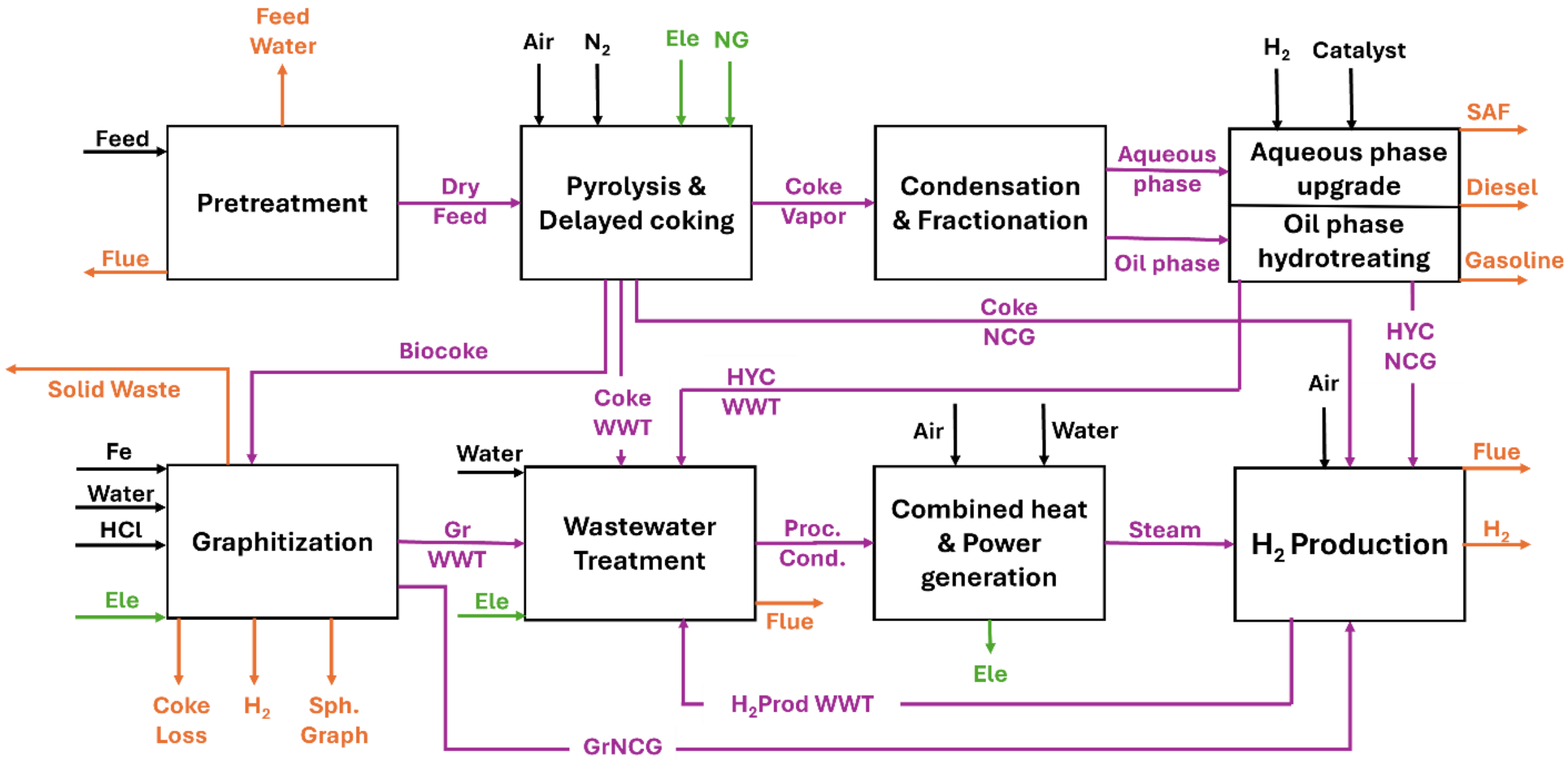

Figure S 4 Process flow chart for the Biocoke pathway – BO2

Hydrocarbon production consists of aqueous phase upgrading and oil phase hydrotreating. HYC NCG: non-condensable gases from hydrocarbon production. Feed: feedstock. Ele: electricity. Sph. Graph: spherical graphite. CokeNCG: non-condensable gases from coking. CokeWWT: wastewater from coking. GrNCG: non-condensable gases from graphitization. GrWWT: wastewater from graphite production. WWT mgnt: wastewater management. Proc. Heat: process heat. Proc. Cond.: process condensates

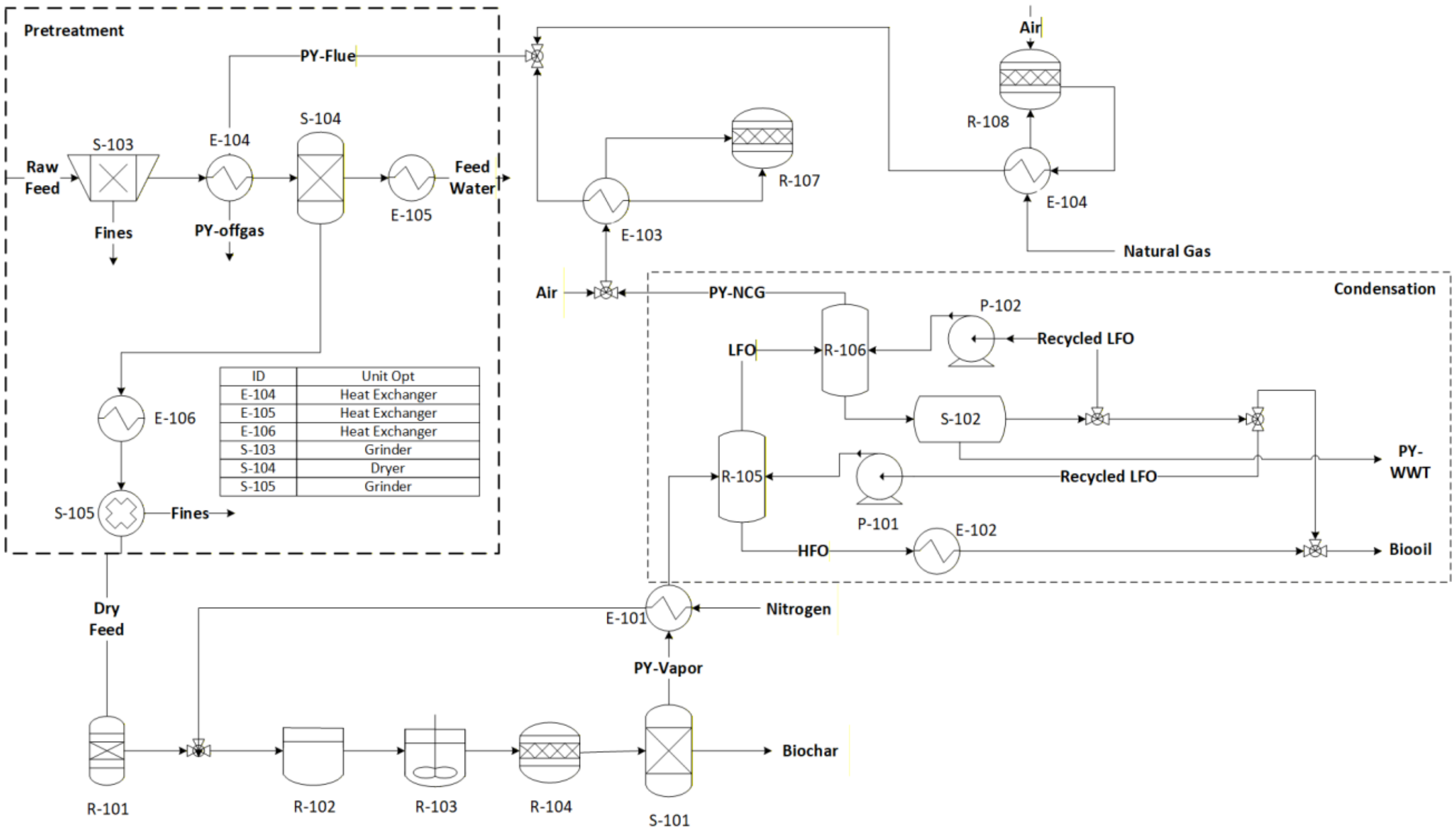

ID	Unit Opt
E-104	Heat Exchanger
E-105	Heat Exchanger
E-106	Heat Exchanger
S-103	Grinder
S-104	Dryer
S-105	Grinder

ID	Unit Opt
E-101	Heat Exchanger
E-102	Heat Exchanger
E-103	Heat Exchanger
E-104	Heat Exchanger
P-101	Pump
P-102	Pump
R-101	Decomposer
R-102	Pyrolysis reactor 1

ID	Unit Opt
R-103	Pyrolysis reactor 2
R-104	Final conversion
R-105	Heavy fraction condenser
R-106	Light fraction condenser
R-107	NCG combustor
R-108	Natural Gas combustor
S-101	Biochar, Vapor separator
S-102	Decanter

Figure S 5 Detailed process flow chart for unit operations in biochar production modeled in Aspen, including pretreatment, slow pyrolysis, vapor condensation

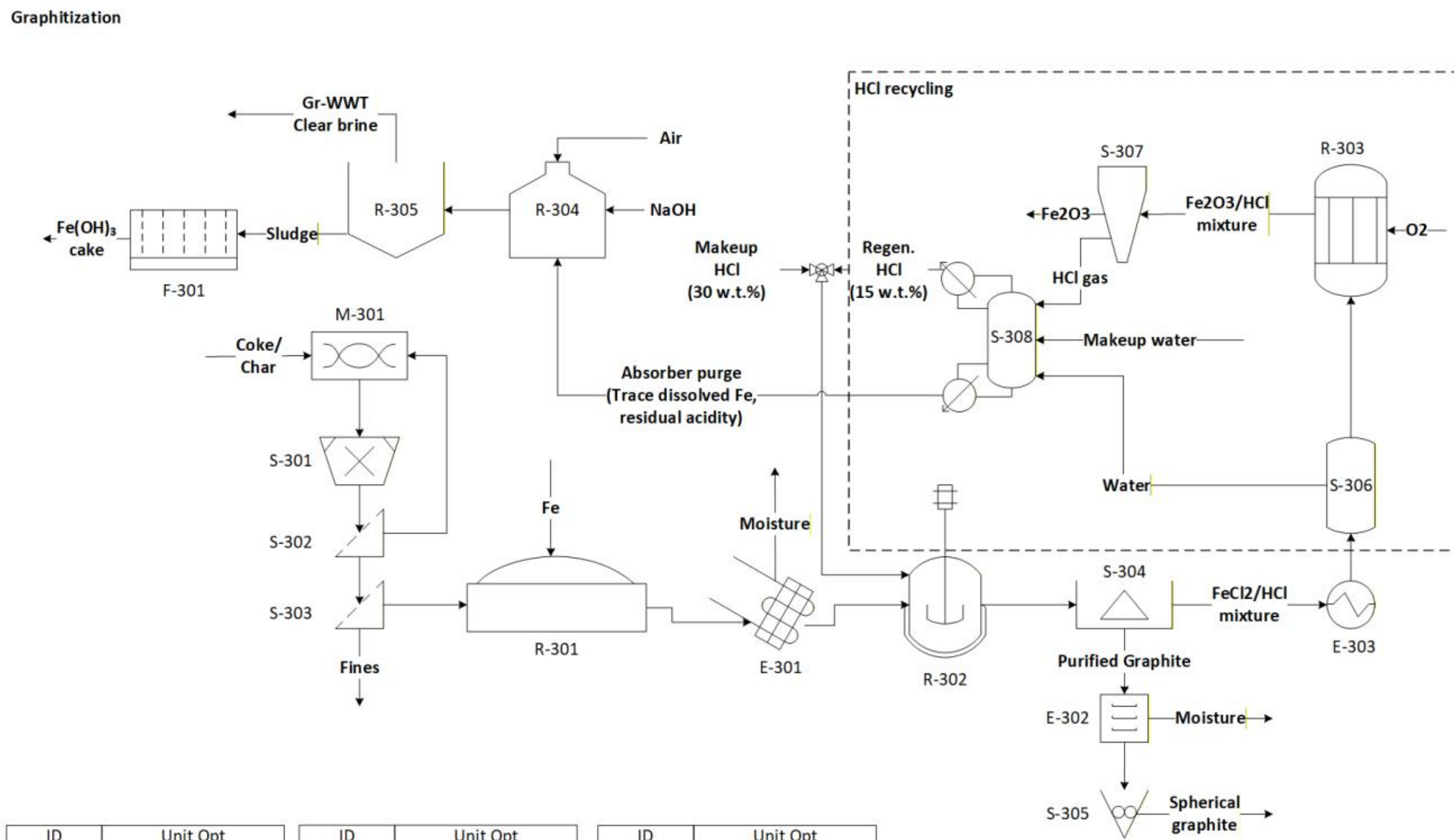

ID	Unit Opt
E-301	Rotary Cooler
E-302	Dryer
E-303	Cooler
F-301	Filter Press
M-301	Blender
R-301	Graphitization Furnace
R-302	Washer
R-303	Spray roaster

ID	Unit Opt
R-304	Neutralization Tank
R-305	Clarifier
S-301	Milling
S-302	Screening
S-303	Screening
S-304	Centrifigue
S-305	Shaping Mill
S-306	Preconcentrator

ID	Unit Opt
S-307	Cyclone
S-308	Absorber

Figure S 6 Detailed process flow chart for unit operations in graphite production modeled in Aspen, including Fe-catalytic graphitization, acid recycling, and post-treatment, based on previous work[7]

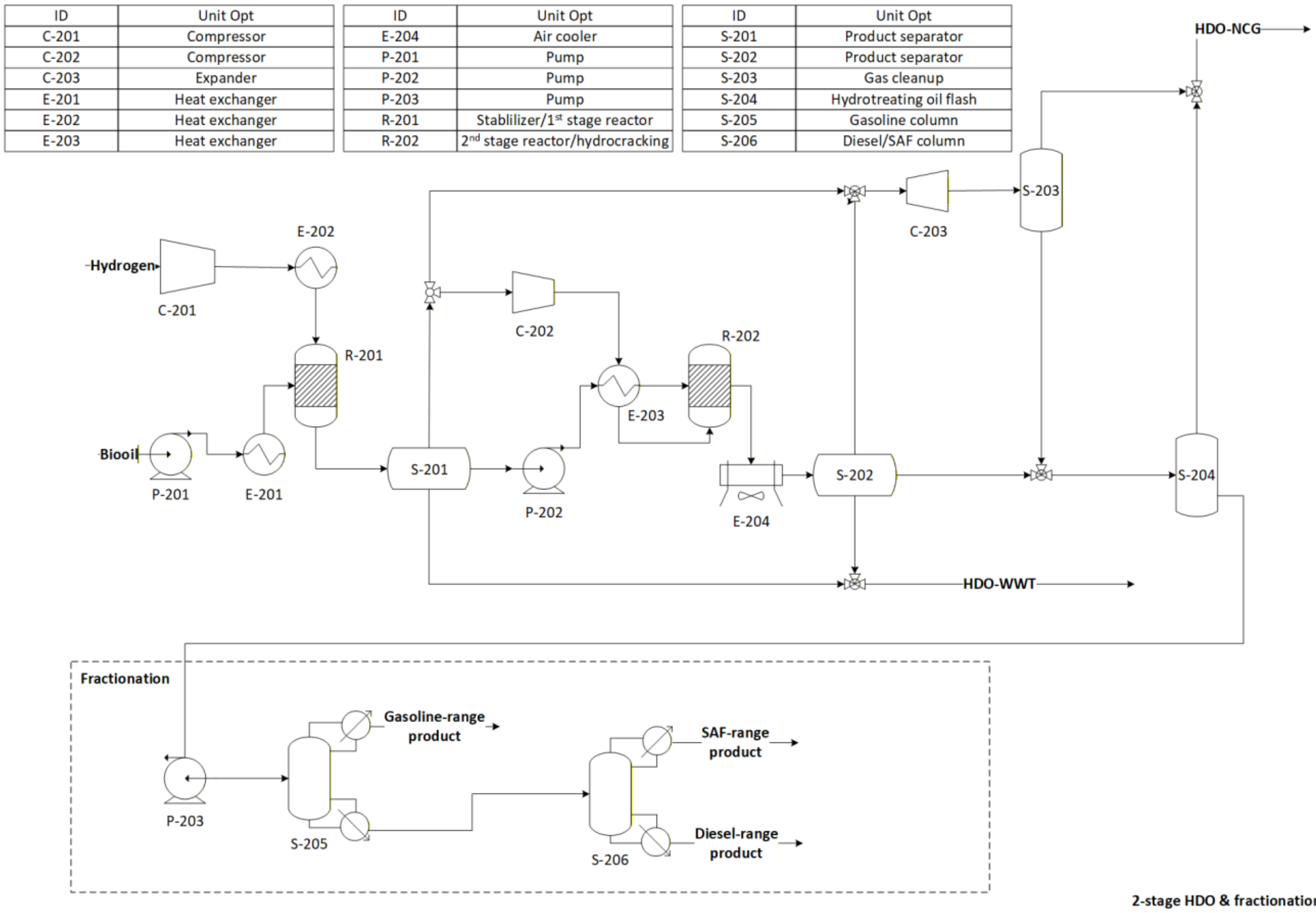

ID	Unit Opt	ID	Unit Opt	ID	Unit Opt
C-201	Compressor	E-204	Air cooler	S-201	Product separator
C-202	Compressor	P-201	Pump	S-202	Product separator
C-203	Expander	P-202	Pump	S-203	Gas cleanup
E-201	Heat exchanger	P-203	Pump	S-204	Hydrotreating oil flash
E-202	Heat exchanger	R-201	Stablilizer/1st stage reactor	S-205	Gasoline column
E-203	Heat exchanger	R-202	2nd stage reactor/hydrocracking	S-206	Diesel/SAF column

Figure S 7 Detailed process flow chart for unit operations in hydrocarbon production modeled in Aspen, including two-stage hydrodeoxygenation (HDO) and fractionation

H2 production

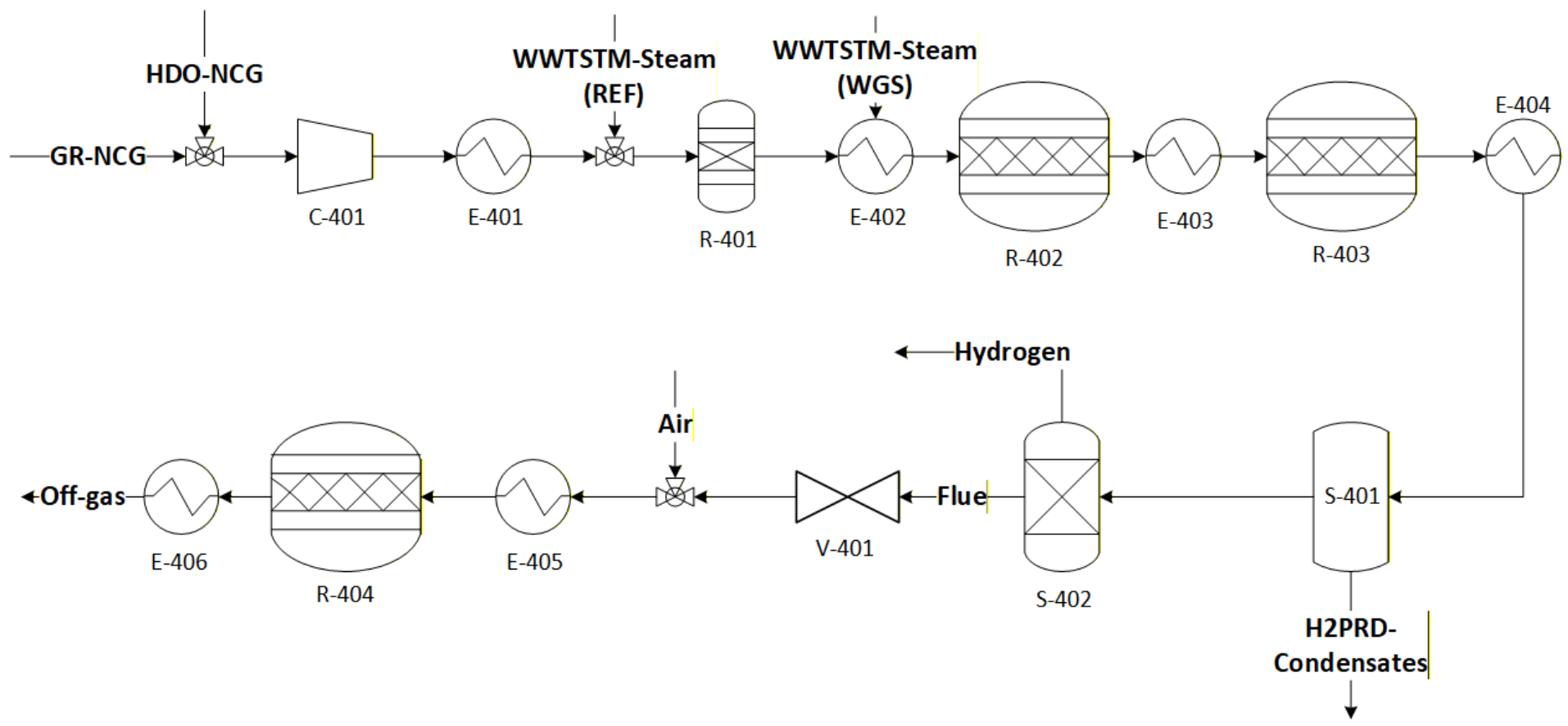

ID	Unit Opt
C-401	NCG compressor
E-401	Heat exchanger
E-402	Heat exchanger
E-403	Heat exchanger
E-404	Heat exchanger
E-405	Heat exchanger
E-406	Cooler

ID	Unit Opt
R-401	Reformer
R-402	High-temperature water-gas-shift (HT-WGS)
R-403	Low-temperature water-gas-shift (LT-WGS)
R-404	PSA off-gas combustor
S-401	WGS product flash
S-402	Pressure Swing Adsorption (PSA)
V-401	Valve

Figure S 8 Detailed process flow chart for unit operations in H_2 production modeled in Aspen, including reforming, water-gas-shift (WGS), and pressure swing adsorption (PSA)

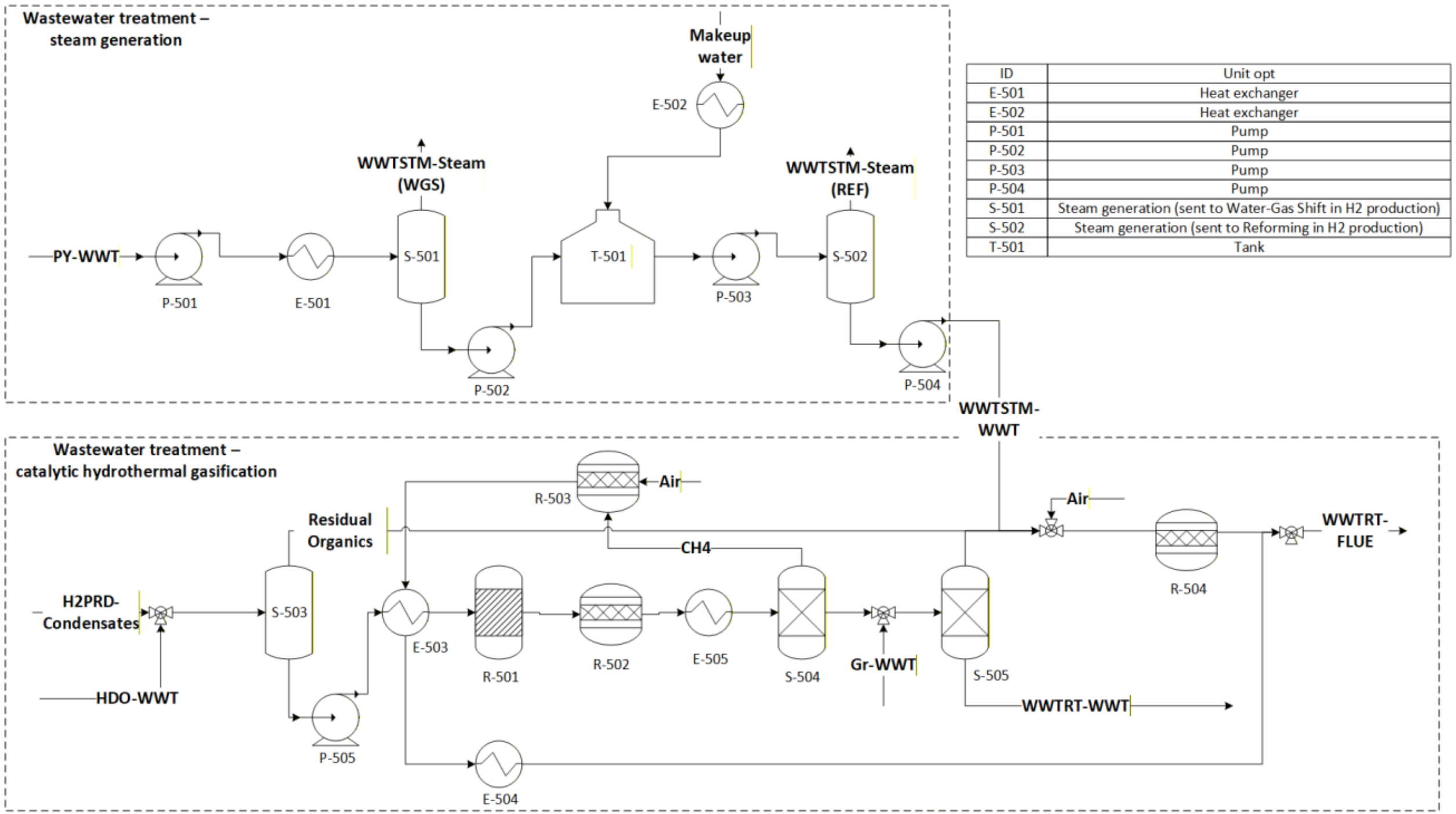

ID	Unit opt
E-501	Heat exchanger
E-502	Heat exchanger
P-501	Pump
P-502	Pump
P-503	Pump
P-504	Pump
S-501	Steam generation (sent to Water-Gas Shift in H2 production)
S-502	Steam generation (sent to Reforming in H2 production)
T-501	Tank

ID	Unit opt
E-503	Heat exchanger
E-504	Heat exchanger
E-505	Heat exchanger
P-505	Pump
R-501	Catalytic hydrothermal gasification (CHG)
R-502	Carbon Monoxide (CO) cleanup

ID	Unit opt
R-503	Combustor (CH4 combustion)
R-504	Regenerative Thermal Oxidizer (RTO)
S-503	Knockout drum
S-504	Methane (CH4) separation
S-505	Reverse Osmosis (RO)

Figure S 9 Detailed process flow chart for unit operations in Wastewater treatment modeled in Aspen, including steam generation and catalytic hydrothermal gasification (CHG)

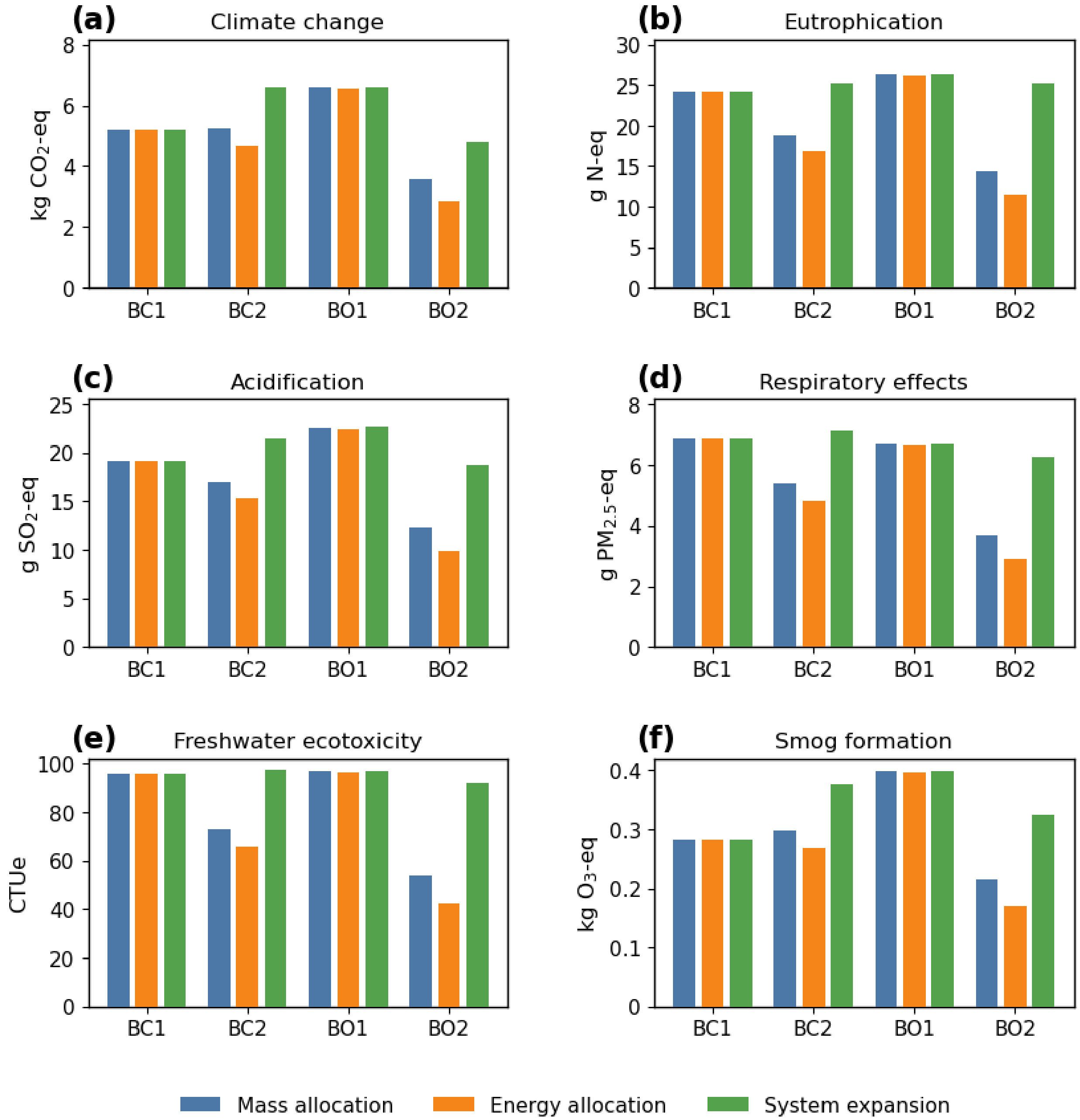

Figure S 10 LCA results for 1 kg graphite across various impact categories and allocation methods for four bio-graphite pathways.

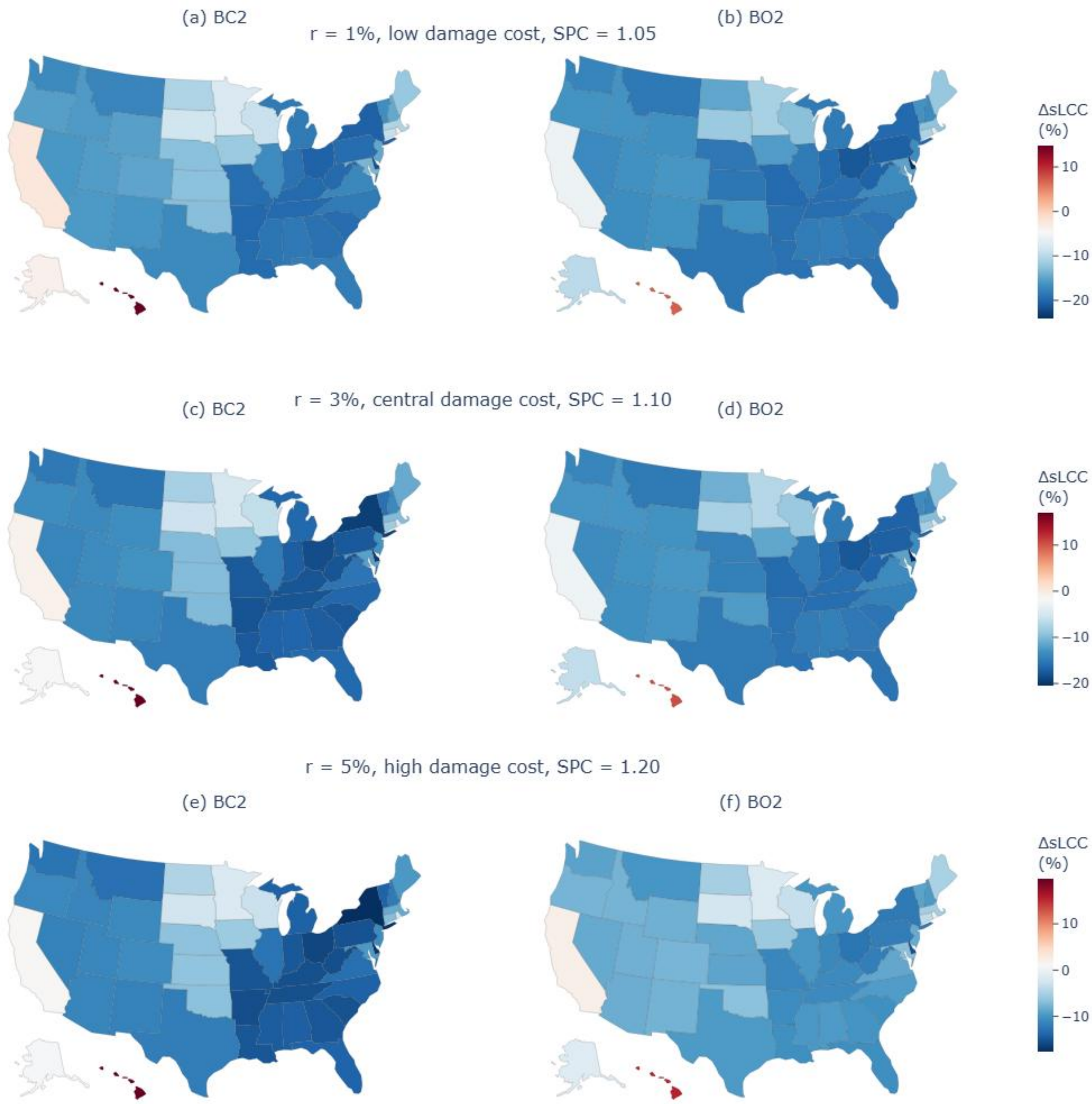

Figure S 11 Average sLCC change compared to market synthetic across 6 impact categories and sLCC assumptions

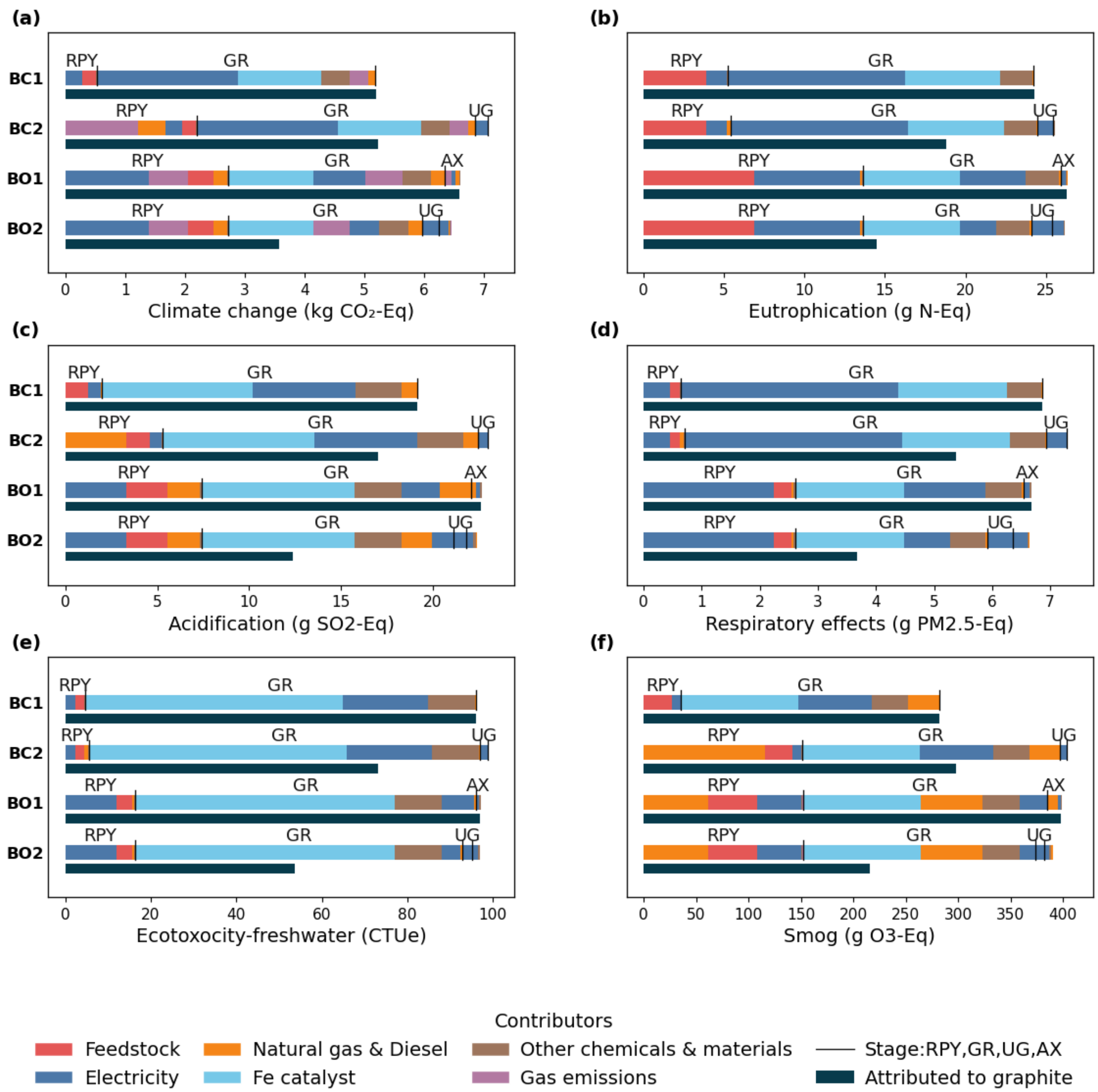

Figure S 12 LCA result breakdown for 1 kg of bio-graphite

Breakdown is shown by energy, materials from different unit process stages, including RPY (residue collection and pyrolysis), GR (Graphitization), UG (Non-condensable gas, pyrolysis vapor upgrade), AX (other auxiliary processes, e.g., cooling, wastewater treatment). The stacked bar charts display the results for the entire processes, while the impacts allocated to graphite by mass are represented by bars labeled “attributed to graphite”.

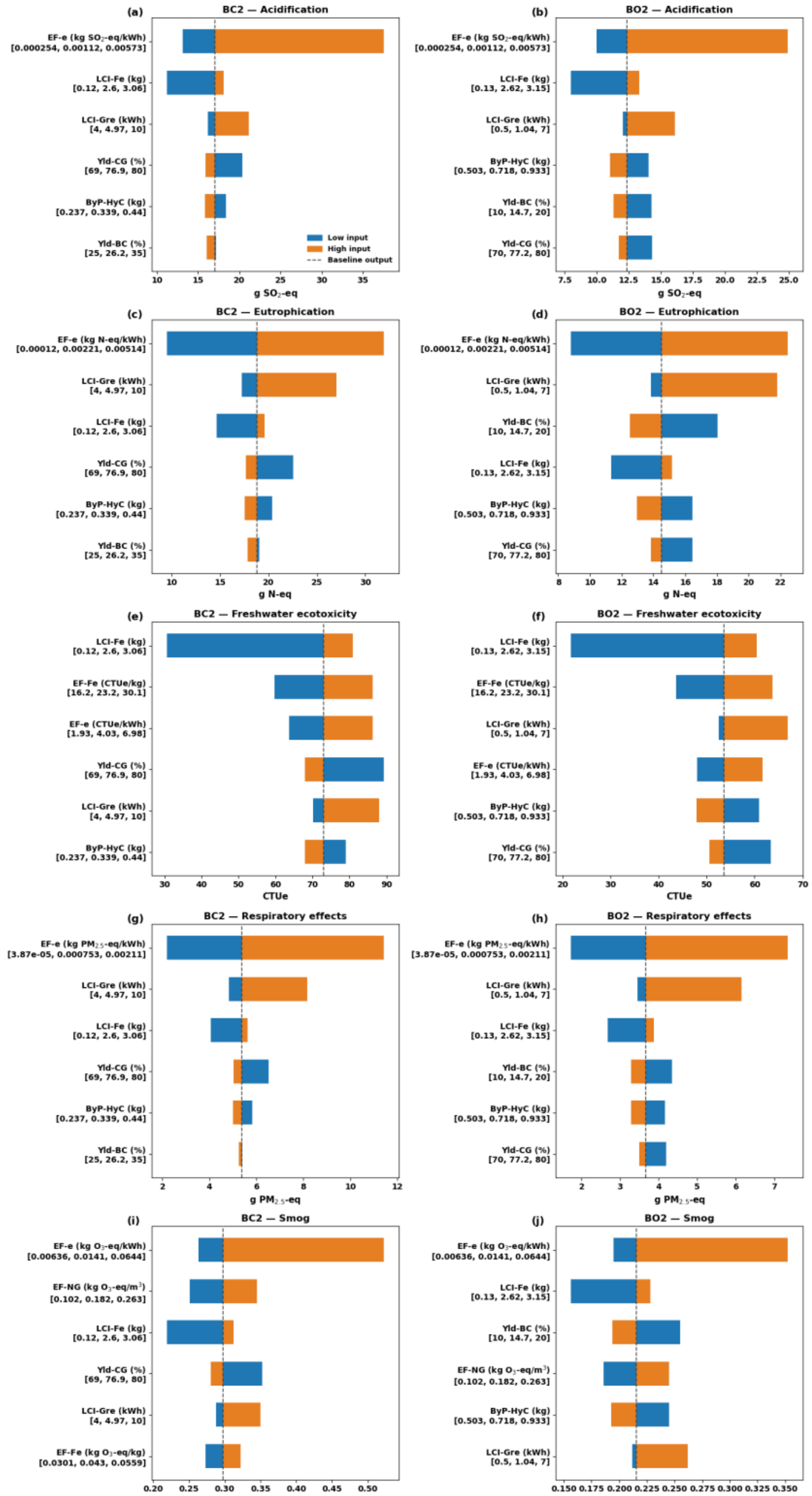

Figure S 13 BC2 and BO2 sensitivity on various environmental impacts

LCI variables: LCI-Fe: Fe catalyst consumption (kg/kg graphite); LCI-Gre: graphitization electricity consumption (kWh/kg graphite); ByP-HyC: byproduct-hydrocarbon amount (kg/kg graphite). Price variables: Pc-e: price of electricity (₵/kWh); Pc-Fe: price of Fe catalyst ($/kg of

Fe catalyst); Pc-feed: price of feedstock (₵/kg of feedstock). Emission factors (EF) units are shown according to the impact categories: EF-e: electricity environmental footprint; EF-Fe: Fe catalyst environmental footprint; EF-NG: natural gas environmental footprint; EF-feed: feedstock environmental footprint. Yield variables: Yld-CG: the yield from converting biochar/biocoke to graphite (%); Yld-BC: the yield from converting biomass to biochar/biocoke.

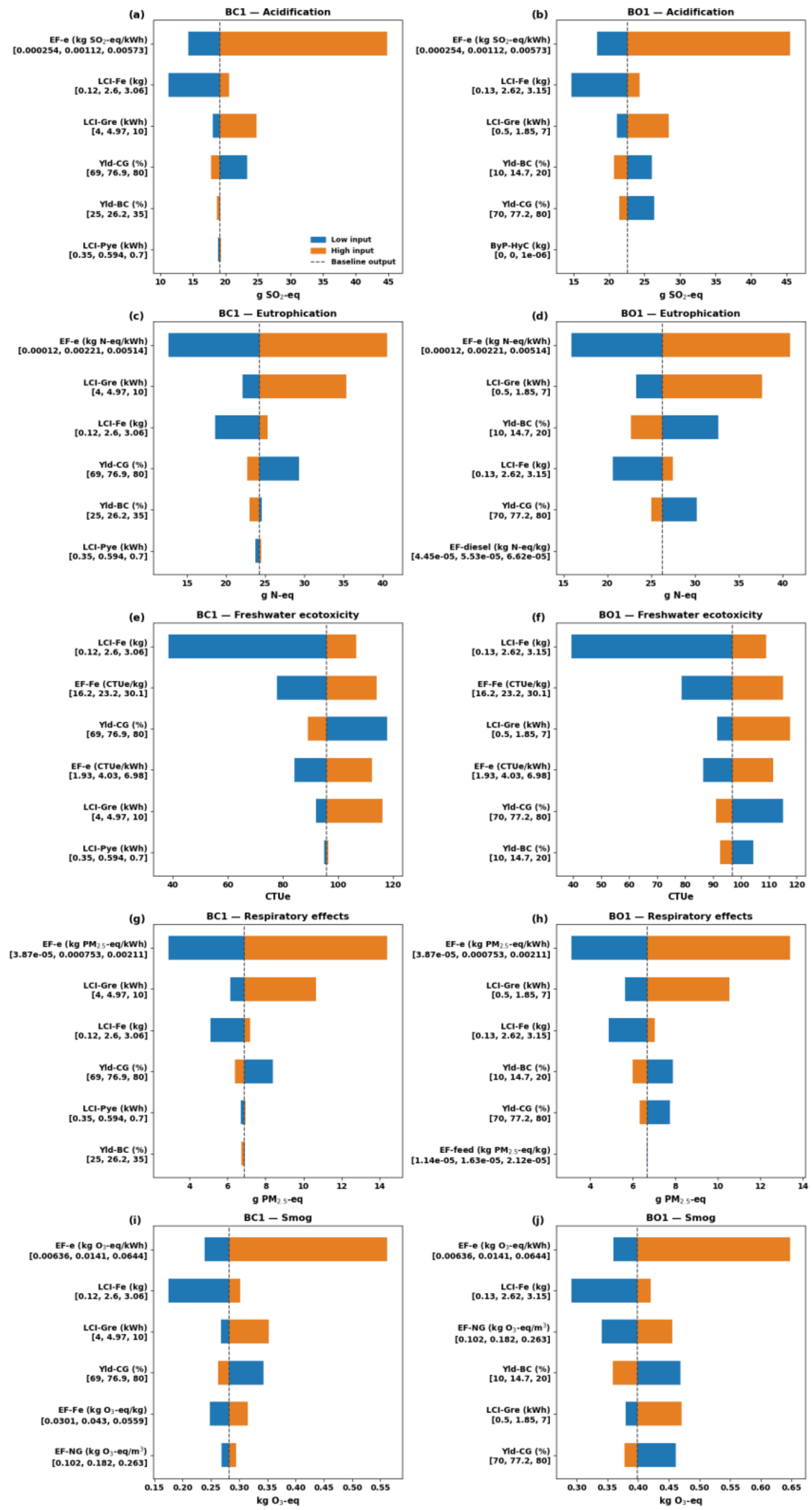

Figure S 14 BC1 and BO1 sensitivity on various environmental impacts

LCI variables: LCI-Fe: Fe catalyst consumption (kg/kg graphite); LCI-Gre: graphitization electricity consumption (kWh/kg graphite); ByP-HyC: byproduct-hydrocarbon amount (kg/kg

graphite). Price variables: Pc-e: price of electricity (₵/kWh); Pc-Fe: price of Fe catalyst ($/kg of Fe catalyst); Pc-feed: price of feedstock (₵/kg of feedstock). Emission factors (EF) units are shown according to the impact categories: EF-e: electricity environmental footprint; EF-Fe: Fe catalyst environmental footprint; EF-NG: natural gas environmental footprint; EF-feed: feedstock environmental footprint. Yield variables: Yld-CG: the yield from converting biochar/biocoke to graphite (%); Yld-BC: the yield from converting biomass to biochar/biocoke.

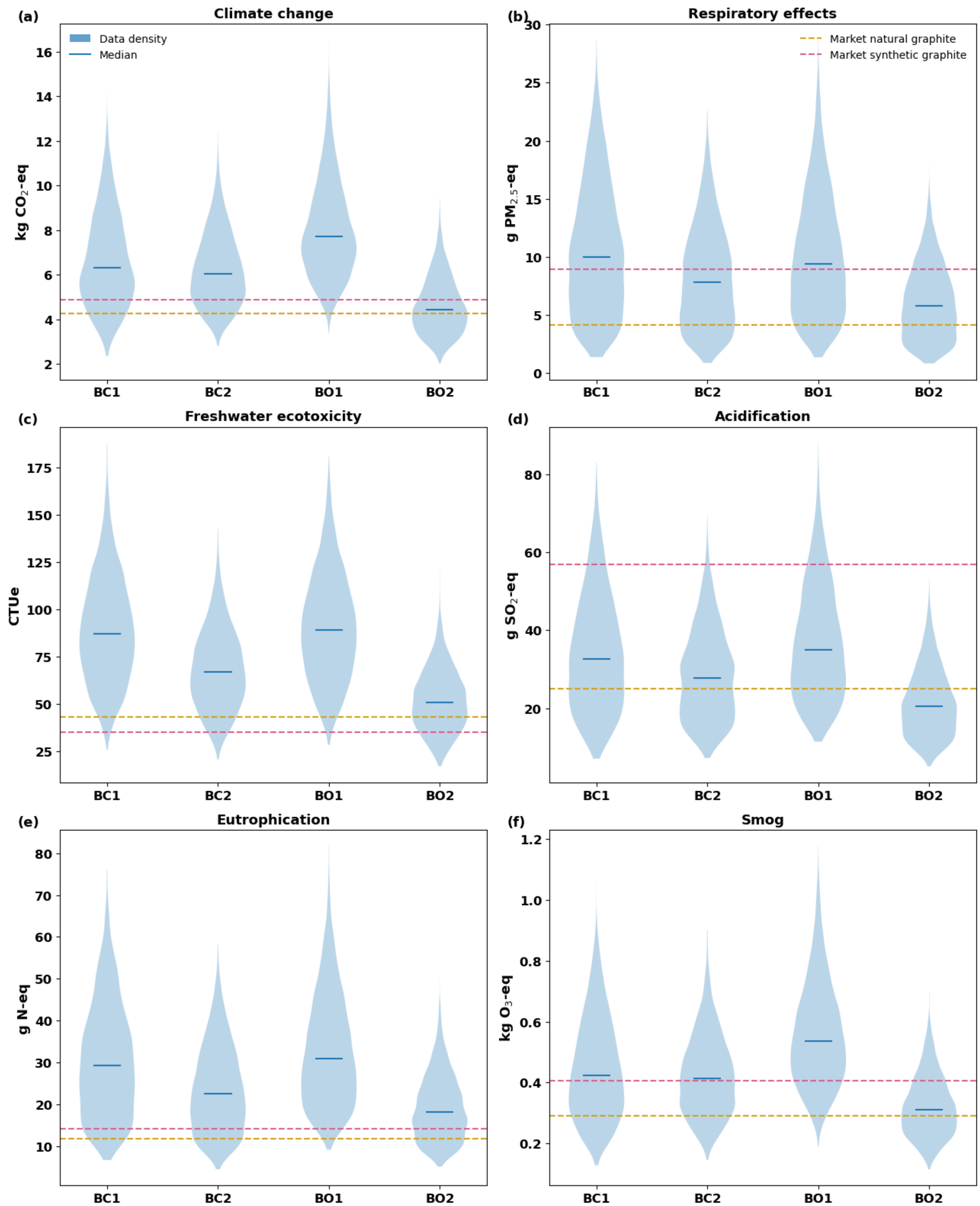

Figure S 15 Monte Carlo simulation results for multiple impact categories

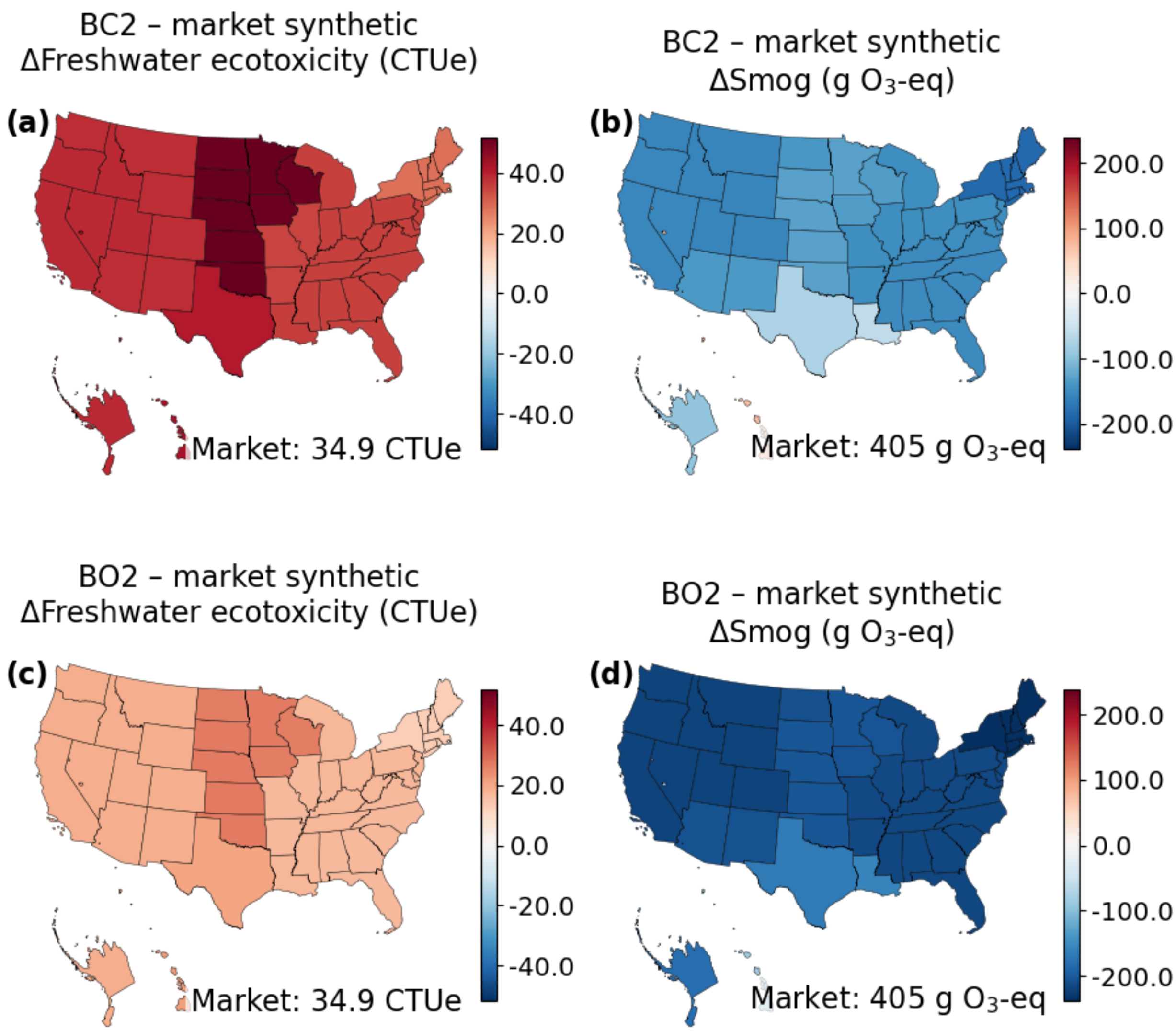

Figure S 16 Environmental impacts (from mass allocation, cradle-to-gate) for 1 kg of bio-graphite produced from BC2 and BO2 pathways and compared to the market synthetic graphite. Environmental Impact: Freshwater Ecotoxicity, Smog.

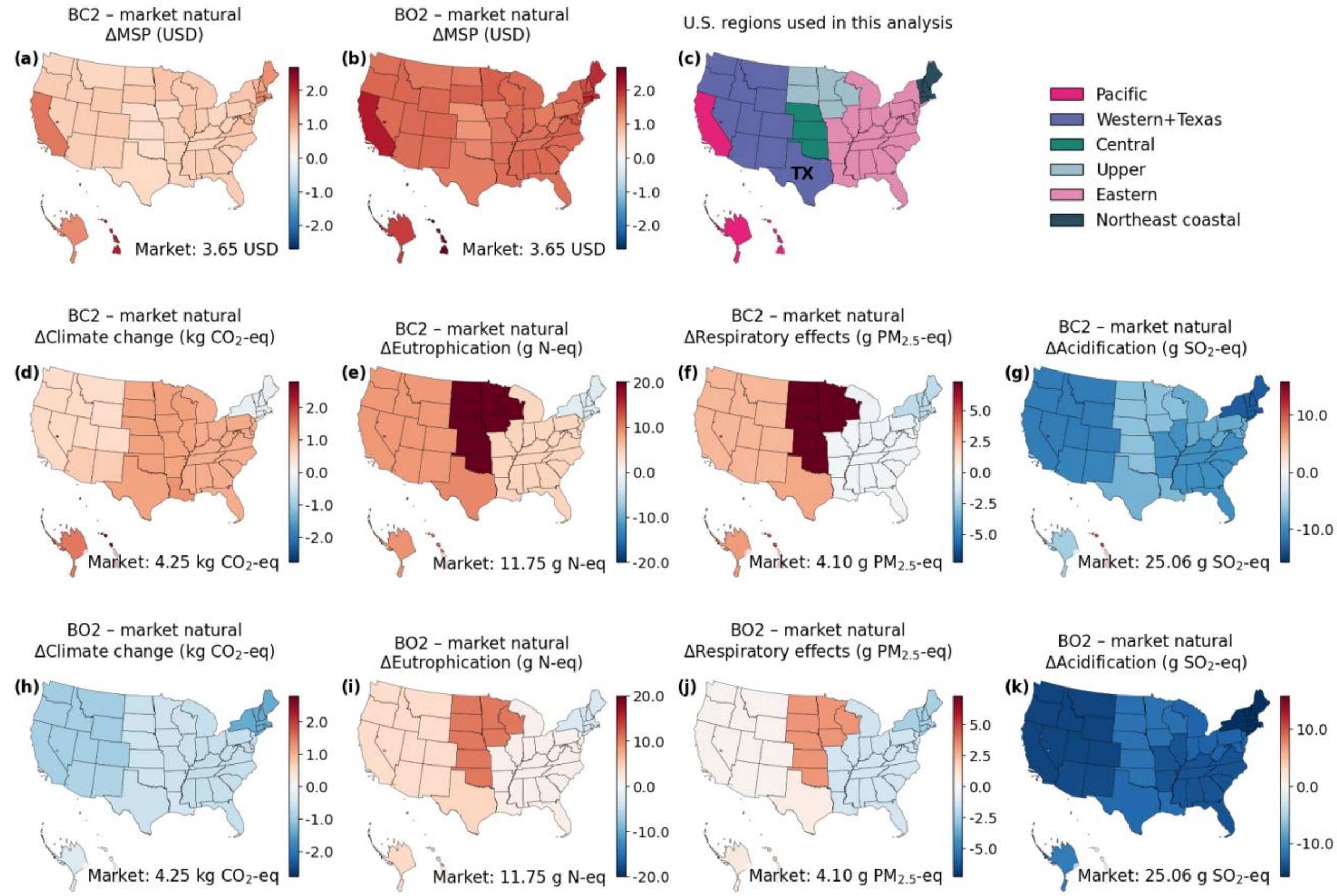

Figure S 17 MSP and environmental impacts (from mass allocation, cradle-to-gate) for 1 kg of bio-graphite produced from BC2 and BO2 pathways and compared to the average market natural graphite. Impact: MSP, climate change, eutrophication, respiratory effects, acidification

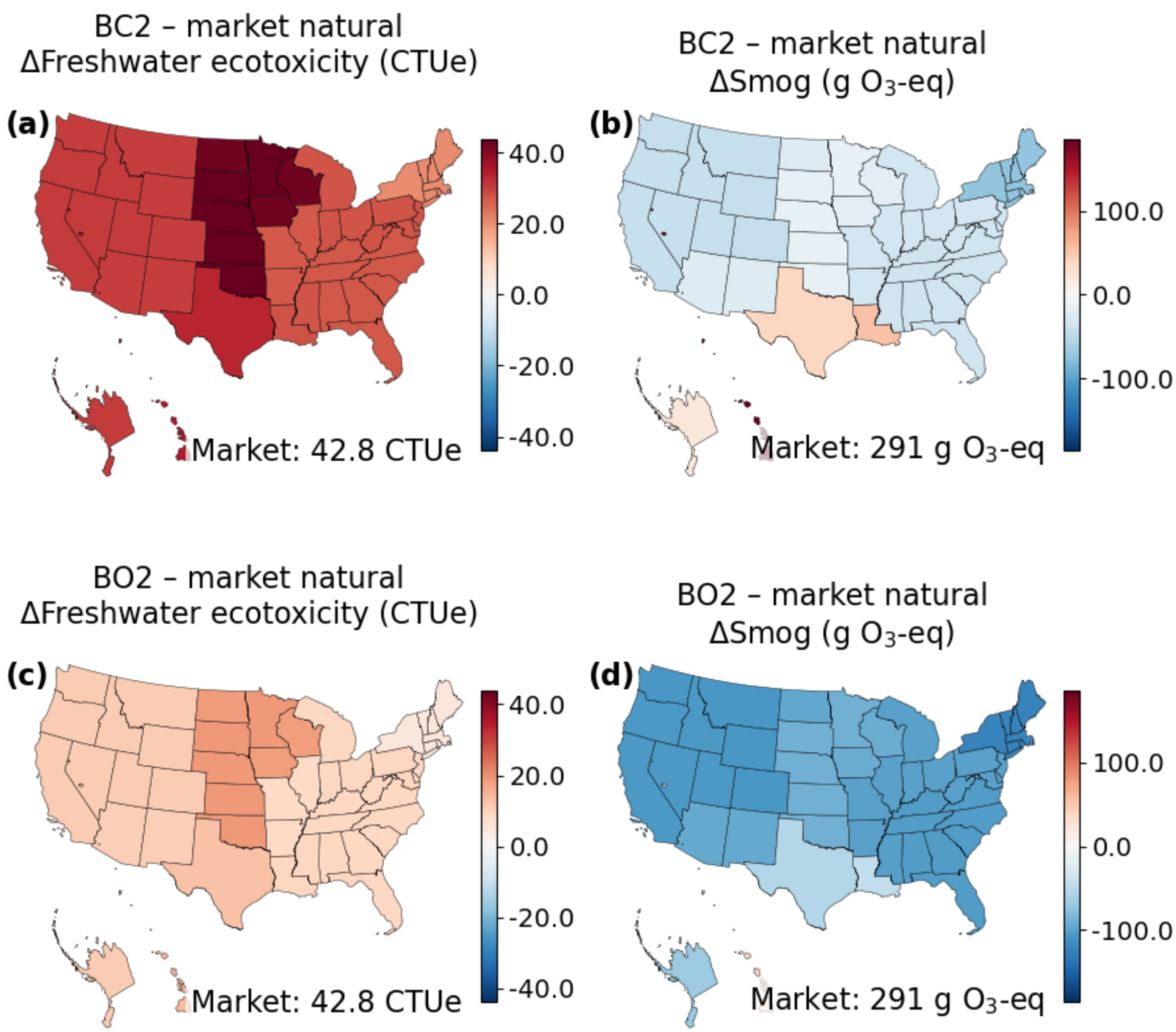

Figure S 18 Environmental impacts (from mass allocation, cradle-to-gate) for 1 kg of bio-graphite produced from BC2 and BO2 pathways and compared to the average market natural graphite. Impact: ecotoxicity, smog

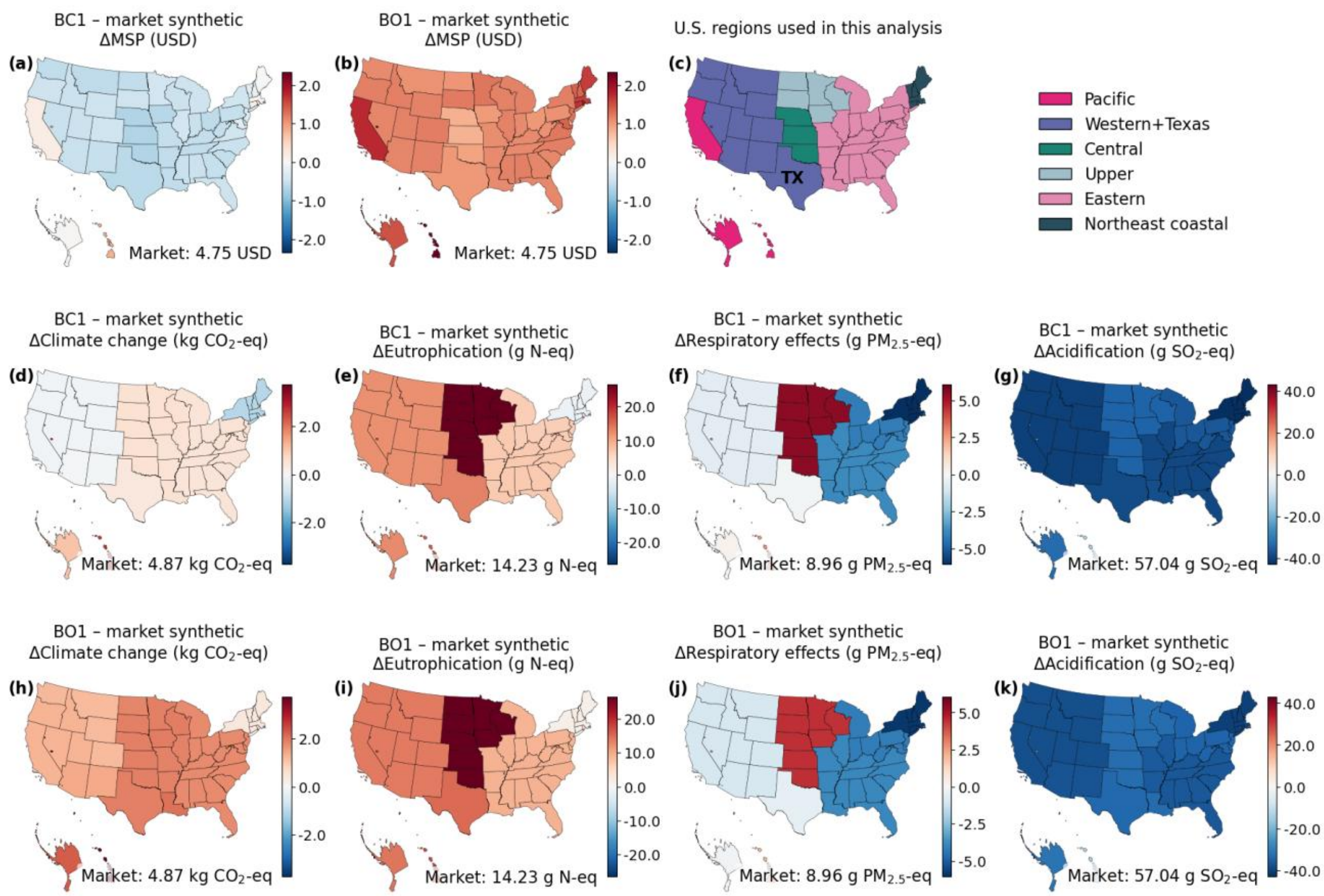

Figure S 19 MSP and environmental impacts (from mass allocation, cradle-to-gate) for 1 kg of bio-graphite produced from BC1 and BO1 pathways and compared to the market synthetic graphite. Impact: MSP, climate change, eutrophication, respiratory effects, acidification

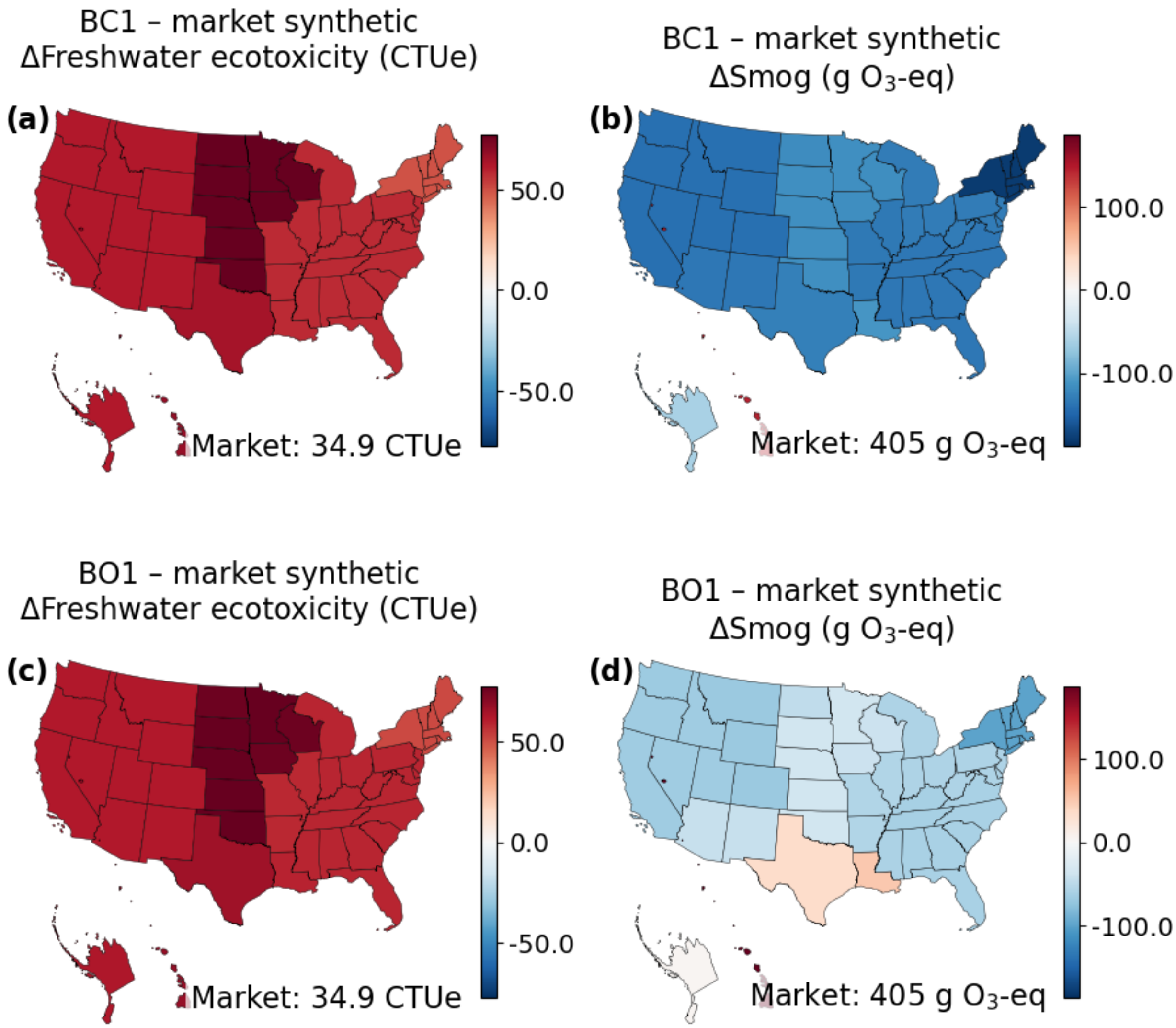

Figure S 20 Environmental impacts (from mass allocation, cradle-to-gate) for 1 kg of bio-graphite produced from BC1 and BO1 pathways and compared to the market synthetic graphite. Impact: freshwater ecotoxicity, smog

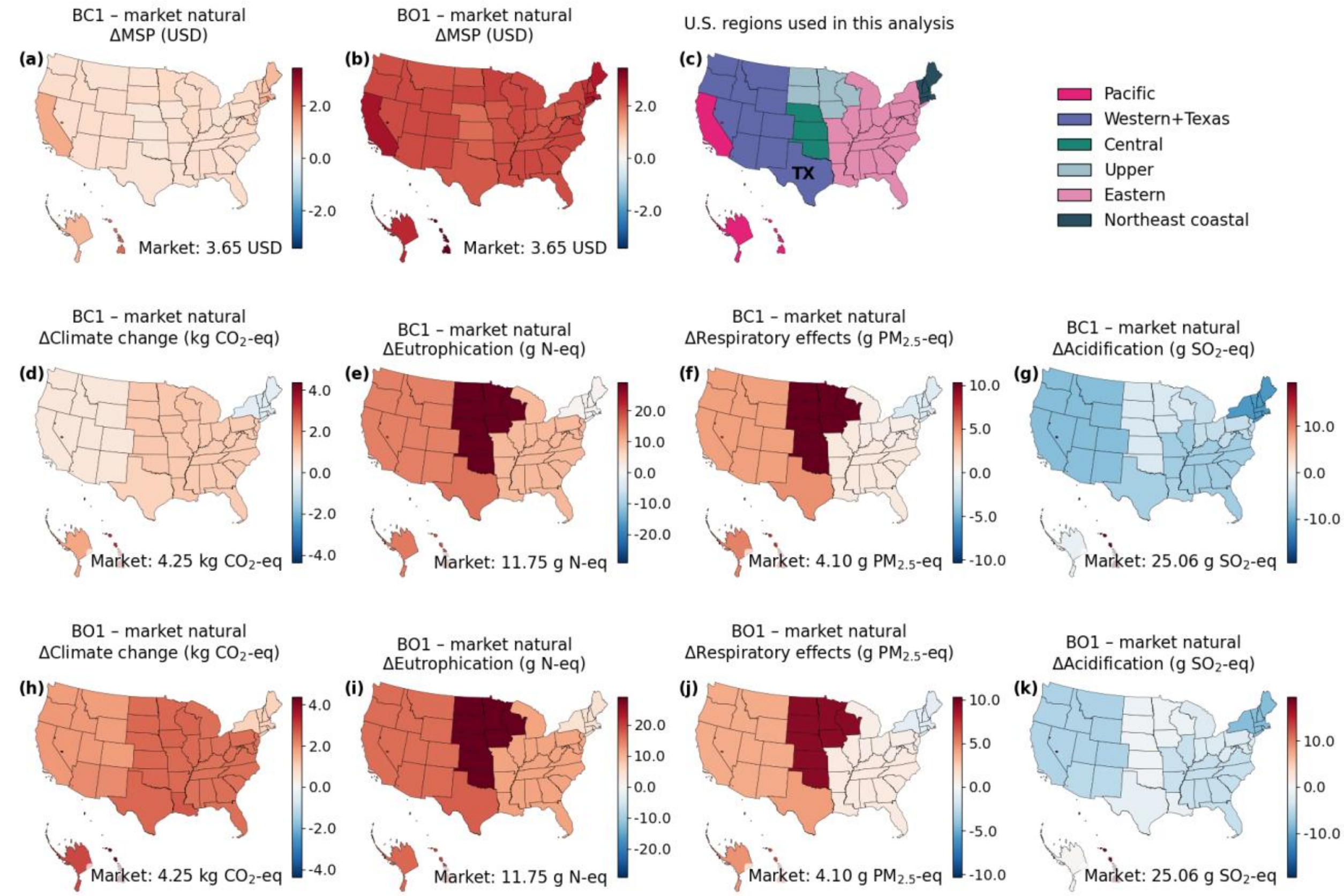

Figure S 21 MSP and environmental impacts (from mass allocation, cradle-to-gate) for 1 kg of bio-graphite produced from BC1 and BO1 pathways and compared to the market natural graphite. Impact: MSP, climate change, eutrophication, respiratory effects, acidification

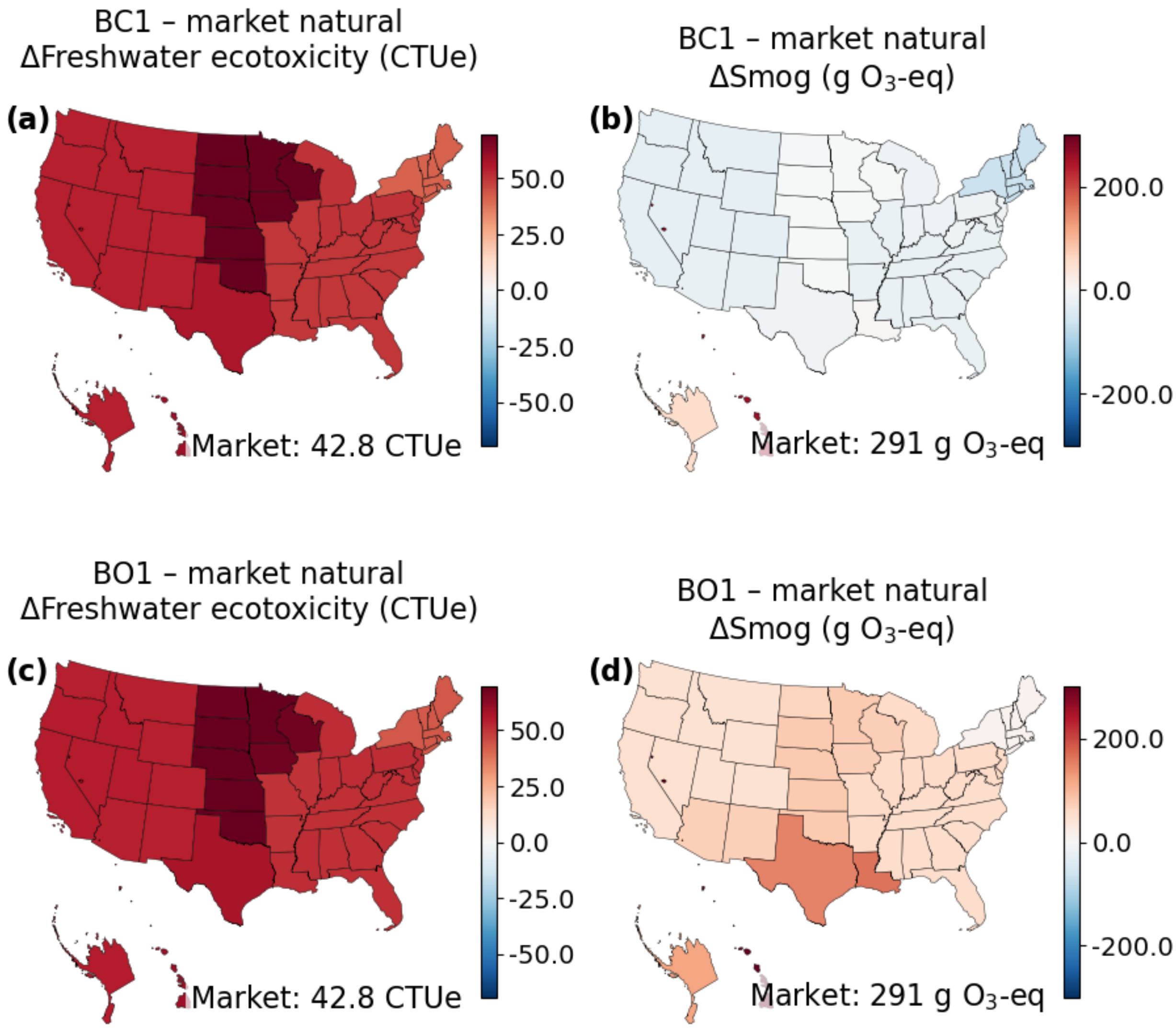

Figure S 22 Environmental impacts (from mass allocation, cradle-to-gate) for 1 kg of bio-graphite produced from BC1 and BO1 pathways and compared to the market natural graphite. Impact: freshwater ecotoxicity, smog

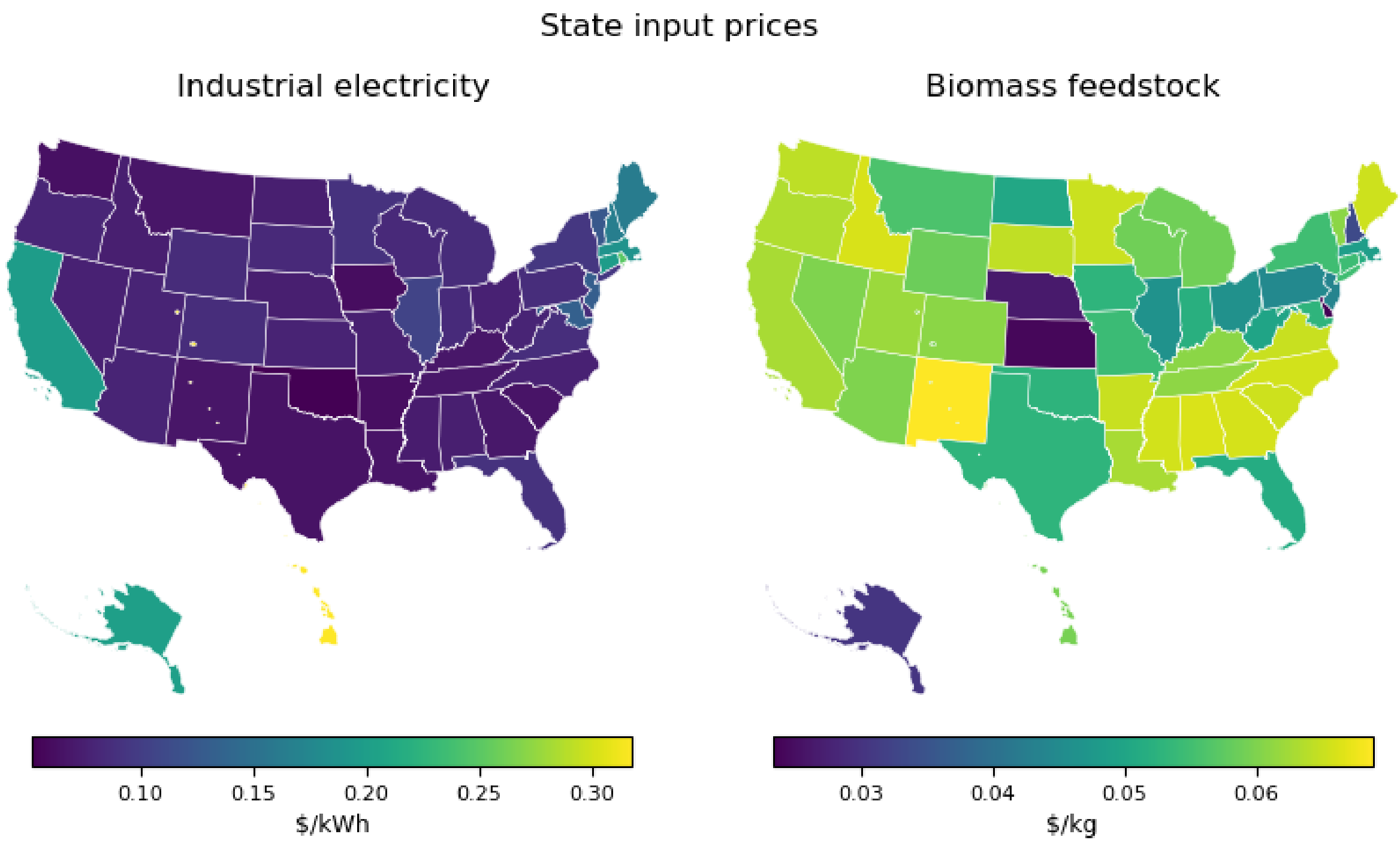

Figure S 23 Electricity and biomass feedstock prices across the U.S.

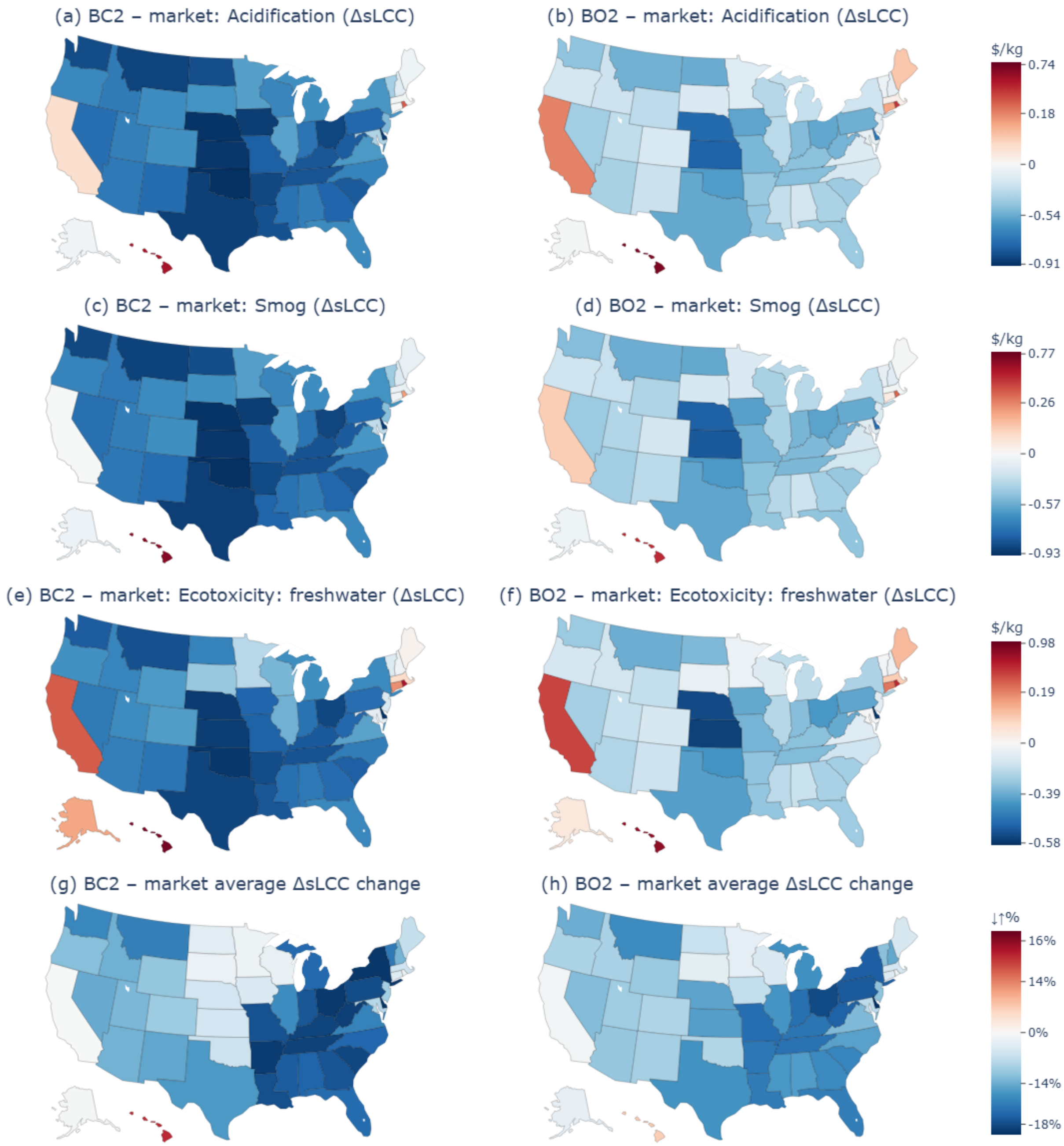

Figure S 24 Societal life cycle costing from bio-graphite and compared to sLCC from market synthetic graphite. (scenario: middle damage cost, 3% social rate)

(a-f): absolute difference between bio-graphite sLCC and market synthetic graphite sLCC for impact category: acidification, smog, ecotoxicity: freshwater. (g-h): average change of bio-graphite sLCC and market synthetic graphite sLCC (%) across all 6 impact categories: climate change, eutrophication, respiratory effects, acidification, smog, ecotoxicity: freshwater.

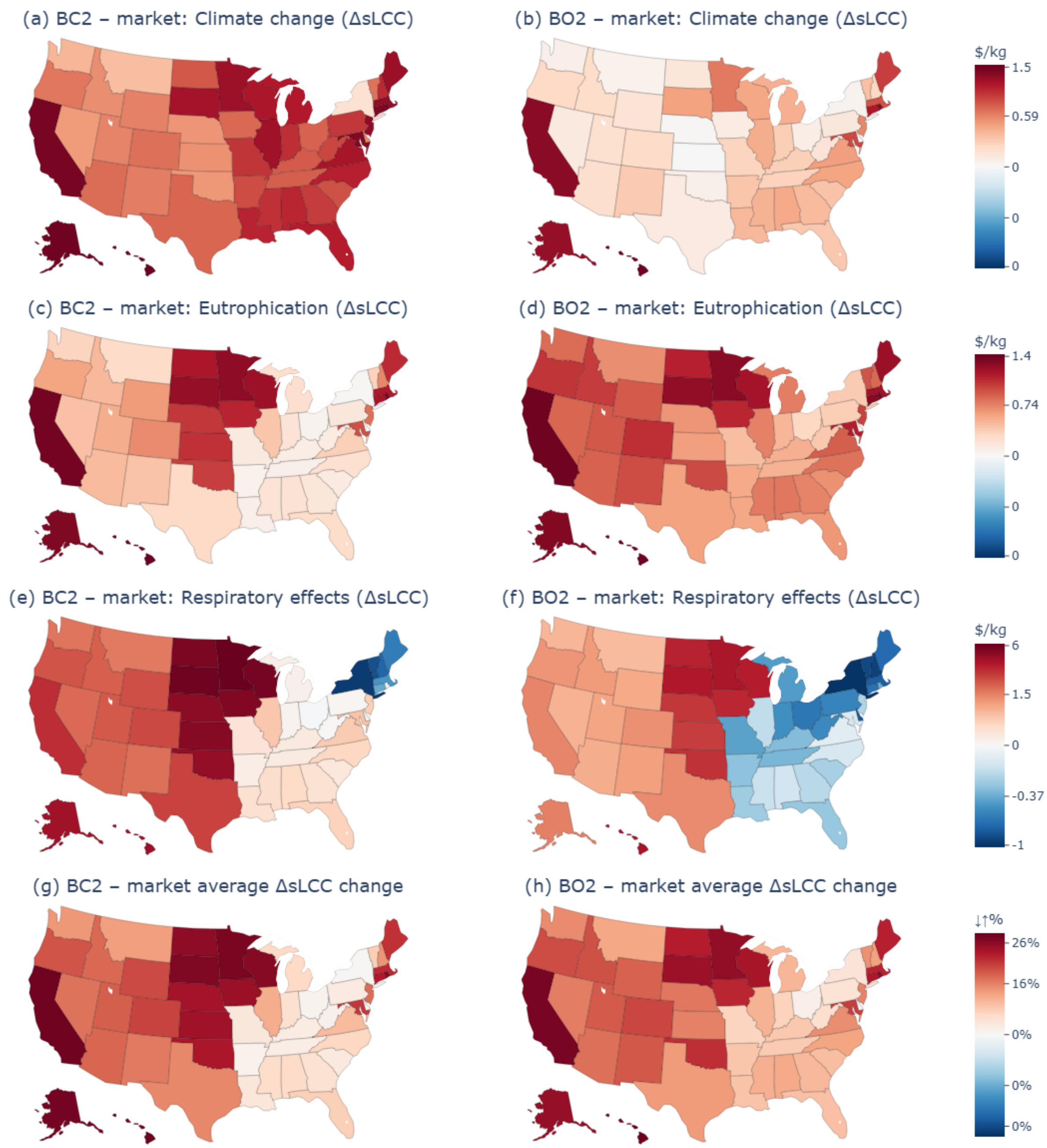

Figure S 25 Societal life cycle costing from bio-graphite and compared to sLCC from market natural graphite. (scenario: middle damage cost, 3% social rate)

(a-f): absolute difference between bio-graphite sLCC and market natural graphite sLCC for impact category: climate change, eutrophication, respiratory effects. (g-h): average change of bio-graphite sLCC and market natural graphite sLCC (%) across all 6 impact categories: climate change, eutrophication, respiratory effects, acidification, smog, freshwater ecotoxicity.

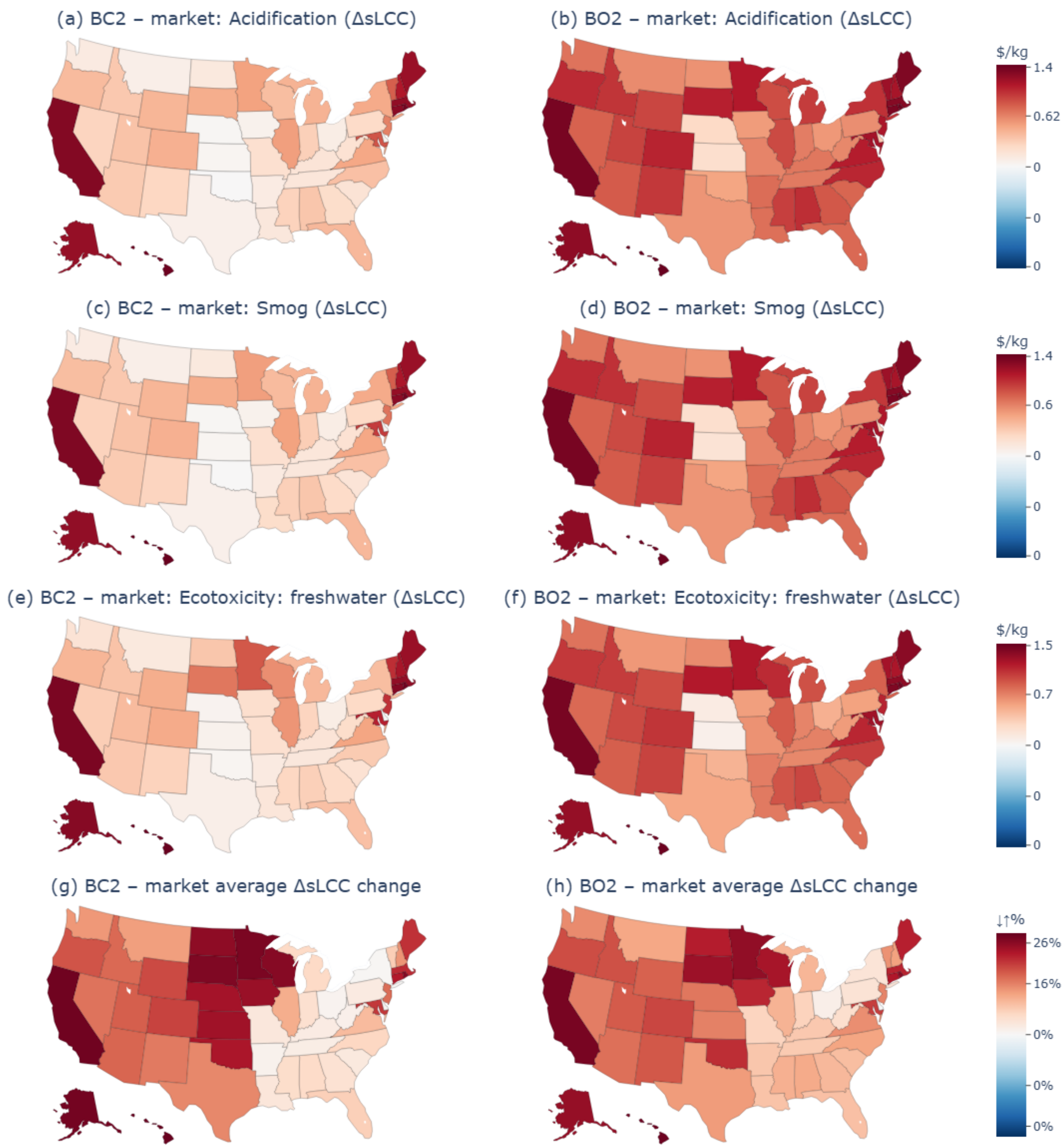

Figure S 26 Societal life cycle costing from bio-graphite and compared to sLCC from market natural graphite. (scenario: middle damage cost, 3% social rate)

(a-f): absolute difference between bio-graphite sLCC and market natural graphite sLCC for impact category: acidification, smog, ecotoxicity: freshwater. (g-h): average change of bio-graphite sLCC and market natural graphite sLCC (%) across all 6 impact categories: climate change, eutrophication, respiratory effects, acidification, smog, freshwater ecotoxicity.

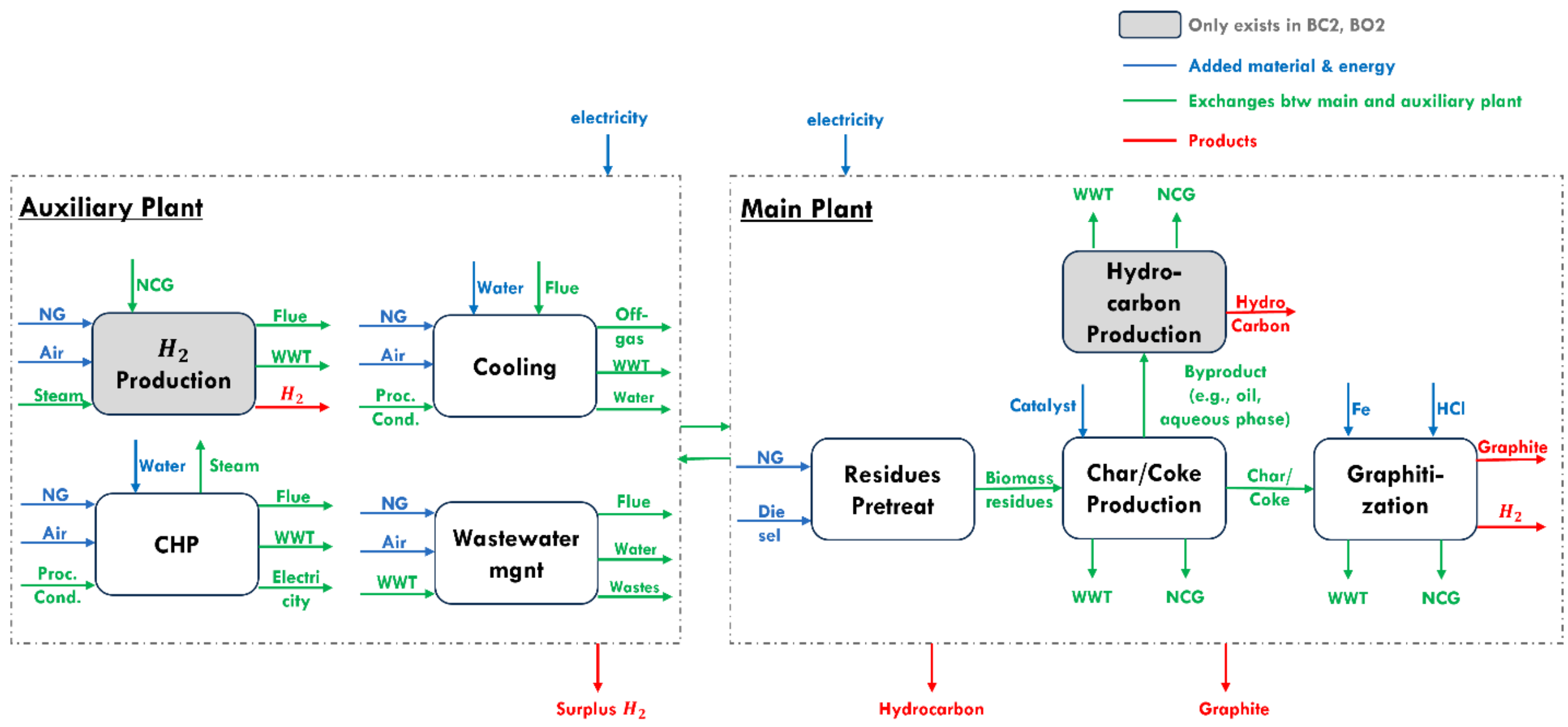

Figure S 27 Bio-graphite production overview across pathways. H_2 production and Hydrocarbon production only exist in BC2 and BO2. NG: natural gas. WWT: wastewater. CHP: combined heat and power generation. Proc. Cond.: process condensates

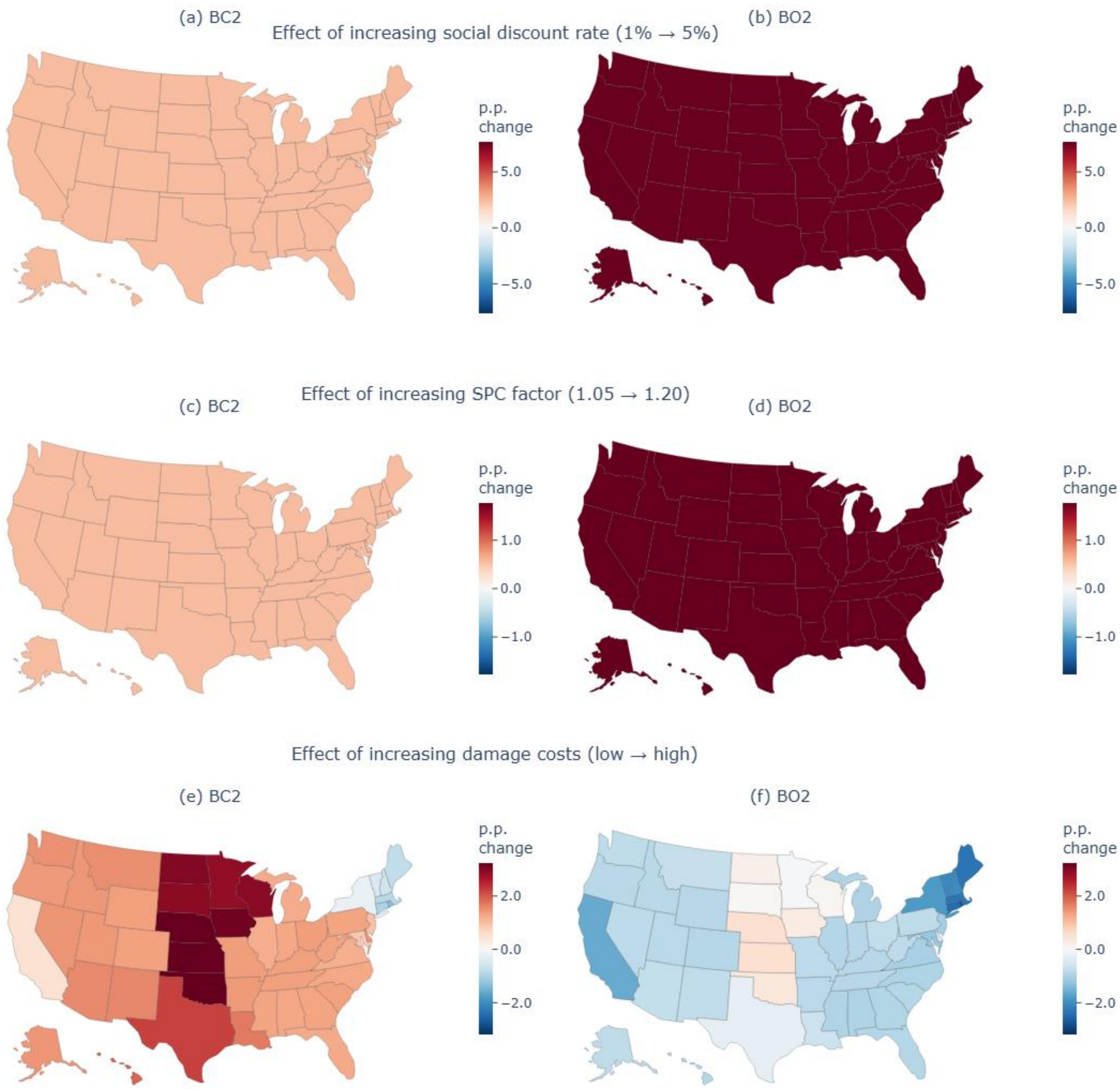

Figure S 28 Sensitivity analysis of various assumptions on social discounting rate, SPC, environmental damage costs for BC2 and BO2. p.p. change: percentage-point differences between scenarios.

The figure shows how increasing each assumption in sLCC calculation affects average $\Delta sLCC$ (i.e., sLCC change of bio-graphite v.s. market synthetic graphite presented in % and average across impact categories.). For example, (a-b) shows the shows $\Delta sLCC$ change when increase social discount rate from 1% to 5%. (c-d) shows $\Delta sLCC$ change when increase SPC factor from 1.05-1.2. (e-f) shows average $\Delta sLCC$ change across impact categories when using higher environmental

damage cost, where the low-end environmental damage costs and high-end environmental damage costs were defined in *Table S 6*. Deeper red or deeper blues means the $\Delta sLCC$ is more sensitive to the assumption change. More details are discussed in *Note S 1*

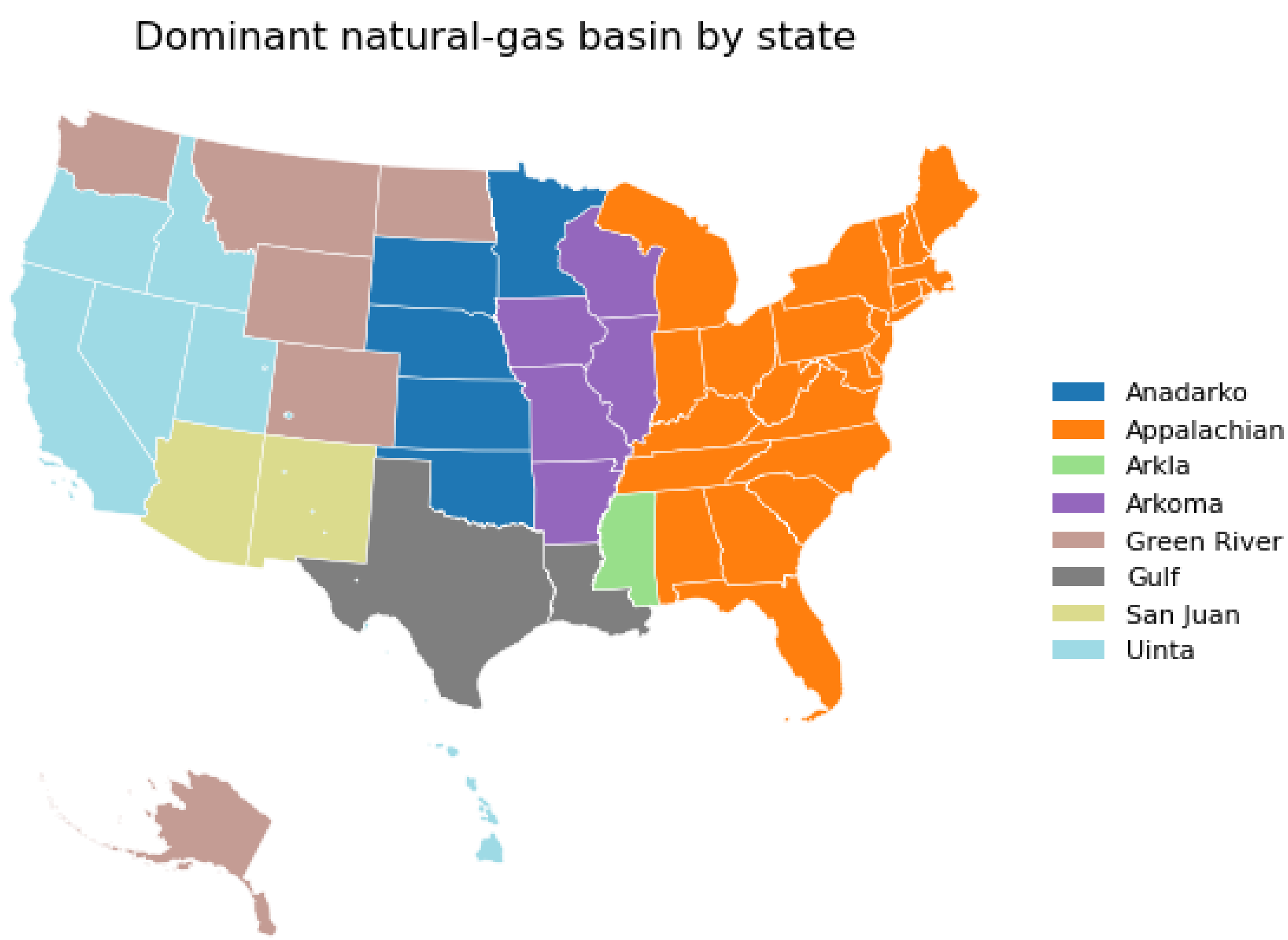

Figure S 29 Dominant Natural Gas basin by state

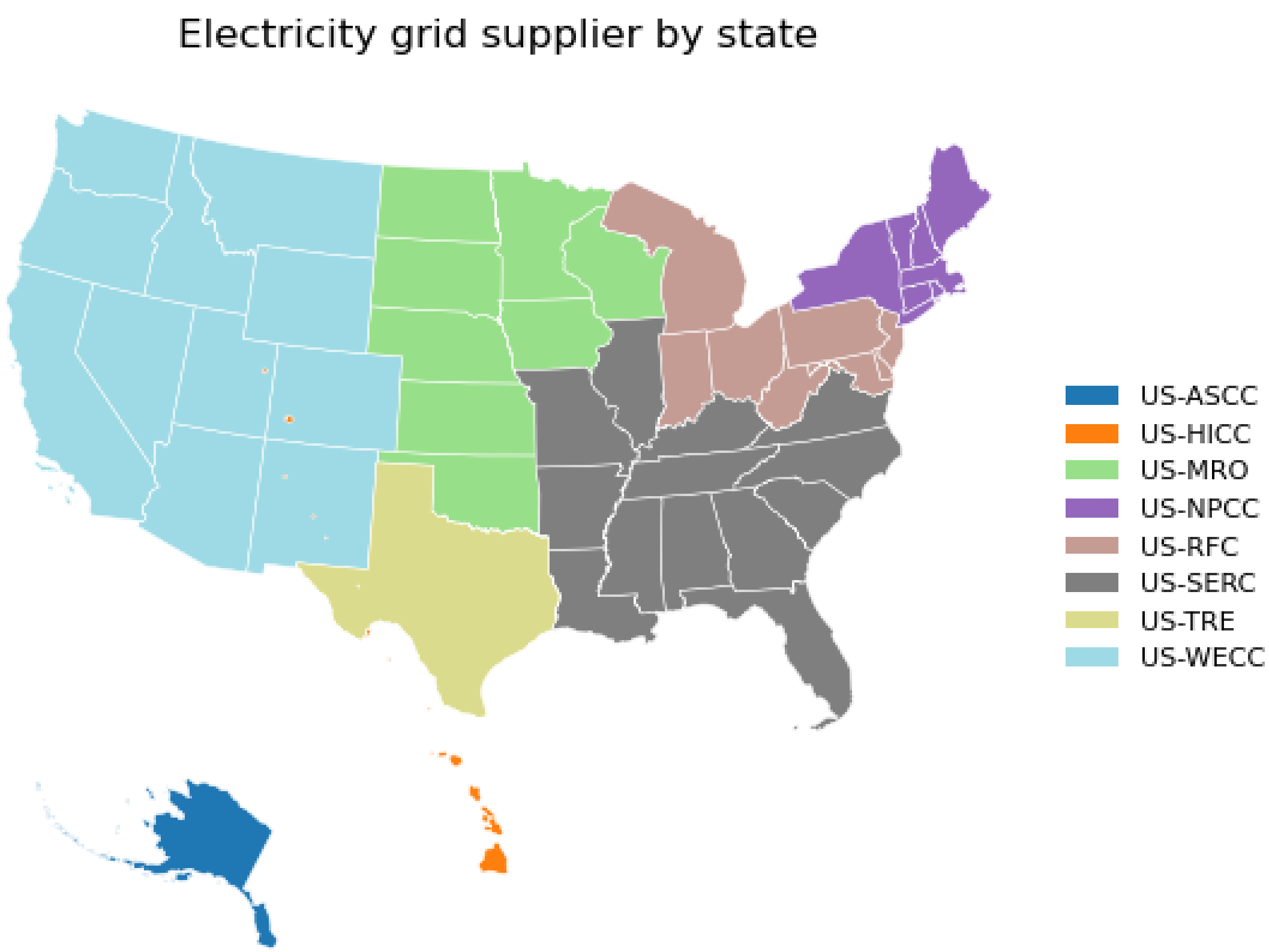

Figure S 30 Electricity grid supplier by state

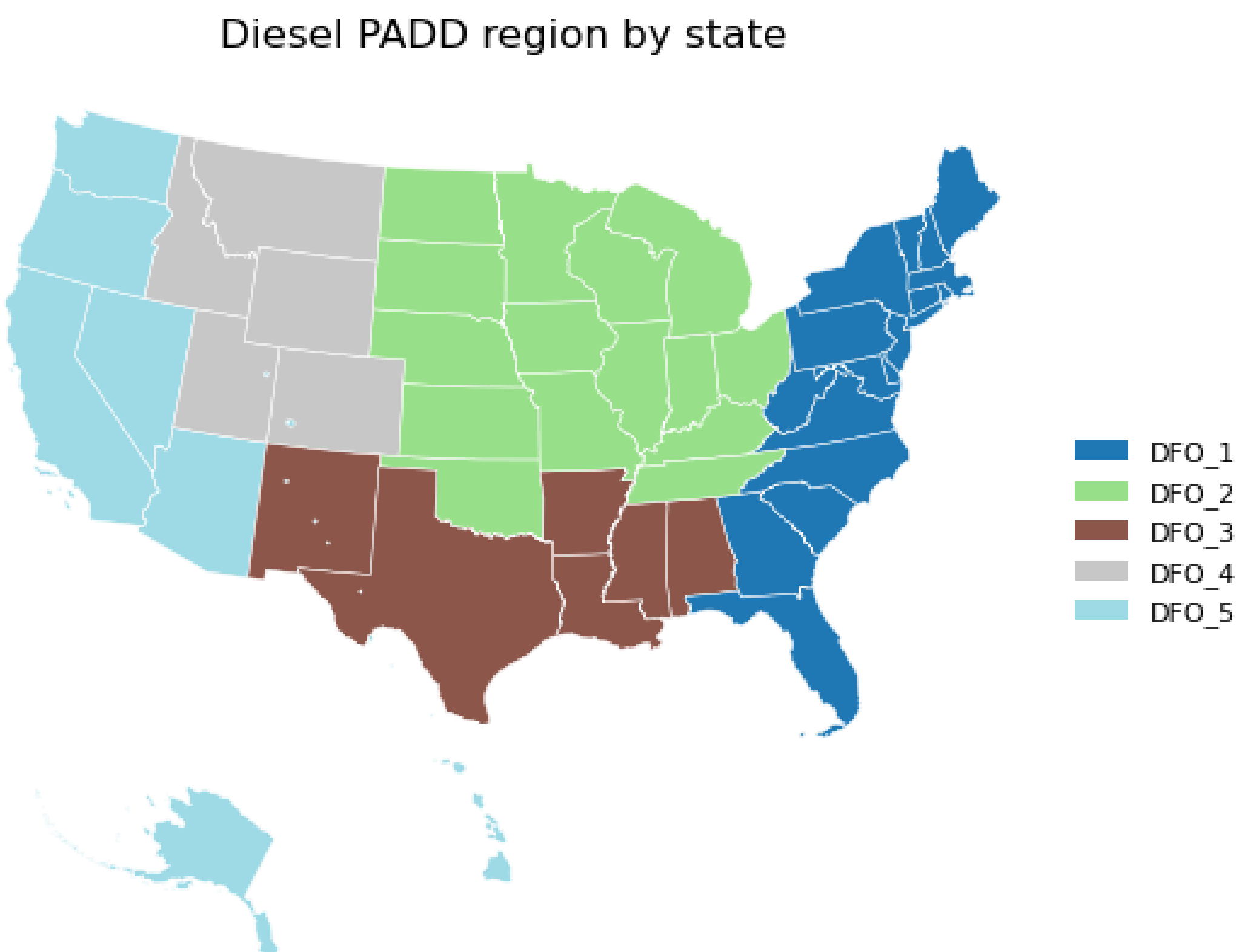

Figure S 31 Diesel PADD region by state

Supporting information – Tables

Table S 1 Billion-Ton (BT23) forest residue forecast[53]

BT23 forest resource	Market scenario	BT23 time definition	Potential (million dry short tons yr^{-1})	Maximum roadside/shadow price (2022 $/dry short ton)	Short-ton to metric-ton factor	Potential (million dry metric tonnes yr^{-1})
Forest processing waste / mill residues	Near term	Approximately by 2030	1.1	54	0.907	1
Logging residues			18.5	40	0.907	16.78
Other forest waste			7.5	50	0.907	6.8
Small-diameter trees			3.1	58.54	0.907	2.81
Total			30.3	—	0.907	27.49
Forest processing waste / mill residues	Mature market	After ~10 years and before 2050	1.1	54	0.907	1
Logging residues			19.2	40	0.907	17.42
Other forest waste			7.5	50	0.907	6.8
Small-diameter trees			34.9	70.11	0.907	31.66
Total			62.7	—	0.907	56.88

Table S 2 Model Biomass Composition

Component	w.t.(%)
Feedstock composition[6–8]	
Pulpwood	45
*[a]Forestry residues	32
Switchgrass	3
*bConstruction and Demolition wood waste	20
Elemental[6,7]	
C	50.94
H	6.04
O	42
N	0.17
S	0.03
Structural component (w.t.%) (derivation documented in *Table S 9*)	
Cellulose	40.18
Hemicellulose	20.70
Lignin	29.18
Extractives	5.90
Ash[6,7]	0.9 - 1.00
*[c]Lumped chemical (w.t.%) [9,10,17,18]	
GMSW<br>Glucomannan (softwood hemicellulose)	0
LIGNIN-C<br>Carbon-rich lignin	1.18
LIGNIN-H<br>Hydrogen-rich lignin	1.18
LIGNIN-O<br>Oxygen-rich lignin	27.73
CELLULOS<br>Cellulose	41.43

MOISTURE	0
ASH	1.03
XYHW<br>Xylan (hardwood hemicellulose)	21.35
XYGR<br>Xylan (grass hemicellulose)	0
TGL<br>Triglycerides	2.03
TANN<br>Tannin	4.05

*aForestry residues: also referred to as woody residues in Idaho National Laboratory (INL)'s literature[6], which designed the feedstock blend used in this study

*bConstruction and Demolition wood waste: also referred to as Construction and Demolition Waste in INL's literature, but the woody materials within the category was assumed[6].

*cLumped chemicals are derived following Debiagi (2015)[9] using Python package chemics[10], and the components are normalized so that the summation equals to 1

Table S 3 Prices and environmental impacts of current market synthetic and natural graphite (Functional unit: 1 kg graphite, system boundary: cradle-to-gate)

**1 kg graphite**	**Acheson Process (Market synthetic graphite)[54]**		**Market Natural Graphite[55]**	
Energy source scenario	China	U.S.*	China*	U.S.*
Price (USD)[52] [low, high]	[4.2, 5.3] mean =4.75		[2.8,4.5] mean = 3.65	
Climate Change (kg CO_2-eq)	5.45	4.29	4.95	3.55
Acidification (g SO_2-eq)	32.13	81.95	33.53	16.59
Eutrophication (g N-eq)	10.97	17.5	6.96	16.54
Respiratory effects (g $PM_{2.5}$-eq)	5.08	12.83	3.89	4.323
Ecotoxicity: freshwater (CTUe)	29.63	40.23	18.2	67.42
Smog (g O_3-eq)	410.9	399.9	345.4	236.95
*Results are modeled by the authors: U.S. scenarios of market synthetic graphite are derived using the LCI provided in ecoinvent 3.9.1[56] and Kulkarni et al. (2022) [54]; U.S. and China scenarios in market natural graphite pathways are modeled with LCI provided in Engels et al. (2022) [55]				

Market synthetic graphite refers to conventional battery-grade synthetic graphite produced from petroleum-coke-based carbonaceous feedstock through energy-intensive thermal processing, including calcination/baking and high-temperature graphitization (the Acheson Process). Market natural graphite refers to graphite obtained through mining and subsequent processing into battery-grade graphite.

Table S 4 Variable distributions in sensitivity analysis and Monte-Carlo Simulations

**Price-related variables**[*a]						
Variable	min	Max	Unit	Path	Description	Ref
Pc-biogasoline	1.79	1.9	$/gallon	BC2, BO2	Use the price range of petroleum-derived gasoline	[57]
Pc-biodiesel	4.95	5.45	$/gallon	BC2, BO2		[58]
Pc-SAF	3.93	5.85	$/gallon	BC2, BO2		[59]
Pc-bioH2	1.5	2.9	$/kg	BC2, BO2	Market price of H_2 produced with carbon capture and storage	[60,61]
Pc-e	5.88	22.15	¢/kWh	All	U.S. market industrial electricity price. The maximum price exists in Hawaii (31.87 ¢/kWh). If one excludes Hawaii, the maximum price is 22.15 ¢/kWh	[62,63]
Pc-H_2	1	3.5	$/kg	BC2, BO2	Market H_2 price	[61]
Pc-diesel	0.94	1.13	$/kg	All		[64]
Pc-N_2	0.51	1.78	$/L			[65]
Pc-NG	0.1123	0.2055	$/m^3			[66]
Pc-feed	0.0233	0.088	$/kg		State-specific feedstock price, projected residue price from forestland in U.S. billion-ton study,	[6,53,67]

					and estimates from Idaho National Laboratory (INL)	
Pc-Fe	0.537	0.806	$/kg			±20%
**Life cycle inventory (LCI)-related variables (based on 1 kg of bio-graphite)**						
Variable	min	Max	Unit	Path	Description	Ref
LCI-Bdsl	0.007	0.036	kilogram	All	Diesel usage in upstream biomass production, ±50% from literature	Diesel usage variation in upstream biomass production is based on [68]
[*f]LCI-Gre	4	10	kWh	BC1, BC2	Graphitization electricity usage (BO paths range reflects that CHP electricity generation reduces grid electricity input)	[7,69]
	0.5	7	kWh	BO1, BO2		
LCI-Pye	0.35	0.7	kWh	BC1, BC2	Electricity usages in pyrolysis and pretreatment	[8,11,70,71]
	2.39	3.19	kWh	BO1, BO2		
LCI-Fe	0.12	3.1	kg	BC1, BC2	Upper bound is when no recycling is assumed, lower bound is when 95% of Fe is recycled	Fe recycling potential is based on [7]
	0.13	3.1	kg	BO1, BO2		
ByP-HyC	0.237	0.44	kg	BC2		

	0.503	0.933	kg	BO2		±30%
Yld-CG	69	80	%	BC1, BC2	Biochar to graphite yield	[24,27,72,73]
	70	80	%	BO1, BO2	Biocoke to graphite yield	[7,23,74]
Yld-BC	25	35	%	BC1, BC2	Biomass to biochar yield	[26,27,73]
	10	20	%	BO1, BO2	Biomass to biocoke yield	[7,23,75]
**Emission-factor related variables**[*b]						
Variable	min	Max	Unit	Pathway	Description	Reference
[*c]EF-feed (per kg feedstock)	0.0955	0.177	kg CO_2-eq	All	Climate change	±30%
	0.136	0.253	g N-eq		Eutrophication	
	-0.064	-0.034	CTUe		Ecotoxicity-freshwater	
	0.159	0.296	g SO_2		Acidification	
	0.0114	0.0212	g PM2.5		Respiratory effects	
	2.69	5	g O_3-eq		Smog	
EF-e (per kWh)	0.232	0.895	kg CO_2-eq		Climate change	Ecoinvent v3.9.1 U.S. regional grid[56]
	0.1	5.1	g N-eq		Eutrophication	
	1.93	6.98	CTUe		Ecotoxicity-freshwater	
	0.254	5.731	g SO_2		Acidification	
	0.039	2.1	g PM2.5		Respiratory effects	
	6.4	64.38	g O_3-eq		Smog	
[*e]EF-NG (per MJ)	0.0142	0.02542	kg CO_2-eq		Climate change	EPA-NETL[49]
	5.09	13.2	mg N-eq		Eutrophication	
	0.0062	0.072	CTUe		Ecotoxicity-freshwater	
	0.082	0.21	g SO_2		Acidification	

	1.6	4.0	mg PM2.5		Respiratory effects	
	2.85	7.33	g O_3-eq		Smog	
*[d]EF-diesel (per MJ)	0.01609	0.02061	kg CO_2-eq		Climate change	EPA-NETL[49]
	1.039	1.548	mg N-eq		Eutrophication	
	0.00584	0.01084	CTUe		Ecotoxicity-freshwater	
	0.01567	0.02482	g SO_2		Acidification	
	1.15	1.648	mg PM2.5		Respiratory effects	
	0.4249	0.6178	g O_3-eq		Smog	

*[a]Pc: price

*[b]EF: emission factor

*[c]EF-Feed: feedstock environmental impact is modeled with a mixture of woody biomass production activities, to reflect the typical lignocellulosic waste compositions used in past literature[7,20,24]. Among the components of the feedstock mixture, the environmental impact from eucalyptus residue was identified as influential in sensitivity analysis; thus, here we vary the emission factor from eucalyptus residues production in ecoinvent v3,9.1 to represent uncertainty in EF-Feed.

*[d]EF-diesel: diesel LHV 42.8 MJ/kg; emission factor ×42.8 to derive for per kg diesel

*[e]EF-NG: natural gas LHV 35.8 MJ/m3: emission factor ×35.8 to derive for per m3 natural gas

*[f]BO1 and BO2's LCI-Gre were determined with conventional graphitization electricity usage subtracting electricity generated on-site

Note: Catalytic graphitization has been demonstrated using various Fe-based precursors, including Fe powder, $FeCl_3$, $Fe(NO_3)_3$, and Fe_2O_3[76–78]. Fe powder was selected as the graphitization catalyst in this study because of its demonstrated effectiveness[76] and its use in the experimental and process-design studies underlying the modeled pathways[7,23,27]. Because no dedicated iron-powder production dataset was identified in ecoinvent v3.9.1, the upstream

environmental burden of the Fe-based catalyst was approximated using the ecoinvent $FeCl_3$ inventory[56]

Table S 5 State feedstock residue prices, approximate with Industrial Wood and biomass waste price data from 2023 EIA State Energy Data System (SEDS) [79]

Original data is in 2023 USD, all calculations were converted into 2025 USD using producer price index (PPI) in the main text

State	Dollars per million Btu, 2023 USD	*Dollars per short ton biomass, 2023 USD
Alabama	3.32	58.0336
Alaska	1.52	26.5696
Arizona	3.01	52.6148
Arkansas	3.26	56.9848
California	3.15	55.062
Colorado	3.05	53.314
Connecticut	2.73	47.7204
Delaware	1.17	20.4516
Dist. of Col.	2.65	46.322
Florida	2.57	44.9236
Georgia	3.3	57.684
Hawaii	2.98	52.0904
Idaho	3.31	57.8588
Illinois	2.32	40.5536
Indiana	2.61	45.6228
Iowa	2.65	46.322
Kansas	1.21	21.1508
Kentucky	3.05	53.314
Louisiana	3.15	55.062
Maine	3.28	57.3344
Maryland	2.65	46.322

Massachusetts	2.44	42.6512
Michigan	2.96	51.7408
Minnesota	3.27	57.1596
Mississippi	3.29	57.5092
Missouri	2.72	47.5456
Montana	2.8	48.944
Nebraska	1.33	23.2484
Nevada	3.01	52.6148
New Hampshire	1.68	29.3664
New Jersey	2.2	38.456
New Mexico	3.45	60.306
New York	2.75	48.07
North Carolina	3.29	57.5092
North Dakota	2.53	44.2244
Ohio	2.31	40.3788
Oklahoma	2.64	46.1472
Oregon	3.18	55.5864
Pennsylvania	2.24	39.1552
Rhode Island	2.59	45.2732
South Carolina	3.28	57.3344
South Dakota	3.23	56.4604
Tennessee	3.06	53.4888
Texas	2.65	46.322
Utah	3.11	54.3628
Vermont	3.05	53.314
Virginia	3.26	56.9848

Washington	3.22	56.2856
West Virginia	2.5	43.7
Wisconsin	2.96	51.7408
Wyoming	2.94	51.3912
*Original data is in Dollars per million Btu, unit conversion: 1 short ton = 17.48 MMBtu[80]		

Table S 6 Societal cost of various environmental damages [3]

Impact category	Low societal cost scenario	*[b]Middle social cost scenario	High societal cost scenario	Unit	Quote year
Climate change	114	212.5	311	$/t CO2	2019
Acidification	239	411.5	584	$/t SO2	2019
Eutrophication	20.1	24.96	29.8	$/kg N	2019
Freshwater ecotoxicity	1.58	4.18	6.78	$/kg 2,4 – D*[a]	2019
Respiratory effects	550	607	664	$/kg PM2.5	2019
Smog	0.006	0.2445	0.483	$/kg O_3	2019

*[a]Conversion factor for 1 kg 2, 4-D: 1 kg 2, 4-D = 860.3533 CTUe[81]

*[b]Middle scenario = arithmetic midpoint of the low and high damage-cost estimates

Table S 7 BC and BO paths main product yield summary, with 2000 dry t/d (≈ 83333.3 kg/h) of biomass being processed, with 416.67 kg/h N_2 providing an inert environment. Excluding catalysts and chemicals needed.

N_2 is assumed to be purchased as bulk liquid N_2 with the supplier providing storage and vaporization systems[82,83]. Such vaporizers can operate without fuel or purchased heating energy [84].

		BC1	BC2	BO1	BO2
Pyrolysis<br>Inputs: biomass, N_2 for slow pyrolysis; biomass for fast pyrolysis					
Biochar	kg/h	21868		20000	
Liquid	kg/h	62011.5*	Oil: 8556.0	Oil: 43375	
			Aqueous: 16681.2	Aqueous: 9125	
Gas	kg/h		36774.3	10833.3	
Biochar	yield, %	~ 26%	~26%	~24 %	
Liquid	yield, %	~74 %	Oil: ~10%	Oil: ~ 52%	
			Aqueous: ~20%	Aqueous: 11%	
Gas	yield, %		~44 %	~13 %	
Delayed Coking<br>Inputs: oil phase from BO1, BO2, N_2					
Coke	kg/h	Not applicable		12250	

<table>
<tr><td>Coke Vapor</td><td>kg/h</td><td colspan="2" rowspan="4"></td><td colspan="2">31541.67</td></tr>
<tr><td rowspan="2">Coke</td><td rowspan="2">yield, %</td><td colspan="2">Oil-to-coke: 28%</td></tr>
<tr><td colspan="2">Biomass-to-coke:14.7 %</td></tr>
<tr><td>Coke Vapor</td><td>yield, %</td><td colspan="2">Oil-to-vapor: 72 %</td></tr>
<tr><td colspan="6">Graphitization

Input: sieved biochar/biocoke (around 4.5% of the biochar/biocoke was lost as fines)
Assume around 95wt% biochar C converts to graphite[72]
Modeled biochar C: 84.4 w.t.%</td></tr>
<tr><td>Graphite</td><td>kg/h</td><td colspan="2">16814.75</td><td colspan="2">9458.3</td></tr>
<tr><td>Graphite</td><td>yield, %</td><td colspan="2">Biomass-to-graphite: 20.2%

Biochar-to-graphite:76.9%</td><td colspan="2">Biomass-to-graphite: 11.3%

Biocoke-to-graphite: 77.2%</td></tr>
<tr><td colspan="6">Hydrotreating, hydrocracking, fractionation

Inputs: in BC2, use oil phase from pyrolysis vapor; in BO2, use oil and aqueous phase from coke vapor</td></tr>
<tr><td>Hydrocarbon</td><td>kg/h</td><td rowspan="3">Not applicable</td><td>5697.6</td><td rowspan="3">Not applicable</td><td>6791.7</td></tr>
<tr><td rowspan="2">Hydrocarbon</td><td rowspan="2">yield, %</td><td>Biomass-to-hydrocarbon: 6.8 %</td><td>Biomass-to-hydrocarbon: 8.15%</td></tr>
<tr><td>Biooil-to-hydrocarbon: 66.6%</td><td>Biooil (oil phase)-to-hydrocarbon: 66.7%</td></tr>
</table>

					Biooil (aqueous phase)-to-hydrocarbon: 27.5 %		
Hydrocarbon ratio			Overall		Oil phase	Aq. phase	Overall
Gasoline ratio	%		60.52		43	0	7.4
Diesel ratio	%		8.23		15	46	40.7
SAF ratio	%		31.25		42	54	51.9
Process condensates (theoretically inputs to CHP)							
Condensates (those consist of organics that can theoretically input to CHP)	kg/h	0	26,878 (process condensates)	41,917 (process condensates) 888.06 (dilute from wastewater treatment)	59,541 (process condensates) 748.24 (dilute from wastewater treatment)		
* In BC1 pyrolysis, liquid and gas are considered as vapor altogether, which is combusted as energy *Yields (%) in pyrolysis is represented on biomass basis							

Table S 8 Capital cost (CAPEX) from each processing stage and pathway

Process	Installed capital Cost (2025$)	Scenario
Feed handling and Drying	$544,042.15	BO2
Fast Pyrolysis	$173,836,580.78	
Delayed Coking	$46,119,017.90	
Oil phase hydrotreating	$33,048,562.14	
Hydrocracking	$4,958,791.84	
Hydrogen production	$46,179,418.90	
Graphitization and post-treating	$55,615,998.27	
Wastewater treatment	$23,714,554.28	
Cooling water plant and other utilities	$41,376,287.35	
Steam system and power generation	$65,267,737.43	
Aqueous phase upgrading	$18,710,186.23	
**Total installed cost of BO2**	$509,371,177.27	
Feed handling and Drying	$544,042.15	BO1
Fast Pyrolysis	$173,836,580.78	
Delayed Coking	$46,119,017.90	
Graphitization and post-treating	$55,615,998.27	
Wastewater treatment	$19,707,521.93	
Cooling water plant and other utilities	$35,851,727.45	
Steam system and power generation	$25,309,348.02	
**Total installed cost of BO1**	$356,984,236.50	
Feed handling and Drying	$544,042.15	BC2
Slow Pyrolysis	$72,699,897.41	
Condensation	$8,354,753.27	
Two-stage Hydrotreating	$26,159,778.77	
Oil Fractionation	$2,103,799.42	
Hydrogen production	$69,350,090.24	
Graphitization and post-treating	$55,615,998.27	

Wastewater treatment and Steam generation	$34,633,786.05	
Cooling water & Other Utilities	$6,589,760.99	
**Total installed cost of BC2**	$276,051,906.57	
Feed handling and Drying	$544,042.15	BC1
Slow Pyrolysis	$72,699,897.41	
Graphitization and post-treating	$55,615,998.27	
Wastewater treatment	$3,468,036.90	
Cooling water & Other Utilities	$3,909,102.44	
**Total installed cost of BC1**	$136,237,077.17	

Table S 9 Model biomass structural composition derivation

model biomass	pulpwood (pine)	poplar	switchgrass	Construction & demolition waste	*Woody residues	*Biomass blend (this study)
Cellulose (wt%)	41.37	43.78	33.21	34.7	42.58	40.18
Hemicellulose (wt%)	22.21	21.71	29.42	14	21.96	20.70
Lignin (wt%)	30.5	25.7	16.24	29.9	28.10	29.18
Extractives (wt%)	5.15	3.98	9.36	9.2	4.57	5.90
Ash (wt%)	0.27	0.24	1.88	3.7	0.26	1.00
reference	[85]			[86]	calculated	calculated

*woody residues: average from pulpwood and poplar

*biomass blend: weighted average from pulpwood, woody residues, switchgrass, construction & demolition waste according to the composition of the representative lignocellulosic residues, i.e., the weight of each component is 45 wt%, 32 wt%, 3 wt%, 20 wt%, respectively

Table S 10 Past studies on biochar and biocoke converted to bio-graphite production conditions

Study	Feedstock	Pyro-lysis temp-erature*	Pyro-lysis heating rate	Pyro-lysis resid-ence time	Graphit-ization Precursor	Graphit-ization temp-erature (°C)	Yield (w.t.%)
Shi et al. (2023)[73]	Pine and spruce sawdust	500 °C	Slow pyrolysis (rate not specified)	Not specified	Biochar	1300	9.5 (from biomass)
Shi et al. (2024)[20]	Softwood sawdust	550 °C	Slow pyrolysis (rate not specified)	Not specified	Biochar	1300	
Shi et al. (2026)[74]	Biooil	600 °C (1-step graphit-ization)	5 °C/min	1 h	Biooil	1300	18.41 (from heavy fraction of biooil)
Sagues et al. (2020)[87]	Various biomass (Softwood, Lignin)	600 °C (1-step graphit-ization)	3 °C/min	30 min	Raw Biomass	1200	18 (from biomass)
Dey et al. (2024)[23]	Hardwood –pyrolysis oil	600 °C (1-step graphit-ization)	Coking rate 4 °C/min	Not specified	Pyrolysis oil	1500	

Dey et al. (2025)[75]	Hardwood sawdust	Coking at 300 - 500 °C	Coking rate 4 °C/min	Coking for 1 h	Biocoke	1500	
Banek et al. (2018[72], 2022[25])	Hardwood sawdust	600 °C	30 °C/min	30 min	Biochar	1500+ (Laser)	95.7 (from biochar C content)
Vook et al. (2023)[26]	Paper towel waste	1000°C	5 °C/min	Not specified	Biochar	1100-1300	14.27 (from biomass)
You et al. (2024)[27]	Hardwood chips	700-1200°C	10 °C/min	1 h	Biochar	1200	69 (from biochar)
Béguerie et al. (2023)[88]	Cellulose and kraft lignin	800 °C	2 °C/min	1 h	Biochar	1000 – 1800	
Lee et al. (2024)[89]	Oak and bamboo Charcoal	Not specified	Not specified	Not specified	Biochar	2400	86 (evaluated at 1500 °C, from charcoal)
Gulas et al. (2026)[24]	Various woody biochar	Not specified	Not specified	Not specified	Biochar	2800 – 3000	80 (from biochar)

*1-step graphitization: graphitization carries out from feedstock to graphitize directly, without cooling to room temperature after intermediate char/coke is produced

*2-step graphitization: feedstock is produced into biochar or biocoke first; then, then, temperature cools to room temperature, and graphitization ramp up again to 1000 +°C for graphitization

Table S 11 Proximate and ultimate analysis of biocoke obtained at 500 °C under Nitrogen atmosphere without catalyst [7]

**Ultimate analysis**	
Component	Weight Percentage (w.t % dry basis)
Carbon	81.78
Hydrogen	3.42
Nitrogen	0.64
Oxygen	14.16
**Proximate analysis**	
Component	Weight percentage (w.t.%)
Fixed Carbon and Ash	67.47
Volatile Matter	27.62
Moisture	4.91

Table S 12 Electricity usage of the market and bio-graphite at the graphitization stage per kg of graphite

Graphite type	Electricity usage at graphitization stage (kWh/kg graphite)	Reference
Natural Graphite	3.3	[54,55]
Synthetic Graphite	Electrochemical process:<br>0.25<br>Acheson process:<br>4 – 12	[54]
Biochar-derived bio-graphite	4 – 10	[7]
Biocoke-derived bio-graphite	2 – 9	

Table S 13 BC paths: Modeled biochar, biooil elemental composition from slow pyrolysis and condensation

**Biochar**		
Component	Value	Description
Carbon (dry w.t.%)	84.4	Similar elemental compositions have been observed for woody biochar derived from 600℃[16,72]. C: 82 – 87.74 w.t.%; H: 2 – 2.82 w.t.% O: 4.5 – 15.3 w.t.%; Ash: 2.22 – 4.3 w.t.%
Hydrogen (dry w.t.%)	3.3	
Oxygen (dry w.t.%)	8.1	
Ash (dry w.t.%)	3.8	
H/C	0.46	
O/C	0.10	
Fixed Carbon (dry w.t.%)	84.4	Ratio of Fixed Carbon/Carbon ≈0.99-1.01 for woody biochar obtained from 550-600 ℃ pyrolysis[16,90], indicating that the material is highly carbonized at this temperature. Thus, we assume the ratio is 1 here.
**Bio-oil**		
Component	**Value**	The modeled biooil elemental composition is within a similar range as mentioned in [74,91–93] C: 63.3 – 73.5 w.t.%, H: 7 – 8.5 w.t.%, O: 25-28 w.t.%
Carbon (w.t.%)	64.4	
Hydrogen (w.t.%)	7.0	
Oxygen (w.t.%)	28.6	
H/C	1.73	
O/C	0.33	

Table S 14 Capital investment breakdown by process sections for BC2 based on past literature [7,8,94]

Equipment title	Install Factor	Purchase equipment cost Unit: 2025 $ k USD		Installed cost Unit: 2025 $ k USD	
Flue Gas Blower	1.94	$	156.13	$	302.89
Cross Flow Pellet Dryer	2.00	$	120.57	$	241.15
Feed handling and Drying Total		$	276.70	$	544.04
Pyrolyzer + Combustor + Grinder + Storage + Cleaning	1.00	$	72,699.90	$	72,699.90
Slow Pyrolysis Total		$	72,699.90	$	72,699.90
Heavy Fraction Condenser Absorber	1.40	$	1,084.15	$	1,517.81
Light Fraction Condenser Absorber	1.33	$	1,101.38	$	1,464.84
Vapor ACHE	1.32	$	325.94	$	430.24
Feed Cooler	2.03	$	75.62	$	153.52
Feed/overhead exchanger	1.52	$	401.33	$	610.02
Liquid feed chiller	2.33	$	59.45	$	138.53
Heavy fraction ACHE	2.42	$	33.61	$	81.34
Quench circulation chiller	4.63	$	3.03	$	14.02
Bottoms pump	4.38	$	5.49	$	24.04
Circulation filter charge pump	4.61	$	0.57	$	2.62
Aqueous filter charge pump	4.20	$	1.46	$	6.11
Organic product pump	4.63	$	2.97	$	13.74
Quench recycle pump	4.88	$	0.19	$	0.93
Bottoms filter	1.84	$	7.96	$	14.64
Bottoms decanter	1.82	$	10.79	$	19.64
Quench circulation filter	1.71	$	1.85	$	3.16
Aqueous phase filter	1.77	$	5.14	$	9.10
Organic product surge tank	3.24	$	24.24	$	78.54
Circulation surge tank	3.24	$	2.48	$	8.04
Pinch exchanger CAPEX	2.64	$	1,425.70	$	3,763.85
Condensation Total		$	4,573.36	$	8,354.75
Feed Pump	1.35	$	84.93	$	114.66
Stabilizer	2.75	$	2,232.49	$	6,139.35
1st stg Reactor	2.75	$	803.22	$	2,208.85
2nd stage Reactor	2.75	$	5,049.12	$	13,885.09
Sulfur Agent Pump	1.79	$	1.35	$	2.42
Stabilizer Product Flash	3.02	$	15.36	$	46.40

2nd stg Three Phase Separator	1.40	$	437.23	$	612.12
Hydrotreating Oil Flash	1.12	$	203.18	$	227.56
Expander	1.12	$	467.71	$	523.84
Stabilized Oil Cooler	1.29	$	34.53	$	44.54
1st Feed/Product Heat Recovery	3.35	$	74.78	$	250.51
2nd Feed/Product Heat Exchanger	2.64	$	272.64	$	719.78
HT Oil Product Heat Recovery	6.44	$	24.33	$	156.69
HT Oil Product Cooler	3.86	$	41.40	$	159.80
Fired Heater - startup	1.14	$	84.14	$	95.92
Air Cooler	1.29	$	66.26	$	85.47
Stabilized Product Pump	1.39	$	109.01	$	151.52
Stabilizer gas product compressor	1.09	$	332.75	$	362.70
Hydrogen Compressor	1.09	$	341.80	$	372.57
Two-stage Hydrotreating Total		$	10,676.24	$	26,159.78
Gasoline column	1.69	$	188.65	$	318.82
Diesel column	1.91	$	104.70	$	199.98
Condenser (startup)	4.58	$	13.24	$	60.62
Reboiler (startup)	2.83	$	20.67	$	58.49
Condenser (startup)	6.66	$	5.19	$	34.60
Reboiler (startup)	2.83	$	10.36	$	29.32
Gasoline product cooler	4.04	$	7.23	$	29.20
Diesel Product Cooler	5.63	$	2.14	$	12.05
Reflux pump	4.31	$	6.05	$	26.07
Reflux pump	3.96	$	1.25	$	4.96
Overhead accumulator	4.01	$	26.67	$	106.94
Overhead accumulator	4.34	$	6.07	$	26.32
Pinch exchanger CAPEX	2.64	$	453.19	$	1,196.42
Oil Fractionation Total		$	845.40	$	2,103.80
WGS Effluent ACHE	1.63	$	77.43	$	126.21
WGS Effluent cooler	3.60	$	19.31	$	69.51
Non-condensable gas compressor to reformer	1.60	$	1,746.66	$	2,794.66
Compressor condensate pump	4.38	$	4.82	$	21.12
Steam reformer system	1.92	$	30,011.58	$	57,622.24
LTS reactor	1.42	$	383.45	$	544.50
Compressor condensate tank	3.24	$	21.68	$	70.25
Pinch exchanger CAPEX	2.64	$	3,068.79	$	8,101.61
Hydrogen production Total		$	35,333.72	$	69,350.09

Reaction hydrogen - melt salt mixing tank	1.00	$	243.41	$	243.41
PSA reaction Compressor 1	1.00	$	2,601.35	$	2,601.35
PSA reaction Compressor 2	1.00	$	884.48	$	884.48
PSA reaction Compressor 3	1.00	$	863.44	$	863.44
PSA reaction Compressor 4	1.00	$	821.37	$	821.37
PSA reaction Compressor feeding Tank	1.00	$	125.21	$	125.21
PSA reaction Compressor cond. Tank	1.00	$	127.21	$	127.21
Reaction Iron makeup mixing kneader	1.00	$	295.49	$	295.49
Reaction Hydrochloric acid - iron separation cyclone	1.00	$	95.16	$	95.16
Reaction Gas phase reduction reactor	1.00	$	24,844.54	$	24,844.54
Salt melting steam mixing tank	1.00	$	144.24	$	144.24
Postreatment dryer	1.00	$	206.35	$	206.35
Postreatment separation vortex	1.00	$	14.02	$	14.02
Postreatment Biographite shaping mill	1.00	$	453.76	$	453.76
Reaction cyclone blower	1.00	$	259.43	$	259.43
Postreatment vortex blower	1.00	$	36.06	$	36.06
Acid washing centrifuge	1.00	$	7,238.10	$	7,238.10
Acid - iron - biographite premixing tank	1.00	$	242.41	$	242.41
Acid washing reactor	1.00	$	403.68	$	403.68
PSA reaction condensates tank	1.00	$	124.21	$	124.21
PSA reaction compressor knock out tank	1.00	$	124.21	$	124.21
PSA recycle condensates tank	1.00	$	125.21	$	125.21
PSA recycle compressor knock out tank	1.00	$	125.21	$	125.21
Pretreatment coke comminuting mill	1.00	$	331.55	$	331.55
Pretreatment coke recycle kneader	1.00	$	382.64	$	382.64
Pretreatment screen 1	1.00	$	32.05	$	32.05
Pretreatment screen 2	1.00	$	32.05	$	32.05
Graphitization Iron - Coke mixing kneader	1.00	$	382.64	$	382.64
PSA recycle Compressor 1	1.00	$	941.57	$	941.57

PSA recycle Compressor 2	1.00	$	476.80	$	476.80
PSA recycle Compressor 3	1.00	$	468.78	$	468.78
PSA recycle Compressor 4	1.00	$	478.80	$	478.80
PSA recycle Compressor cond. tank	1.00	$	122.20	$	122.20
PSA recycle condensates tank	1.00	$	203.34	$	203.34
Recycle Hydrochloric acid knock out tank	1.00	$	232.39	$	232.39
Recycle Water - Hydrochloric acid mixing tank	1.00	$	237.40	$	237.40
Recycle Hydrochloric acid makeup mixing tank	1.00	$	232.39	$	232.39
PSA recycle Compressor feeding Tank	1.00	$	125.21	$	125.21
PSA reaction Column	1.80	$	523.88	$	942.58
PSA recycle Column	1.80	$	523.88	$	942.58
Acid wash air fan	1.18	$	49.08	$	58.10
Graphitization Furnace	3.50	$	551.92	$	1,930.23
Conveyor Raw Coke	1.00	$	1,116.87	$	1,116.87
Conveyor Coke	1.00	$	394.66	$	394.66
Pinch Heat Exchanger CAPEX	2.64	$	1,952.26	$	5,152.62
Graphitization and post-treating Total		$	50,190.93	$	55,616.00
RTO blower	1.60	$	360.76	$	577.22
Clarifier	2.41	$	106.42	$	256.47
Belt press	2.47	$	142.42	$	351.79
Sand filter	2.47	$	127.02	$	313.74
Aqueous regenerative thermal oxidizer	2.40	$	8,747.50	$	20,993.99
Reverse osmosis unit	1.15	$	117.72	$	135.38
Clarifier overflow pump	4.38	$	9.22	$	40.38
Slurry pump	3.10	$	3.59	$	11.12
Boiler charge pump	4.14	$	6.66	$	27.58
Condensate pump	4.59	$	5.20	$	23.86
Boiler chage pump	4.59	$	5.10	$	23.41
Boiler makeup pump	1.84	$	30.11	$	55.41
Concentrated waste water pump	4.31	$	5.93	$	25.55
RO feed pump	3.84	$	8.08	$	31.02
Clean water pump	4.35	$	2.72	$	11.84
Clarifier overflow tank	2.27	$	87.89	$	199.50
Slurry tank	2.27	$	87.89	$	199.50
Boiler feed tank	4.49	$	9.75	$	43.80

Steam drum	4.46	$	14.55	$	64.88
Boiler feed tank	4.46	$	13.35	$	59.52
Steam drum	5.23	$	11.29	$	59.06
RO feed tank	2.24	$	50.96	$	114.15
Clean water tank	2.23	$	110.54	$	246.50
Pinch Heat Exchanger CAPEX	2.64	$	1,122.72	$	2,963.99
Booster Pump (plunger type)	2.40	$	19.63	$	47.12
Feed Pump (plunger type)	1.74	$	94.12	$	163.77
Feed/Product Exchanger	2.20	$	1,833.57	$	4,033.85
Fired Heater	1.30	$	335.88	$	436.65
CHG Reactor	2.00	$	1,357.87	$	2,715.75
Product Air Fin Cooler	1.19	$	151.28	$	180.03
CHG Product Separator	4.15	$	54.69	$	226.97
Wastewater treatment and Steam generation Total		$	15,034.44	$	34,633.79
Plant air compressor	1.57	$	291.06	$	456.97
Cooling tower system	2.47	$	119.68	$	295.62
Hydraulic truck dump with scale	2.47	$	97.09	$	239.81
Flue gas scrubber	2.47	$	215.51	$	532.30
Flue gas stack	1.30	$	57.37	$	74.59
Chilled water system	1.80	$	570.55	$	1,026.98
Product loading rack	2.47	$	24.80	$	61.26
Cooling water pump	2.14	$	392.51	$	839.96
Firewater pump	3.70	$	50.86	$	188.16
Diesel pump	5.39	$	8.48	$	45.70
Ammonia pump	5.21	$	8.48	$	44.18
Caustic pump	4.30	$	10.85	$	46.66
BFW chemical pump	5.21	$	8.48	$	44.18
Flue gas scrubber circulation pump	4.12	$	19.50	$	80.32
Gasoline product pump	3.96	$	5.78	$	22.91
Diesel product pump	3.96	$	1.67	$	6.62
Instrument air dryer	2.47	$	24.42	$	60.31
Plant air receiver	5.44	$	23.12	$	125.76
Firewater storage tank	1.46	$	253.02	$	369.41
Purchased diesel storage tank	1.35	$	115.20	$	155.52
Ammonia storage tank	5.39	$	17.28	$	93.16
Caustic storage tank	3.01	$	17.61	$	53.02
BFW chemical storage tank	6.70	$	24.22	$	162.25

Gasoline product storage tanks	1.75	$	667.85	$	1,168.75
Diesel product storage tank	1.75	$	225.92	$	395.36
Cooling water & Other Utilities Total		$	3,251.31	$	6,589.76

Table S 15 TEA cash flow analysis capital investment table[7]

Discounted Cash Flow Financial Parameters		
Equity	(% of *[a]FCI)	40%
Loan Interest		8%
Loan Term, years		10
Working Capital	(% of *[a]FCI)	5%
Discount Rate (IRR)		10%
Income Tax Rate		21%
Plant Depreciation Period		
General plant	(Years)	7
Steam plant	(Years)	20
Plant Life (Project life)	(Years)	30
Construction Period	(Years)	3
% Spent in Year -2		8%
% Spent in Year -1		60%
% Spent in Year 0		32%
Start-up Time	(Years)	0.25
Biographite production during start-up	(% of normal capacity)	50%
Variable Costs during start-up	(% of normal)	75%
Fixed Cost during start-up	(% of normal)	100%
Land Requirement	Acres	132
Land Cost	$/acre	$14,000
Other costs		
Warehouse	(% of *[b]ISBL)	4%
Site development	(% of *[b]ISBL)	9%
Additional piping	(% of *[b]ISBL)	4.5%
Field expenses	(% of *[c]TDC)	10%
Home office & construction fee	(% of *[c]TDC)	20%
Process contingency	(% of *[c]TDC)	10%

Prorated expenses	(% of *[c]TDC)	10%
Other costs (start-ups, permits, etc.)	(% of *[c]TDC)	10%
Operating hours	hours	8000[95,96]

*[a]FCI: Fixed capital investment

*[b]ISBL: Inside battery limits plant costs, representing cost of procuring and installing all process equipment

*[c]TDC: Total direct cost

Table S 16 TEA operating costs table

Labor costs[*a]				
**Position**	**Salary per position (2016 USD)**	**Number of Position**	**Total (2025 USD)**	**Ref**
Plant Manager	164,452	2	456,859.39	[7]
Plant Engineer	78,310	2	217,550.77	
Maintenance Supr	63,767	2	177,149.27	
Maintenance Tech	44,749	15	932,369.36	
Lab Manager	62,648	2	174,040.61	
Lab Technician	44,749	4	248,631.83	
Shift Supervisor	53,699	10	745,898.26	
Shift Operators	44,749	30	1,864,738.71	
Yard Employees	31,324	15	652,652.30	
General manager	101,891	1	141,530.23	
Clerks & Secretaries	36,000	5	250,026.42	
**Total salaries**	726,338	88	**5,861,447.15**	
Labor burden	90% of total salaries		5,275,302.44	
**Maintenance[7], and Property Insurance[97]**				
Maintenance	3% of FCI[*b]			
Property Insurance	1% of FCI			
**Material and energy costs**				
**Material or energy**	**Price per unit**	**unit**	**Quote year**	**Ref**
feedstock	60	\$/short ton	2023	[67,98]
diesel	3.133	\$/gallon	2025	[64]
electricity	8.62[*c]	cents/kWh	2025	[99]
N_2	1.145	\$/L	2025	[65]
natural gas	5.74	\$/1000ft^3	2025	[66]
H_2	1.5	\$/kg	2024	[61]
$NiMo/Al_2O_3$ catalyst	44444	\$/t	2015	[7,8]
ZrO_2 catalyst	50000	\$/t	2015	[7,8]

Pt/Al_2O_3 catalyst	1436000	\$/t	2015	[7,8]
Ni/Al_2O_3 catalyst	11.62	\$/kg	2025	[100]
water	5.56	\$/1000 gallon	2023	[101]
HCl	179	\$/t	2015	[7,8]
Fe	550	\$/t	2015	[7,8]
Wastewater disposal	6.86	\$/1000 gallon	2023	[101]
biogasoline	1.84	\$/gallon	2025	[57]
biodiesel	5.2	\$/gallon	2025	[58]
bioSAF	4.89	\$/gallon	2025	[59]
bioH_2	2.2	\$/kg	2024	[61]
sand	45.74	\$/t	2024	[7]
toluene	1296	\$/t	2024	[7]
H_2S	1776	\$/t	2024	[7]
NaOH	2	\$/lb	2025	[102]
boiler chemical	5557.64	\$/t	2024	[7]
cooling chemical	2.027	\$/lb[*d]	2016	[8,103]
Ar	0.55	\$/kg	2025	[104]
MgAlO catalyst	57.5	\$/kg	2025	[105]
Solid disposal	0.033	\$/kg	2024	[7]

[*a]Labor costs are adjusted to 2025 value based on indices from the U.S. Bureau of Labor Statistics

[*b]FCI: Fixed Capital Investment

[*c]Annual national average price of industrial electricity in the U.S.

[*d]Literature range: 1.7842 – 2.27 \$/lb[8,103]

Table S 17 TEA summary (CAPEX, OPEX, byproduct revenue) by pathway

Pathway	Annual graphite amount (thousand tonne/yr)	Fixed capital investment, FCI (million $)	Annual OPEX (million $/yr)	Annual by-product credit (million $/yr)	*Precursor throughput (thousand kg/h)
BC1	134.5	254.1	545.7	0	21.87
BC2	134.5	507.4	579.6	55.3	21.87
BO1	75.7	648.5	370.6	0	12.25
BO2	75.7	921.1	380	99.4	12.25

*Precursor is biochar or biocoke

Table S 18 Past studies on biochar and biocoke converted to bio-graphite material properties compared to market graphite

Study	Graphitization Precursor	Catalyst	Graphiti-zation Temp (°C)	Rever-sible Capacity (mAh/g)	Initial Effi-ciency (ICE, %)	Graphiti-zation Degree (G or α)	Surface area (m^2/g)
Shi et al. (2023)[73]	Biochar	Iron ($FeNO_3)_3$	1300	264	67	91.32% (G)	70
Shi et al. (2024)[20]	Biochar	Mixture (Ni-Fe-Mn)	1300	293	75.55	89.28 % (G)	93
Shi et al. (2026)[74]	Biooil (Best sample HFB)	Iron powder	1300	275.9	≈50	94.53 % (G)	70
Sagues et al. (2020)[87]	Biomass	Iron Powder	1200	335.09	83.96	~87 % (G)	25.99
Dey et al. (2024)[23]	Pyrolysis oil	Various iron catalysts	1500	350	90.2	> 90% (G)	1.35
Dey et al. (2025)[75]	Biocoke	Reduced Iron	1500	370	92.03	91% (α)	1.35 – 1.78
Banek et al. (2018[72], 2022[25])	Biochar	Iron Metal	1500+ (Laser)	353-356	84-92.3	High crysta-llinity	4.9 – 14.2
Vook et al. (2023)[26]	Biochar	Iron	1100 - 1300	340-355	75-77	91.4% (α)	8.02 – 9.1

You et al. (2024)[27]	Biochar	Iron Powder	1200	Not measured	Not measured	> 90% (α)[*a]	Not measured
Béguerie et al. (2023)[88]	Biochar	Calcium (CaO)	1000 - 1800	Not measured	Not measured	58.1% (G)[*b]	Not measured
Lee et al. (2024)[89]	Biochar (Bamboo)	None (Catalyst-free)	2400	196	Not measured	91.75% (α)	Not measured
Gulas et al. (2026)[24]	Biochar	None (Catalyst-free)	2800 - 3000	335.7	87.2	94-95 % (G)	6.7-12.8
Market synthetic graphite[106]	Petroleum coke, coal	None	2500 - 3000	345 – 355	92 - 93	90 -94% (G)[24]; 90 - 94% (α)[75,107]	1.5 – 2
Market Natural graphite[106]	Mined graphite ore	None	Purifi-cation / coating at 1000 - 3000	358 – 360	92 – 93.5	94 – 98% (G)[108]	2.1 – 4.85

[*a]calculated with $\alpha = \frac{I_G}{I_G + I_D} \times 100$

[*b]claculated with $G = \frac{0.3440 - d_{002}}{0.3440 - 0.3354} \times 100$, d_{002}: interlayer spacings

Table S 19 Modeled Pyrolysis reactions

Reactions inside R-102 in Figure S 5	
Reaction ID	Reactions
1	CELLULOSàCELLA
2	CELLAà0.405 CH3COOH+ 0.18 GLYOX+ 0.27 ACETONE+ 0.225 HMFU+ 0.145 H2+ 0.279 CO+ 0.419 CO2+ 0.36 HCHO+ 0.135 CH3OH+ 0.09 CH3CHO+ 1.147 WATER+ 0.018 HCOOH+ 0.045 G-H2+ 0.18 G-CH4+ 1.099 ELE-CHAR
3	CELLAà0.405 CH3COOH+ 1.099 ELE-CHAR+ 1.147 WATER+ 0.419 CO2+ 0.145 H2+ 0.18 GLYOX+ 0.09 CH3CHO+ 0.27 ACETONE+ 0.225 HMFU+ 0.279 CO+ 0.36 HCHO+ 0.135 CH3OH+ 0.018 HCOOH+ 0.045 G-H2+ 0.18 G-CH4
4	GMSWà 0.7 HCEA1+ 0.3 HCEA2+ WATER
5	XYHWà 0.35 HCEA1+ 0.65 HCEA2+ WATER
6	XYGRà0.12 HCEA1+ 0.88 HCEA2+ WATER
7	HCEA1à0.4 CO+ 0.5 CO2+ 0.26 CH4+ 0.64 HCHO+ 0.08 CH3OH+ 0.2 C2H4+ 0.1 C2H5OH+ 0.62 WATER+ 0.02 HCOOH+ 0.22 G-CO2+ 0.32 G-COH2+ 0.1 G-H2+ 0.36 G-CH3OH+ 1.6 ELE-CHAR+ 0.2 H2
8	HCEA1à 0.4 CO+ 1.6 ELE-CHAR+ 0.62 WATER+ 0.5 CO2+ 0.2 H2+ 0.26 CH4+ 0.64 HCHO+ 0.08 CH3OH+ 0.2 C2H4+ 0.1 C2H5OH + 0.02 HCOOH+ 0.22 G-CO2+ 0.32 G-COH2+ 0.1 G-H2+ 0.36 G-CH3OH
9	HCEA1 à 0.08 CO + 0.74 CO2 + 0.24 HCHO + 0.8 WATER + 0.04 HCOOH + 0.12 G-CO2 + 0.12 G-CO + 0.96 G-COH2 + 0.16 G-H2 + 0.5 G-CH4 + 0.3 G-C2H4 + 1.6 ELE-CHAR + 0.2 H2
10	HCEA2 à 0.16 CH3COOH + 0.14 CO + 0.32 CO2 + 0.4 HCHO + 0.08 C2H5OH + 0.76 WATER + 0.02 HCOOH + 0.32 G-CO2 + 0.74 G-COH2 + 0.2 G-CH4 + 0.24 G-CH3OH + 0.22 G-C2H4 + 1.7 ELE-CHAR + 0.2 H2

11	LIGNIN-C à 0.35 LIG-CC + 0.1 PCOUMARY + 0.08 PHENOL + 0.32 CO + 0.3 HCHO + WATER + 0.7 G-COH2 + 0.495 G-CH4 + 0.41 G-C2H4 + 5.735 ELE-CHAR
12	LIGNIN-H à LIG-OH + 0.25 CH3COOH + 0.5 ACETONE + 0.5 G-C2H4
13	LIGNIN-O à LIG-OH + CO2
14	LIG-CC à 0.245 CH3COOH + 0.21 PCOUMARY + 0.14 PHENOL + 0.28 CO + 0.455 CH4 + 0.42 C2H4 + 1.69 WATER + 0.28 G-CO + 0.7 G-COH2 + 9.225 ELE-CHAR + 0.9 H2
15	LIG-OH à LIG + 0.55 CO + 0.05 CO2 + 0.1 CH4 + 0.6 CH3OH + 0.9 WATER + 0.05 HCOOH + 0.6 G-CO + 0.85 G-COH2 + 0.1 G-H2 + 0.35 G-CH4 + 0.3 G-CH3OH + 0.2 G-C2H4 + 4.15 ELE-CHAR
16	LIG à 0.7 FE2MACR + 3.15 ELE-CHAR + 0.9 WATER + 0.15 CO2 + 0.9 H2
17	LIG à 0.14 ACETONE + 0.7 CO + 0.14 CH4 + 0.14 HCHO + 0.28 CH3OH + 0.14 CH3CHO + 1.565 WATER + 0.035 HCOOH + 0.315 G-CO + 0.35 G-COH2 + 0.28 G-CH4 + 0.455 G-C2H4 + 7 ELE-CHAR + 0.15 CO2 + 0.9 H2
18	LIG à0.28 CO + 0.14 CH4 + 0.28 HCHO + 1.32 WATER + 0.14 G-CO + 1.4 G-COH2 + 0.28 G-CH4 + 0.28 G-CH3OH + 0.35 G-C2H4 + 7.35 ELE-CHAR + 0.15 CO2 + 0.9 H2
19	TANN àPHENOL + ITANN + HCOOH
20	ITANN à2 CO + WATER + 5 ELE-CHAR + 0.45 G2-COH2 + 0.55 G-COH2
21	TGLàACROLEIN + 3 FFA
22	G-CO2 àCO2
23	G-CO à0.6 CO + 0.2 ELE-CHAR + 0.2 CO2
24	G-COH2 à 0.75 G2-COH2 + 0.25 H2 + 0.125 CO + 0.0625 CO2 + 0.0625 ELE-CHAR
25	G-H2 à H2
26	G-CH4 à CH4
27	G-CH3OH à 0.6 CH3OH + 0.4 ELE-CHAR + 0.4 WATER + 0.4 H2
28	G-C2H4 à 0.3 C2H4 + 0.7 CH4 + 0.7 ELE-CHAR
29	G2-COH2 à 0.2 G3-COH2 + 0.8 CO + 0.8 H2

30	MOISTURE à WATER
Reactions inside R-103 in Figure S 5	
1	CH3COOH à 1.5 H2 + 1.5 CO + 0.25 CO2 + 0.25 CH4
2	GLYOX à H2 + 2 CO
3	ACETONE à 0.5 CO2 + C2H4 + 0.5 CH4
4	HMFU à3 CO + 1.5 C2H4
5	PCOUMARY à2 CO + 1.5 C2H4 + CH4 + 3 ELE-CHAR
6	PHENOL àCO + C2H4 + 0.5 CH4 + 2.5 ELE-CHAR
7	FE2MACR à4 CO + C2H4 + 2 CH4 + 3 ELE-CHAR
8	HCHOàH2 + CO
9	CH3OH à1.5 H2 + 0.5 CO + 0.25 CO2 + 0.25 CH4
10	CH3CHO àCO + CH4
11	C2H5OH àH2 + CO + CH4
12	HCOOH àH2 + CO2

Table S 20 Past studies on product yields from slow pyrolysis at around 600 ℃ for various lignocellulosic biomass

Ref.	Feedstock	Tempe-rature (℃)	Heat-ing rate	Biochar Yield (w.t.%)	Liquid /Bio-oil Yield (w.t.%)	Gas Yield (w.t.%)	Liquid/ Bio-oil fraction details
Yu et al. (2019)[109]	Hinoki Cypress	600	10-15 °C/min	20.7	63.8	15.5	Heavy oil: 35.5 w.t.%<br>Aqueous: 28.3 w.t.%
Wang et al. (2022)[110]	Corn Stalk		10 °C/min	25.9	55.86	18.2	Reported as total condensates
	Corn Cob			26.56	54.46	19	
	Spruce Wood			25.6	57.43	17	
Gupta et al. (2019)[111]	Teak Sawdust		Not specified	27.4	48.8	23.8	Separation of water is mentioned, but yields are not specified
Altikat et al. (2024)[112]	Thick Stem		Not specified	30.6	12.75	56.64	Low bio-oil is attributed to high temperature cracking into syngas

Benedini et al. (2024)[93]	Wheat Straw Pellets	500	Not specified, total pyrolysis time: 3h	22.9 – 32.1	42.2 – 58.8	17.7 – 25.8	Heavy oil (tar):5.1 – 8.6 w.t.%
							Aqueous: 34.1 – 53.7 w.t.%
Chandrasekaran et al. (2016)[113]	Birch Wood	550	Not specified	19	49	32	Liquid component largely lies in medium boiling points (200-300 °C)
Safavi et al. (2022)[114]	Walnut Shells	600	15 °C/min	23	19	58	Liquid reported as “Tar”; high gas yield may include water vapos or cracking products
Vilas-Boas et al. (2024)[115]	Eucalyptus Sawdust	550	30 °C/min	22 (est.)	59	19 (est.)	Max bio-oil yield condition in this study
	Giant Reed		2 °C/min	33 (est.)	26	41	Low heating rate favored gas/char over oil

Ben & Ragauskas (2013)[116]	Pine Wood	600	3 °C/sec*	23 (est.)	32 (est.)	45 (est.)	Heavy oil: 12 w.t.%<br>Aqueous: 20 w.t. %
	Kraft Lignin	600		47 (est.)	25 (est.)	28 (est.)	Heavy oil: 15 w.t.%<br>Aqueous: 10 w.t.%

*Heating rate 3 °C/sec is higher than conventional slow pyrolysis, but this study refers to the process as "slow" in contrast to their fast pyrolysis condition (2000 °C/sec)

*est.: estimated from texts or figures

Table S 21 Past studies on Hydrocarbon yields and H_2 consumption for various bio-oil

Ref.	Biomass Resource	Bio-oil Elemental Composition (w.t.%)			H_2 usage (g H_2 / g oil feed)	Hydrocarbon yield (%)	Note
		C	H	O			
Khlewee et al. (2019)[92]	Pine	74.6	7.3	18	0.035	79.4	Yield drops as biooil O content increases
		71.5	7.4	21	0.029	66.5	
		65.9	7.1	26.5	0.032	52.4	
Dutta et al. (2015)[8]	Woody Biomass	80	10	10	0.025	84	If consider purging, H_2 usage is 0.063
		83	11	6	0.014	90	If consider purging, H_2 usage is 0.036
Lower et al. (2025)[117]	Crude Glycerol	Not specified		< 3	0.041	93	Hydrotreated biooil comes from condensed vapor from coking (i.e., coke oil)
Jahromi & Agblevor (2017)[118]	Pinyon Juniper	66.9	7.2	25.4	0.096	44.8	
Elliott et al. (2012)[119]	Pine	53	6.4	40.5	0.025 – 0.044	35 – 45	Two-stage hydrotreating required

Table S 22 Graphitization power requirement developed in Gonzalez-Aguirre et al. 2025 [7] that graphitizes 294 t/d coke

Graphitization power requirement breakdown	MW
Coke pretreatment	1.5
Graphitization	26.2
Acid washing	0.4
Graphite post-treatment	18.7

Table S 23 Hydrotreating H_2 and electricity consumption in past literature and BC2

Reference	Feedstock	Hydrotreating method and temperature	Bio-oil Oxygen (wt%)	H_2 usage (kg H_2 / kg oil feed)	Carbon Yield in Oil (%)	Electricity Consumption (hydrotreating unit only)
Elliott et al. (2012)[119]	Pine	Two-stage (170°C/400°C)	40.5	0.025 – 0.044	63	Not reported
Dutta et al (2015) [8]	Woody biomass	Single-stage (375-403°C)	10.5	0.026	91	0.125 kWh/kg oil
Dutta et al (2015) [8]	Woody biomass	Single-stage (375-403°C)	6.4	0.021	94	0.12 kWh / kg oil
Khlewee et al. (2019)[92]	Pine	Single-stage (300°C, 97 bar)	18	0.035	92	Not reported
Khlewee et al. (2019)[92]	Pine	Single-stage (300°C, 97 bar)	26.5	0.032	61	Not reported
Lower et al. (2025)[117]	Crude Glycerol	Single-stage (180 – 385 °C)	< 1	0.041	97	Not reported
Jahromi & Agblevor (2017)[118]	Pinyon Juniper	Single-stage (450 °C, 70 bar)	25.4	0.096	68.8	Not reported
This study (BC2)	Lignocellulosic residues	Two-stage (200°C at 10MPa/400 °C at 15 MPa)	28.6	0.056	88.6	0.478 kWh / kg oil

Table S 24 Reactions in mild and deep HDO

**Mild HDO reactions in R-201 in Figure S 7**		
Fractional conversion	Fractional Conversion of Component	Stoichiometry
1	HCOOH	HCOOH à H2 + CO2
1	HMFU	HMFU + 3 H2 à 2MEFURAN + 2 WATER
1	GLYOX	GLYOX + H2 à HYDACTLD
1	ACETONE	ACETONE + H2 àPROPANOL
1	HCHO	HCHO + H2 àCH3OH
1	CH3CHO	CH3CHO + H2 àC2H5OH
1	ACROLEIN	ACROLEIN + 2 H2 à PROPANOL
1	HYDACTLD	HYDACTLD + H2 àETGLYCL
1	C2H4	C2H4 + H2 àC2H6
0.3	CH3COOH	CH3COOH + 2 H2 àC2H5OH + WATER
**Deep HDO reactions in R-202 in Figure S 7**		
1	CH3COOH	CH3COOH + 3 H2 à C2H6 + 2 WATER
1	PHENOL	PHENOL + H2 à BENZENE + WATER
0.7	BENZENE	BENZENE + 3 H2 à CYHEXANE
1	HYDACTLD	HYDACTLD + H2 à ETGLYCL
1	2MEFURAN	2MEFURAN + 3 H2 àHEXANOL
1	HEXANOL	HEXANOL + H2 à NHEXA + WATER
1	FFA	FFA + 2 H2 à HEPTCANE + CO2
1	PCOUMARY	PCOUMARY + 4 H2 àETHYBENZ + 2 WATER + CH4
1	FE2MACR	FE2MACR + H2 àETHYBENZ + CH4 + 2 CO2
0.5	HCOOH	HCOOH àCO + WATER
0.5	HCOOH	HCOOH àCO2 + H2
1	CH3OH	CH3OH + H2 àCH4 + WATER

1	CH3COOH	CH3COOH + 3 H2 àC2H6 + 2 WATER

References

1. *Report on the Social Cost of Greenhouse Gases: Estimates Incorporating Recent Scientific Advances*. https://www.epa.gov/system/files/documents/2023-12/epa_scghg_2023_report_final.pdf (2023).
2. Nordhaus, W. D. Revisiting the social cost of carbon. *Proc. Natl. Acad. Sci.* **114**, 1518–1523 (2017).
3. Morris, J. *Economic Damage Costs for Nine Human Health and Environmental Impacts*. https://srmginc.com/images/Final-DEQ-Metro-Report.pdf (2020) (accessed 31 October 2025).
4. Newell, R. G., Pizer, W. A. & Prest, B. C. The Shadow Price of Capital: Accounting for Capital Displacement in Benefit-Cost Analysis. *Environ. Energy Policy Econ.* **5**, 49–69 (2024).
5. US EPA. *Guidelines for Preparing Economic Analyses (3rd Edition)*. https://www.epa.gov/environmental-economics/guidelines-preparing-economic-analyses-3rd-edition (2024) (accessed 12 December 2025).
6. Jacobson, J. J., Roni, M. S., Lamers, P. & Cafferty, K. G. *Biomass Feedstock Supply System Design and Analysis*. (2014).
7. Gonzalez-Aguirre, J. A. *et al.* Process design and techno-economic analysis for bio-based graphite and liquid hydrocarbons production from lignocellulosic biomass. *Bioresour. Technol.* **438**, 133156 (2025).
8. Dutta, A. *et al. Process Design and Economics for the Conversion of Lignocellulosic Biomass to Hydrocarbon Fuels: Thermochemical Research Pathways with In Situ and Ex*

Situ Upgrading of Fast Pyrolysis Vapors. https://www.osti.gov/biblio/1215007 (2015) (accessed 20 February 2025) doi:https://doi.org/10.2172/1215007.

9. Debiagi, P. E. A. *et al.* Extractives Extend the Applicability of Multistep Kinetic Scheme of Biomass Pyrolysis. *Energy Fuels* **29**, 6544–6555 (2015).
10. GitHub - wigging/chemics: A Python package for chemical engineering.
11. Lan, K., Zhang, B. & Yao, Y. Circular utilization of urban tree waste contributes to the mitigation of climate change and eutrophication. *One Earth* **5**, 944–957 (2022).
12. Liao, M., Kelley, S. & Yao, Y. Generating Energy and Greenhouse Gas Inventory Data of Activated Carbon Production Using Machine Learning and Kinetic Based Process Simulation. *ACS Sustain. Chem. Eng.* **8**, 1252–1261 (2020).
13. Davis, R. *et al. Process Design and Economics for the Conversion of Lignocellulosic Biomass to Hydrocarbons: Dilute-Acid and Enzymatic Deconstruction of Biomass to Sugars and Catalytic Conversion of Sugars to Hydrocarbons*. NREL/TP--5100-62498, 1176746 http://www.osti.gov/servlets/purl/1176746/ (2015) (accessed 16 April 2024) doi:10.2172/1176746.
14. Dutta, A. *et al. Ex Situ Catalytic Fast Pyrolysis of Lignocellulosic Biomass to Hydrocarbon Fuels: 2020 State of Technology*. NREL/TP-5100-80291, 1805204, MainId:42494 https://www.osti.gov/servlets/purl/1805204/ (2021) (accessed 14 June 2025) doi:10.2172/1805204.
15. Brautaset, K.-M. Techno-economic Analysis of Sawdust Derived Hard Carbon for LIBs. (NTNU, 2024).
16. Kieush, L. *et al.* Comprehensive microstructural evaluation of biochar, biocoke, and anthracite with respect to the CO2 reactivity. *Results Eng.* **27**, 105690 (2025).

17. Ranzi, E., Debiagi, P. E. A. & Frassoldati, A. Mathematical Modeling of Fast Biomass Pyrolysis and Bio-Oil Formation. Note II: Secondary Gas-Phase Reactions and Bio-Oil Formation. *ACS Sustain. Chem. Eng.* **5**, 2882–2896 (2017).

18. Ranzi, E., Debiagi, P. E. A. & Frassoldati, A. Mathematical Modeling of Fast Biomass Pyrolysis and Bio-Oil Formation. Note I: Kinetic Mechanism of Biomass Pyrolysis. *ACS Sustain. Chem. Eng.* **5**, 2867–2881 (2017).

19. Meier, H. F. *et al.* A kinetic model for thermal cracking of waste cooking oil based on chemical lumps. *Fuel* **144**, 50–59 (2015).

20. Shi, Z. *et al.* Bio-based anode material production for lithium–ion batteries through catalytic graphitization of biochar: the deployment of hybrid catalysts. *Sci. Rep.* **14**, 3966 (2024).

21. Shi, Z. *et al.* Establishment of green graphite industry: Graphite from biomass and its various applications. *SusMat* **3**, 402–415 (2023).

22. Gao, Y. *et al.* Acid recovering and iron recycling from pickling waste acid by extraction and spray pyrolysis techniques. *J. Clean. Prod.* **312**, 127747 (2021).

23. Dey, S. C. *et al.* Catalytic graphitization of pyrolysis oil for anode application in lithium-ion batteries. *Green Chem.* **26**, 8840–8853 (2024).

24. Gulas, M. *et al.* High-Purity Biomass-Derived Synthetic Graphite: Catalyst-Free Industrial Synthesis and Applications. *ACS Omega* **11**, 9435–9450 (2026).

25. Banek, N. A., McKenzie, K. R., Abele, D. T. & Wagner, M. J. Sustainable conversion of biomass to rationally designed lithium-ion battery graphite. *Sci. Rep.* **12**, 8080 (2022).

26. Vook, T. *et al.* Sustainable Li-ion anode material from Fe-catalyzed graphitization of paper waste. *J. Energy Storage* **73**, 109242 (2023).

27. You, H. *et al.* Sustainable Production of Biomass-Derived Graphite and Graphene Conductive Inks from Biochar. *Small* **20**, 2406669 (2024).

28. Gil, A., Sancho-Sanz, I. & Korili, S. A. Progress and Perspectives in the Catalytic Hydrotreatment of Bio-Oils: Effect of the Nature of the Metal Catalyst. *Ind. Eng. Chem. Res.* **63**, 11759–11775 (2024).

29. Han, Y. *et al.* Hydrotreatment of pyrolysis bio-oil: A review. *Fuel Process. Technol.* **195**, 106140 (2019).

30. Dutta, A. Ex Situ and In Situ Catalytic Fast Pyrolysis Models (ASPEN PLUS MODELS) [SWR-16-12]. National Renewable Energy Laboratory (NREL), Golden, CO (United States) https://doi.org/10.11578/DC.20190515.4 (2019).

31. Jones, S. *et al. Production of Gasoline and Diesel from Biomass via Fast Pyrolysis, Hydrotreating and Hydrocracking: A Design Case*. (2009).

32. Dehimi, L. *et al.* Hydrogen production by the water-gas shift reaction: A comprehensive review on catalysts, kinetics, and reaction mechanism. *Fuel Process. Technol.* **267**, 108163 (2025).

33. Phan, T. S., Pham Minh, D., Espitalier, F., Nzihou, A. & Grouset, D. Hydrogen production from biogas: Process optimization using ASPEN Plus®. *Int. J. Hydrog. Energy* **47**, 42027–42039 (2022).

34. Großmann, K., Treiber, P. & Karl, J. Steam methane reforming at low S/C ratios for power-to-gas applications. *Int. J. Hydrog. Energy* **41**, 17784–17792 (2016).

35. Snowden-Swan, L., Schmidt, A., Fuller, C. & Anderson, D. *Hydrothermal Processing of Wastewater Solids (HYPOWERs) Project Preliminary Techno- Economic Analysis*.

https://www.pnnl.gov/main/publications/external/technical_reports/PNNL-36528.pdf (2024).

36. SundarRajan, P. *et al.* Insights into valuing the aqueous phase derived from hydrothermal liquefaction. *Renew. Sustain. Energy Rev.* **144**, 111019 (2021).

37. Boruah, B. *et al. Catalytic Conversion of Biogenic and Synthetic Polymers into Carbon-Negative and -Neutral Chemicals and Fuels*. (2024).

38. Solutions, L. D. X. Oxidizers RTO | Regenerative Thermal Oxidizers. *LDX Solutions* https://www.ldxsolutions.com/solutions/vocs/oxidizers-rto/ (accessed 2 March 2026).

39. NISTIR 5078. https://www.nist.gov/system/files/documents/srd/NISTIR5078.htm (accessed 7 October 2025).

40. Water Properties: Vaporization Heat vs. Temperature. *The Engineering ToolBox* https://www.engineeringtoolbox.com/water-properties-d_1573.html (2018) (accessed 29 August 2026).

41. Cutler, D. S., Dean, J. D., Daw, J. A. & Howett, D. *Alternative Water Treatment Technologies for Cooling Tower Applications*. NREL/TP--7A40-71845, 1496058 http://www.osti.gov/servlets/purl/1496058/ (2019) (accessed 7 October 2025) doi:10.2172/1496058.

42. Dutta, A. *et al. Process Design and Economics for Conversion of Lignocellulosic Biomass to Ethanol: Thermochemical Pathway by Indirect Gasification and Mixed Alcohol Synthesis*. NREL/TP-5100-51400, 1015885 http://www.osti.gov/servlets/purl/1015885/ (2011) (accessed 2 March 2026) doi:10.2172/1015885.

43. EnergyPlus. https://energyplus.net/ (accessed 3 March 2026).

44. Pumping Plant Performance | NC State Extension Publications. https://content.ces.ncsu.edu/pumping-plant-performance (accessed 3 March 2026).

45. Gilbert A. McCoy & Douglass, J. G. *Premium Efficiency Motor Selection And Application Guide: A Guidebook for Industry*. https://www.energy.gov/sites/prod/files/2014/04/f15/amo_motors_handbook_web.pdf? (2014) (accessed 3 March 2026).

46. Fu, Z., Marston, A. & Baumann, O. *Cooling Tower, Pump and Fan Component Constraints of Ener- gyPlus™ When Performing the Whole Building Energy Simulation for LEED*. (2014).

47. Goel, S., Rosenberg, M., Mets, E. & Eley, C. *Standard 90.1-2019 Performance Rating Method Reference Manual*. (2023).

48. Ecoinvent geography definitions — Ecoinvent-topology 3.10 documentation. https://geography.ecoinvent.org/ (accessed 31 October 2025).

49. NETL-RIC/ElectricityLCI. National Energy Technology Laboratory Research & Innovation Center (2025).

50. Glossary - U.S. Energy Information Administration (EIA). https://www.eia.gov/tools/glossary/index.php (accessed 31 October 2025).

51. brentq — SciPy v1.18.0 Manual. https://docs.scipy.org/doc/scipy/reference/generated/scipy.optimize.brentq.html (accessed 29 August 2026).

52. U.S. Geological Survey. *2022 Minerals Yearbook - Graphite (Advanced Release)*. https://pubs.usgs.gov/myb/vol1/2022/myb1-2022-graphite.pdf (2025) (accessed 21 February 2026).

53. Davis, M. *et al.* Chapter 4: Biomass from the Forested Land Base. Oak Ridge National Laboratory (ORNL), Oak Ridge, TN (United States), Bioenergy Knowledge Discovery Framework (BEKDF) https://doi.org/10.23720/BT2023/2316170 (2024).

54. Kulkarni, S. *et al.* Prospective Life Cycle Assessment of Synthetic Graphite Manufactured via Electrochemical Graphitization. *ACS Sustain. Chem. Eng.* **10**, 13607–13618 (2022).

55. Engels, P. *et al.* Life cycle assessment of natural graphite production for lithium-ion battery anodes based on industrial primary data. *J. Clean. Prod.* **336**, 130474 (2022).

56. ecoinvent Version 3.9.1. https://support.ecoinvent.org/ecoinvent-version-3.9.1 (accessed 31 October 2025).

57. U.S. Energy Information Administration (EIA). Spot Prices for Crude Oil and Petroleum Products. https://www.eia.gov/dnav/pet/pet_pri_spt_s1_m.htm (accessed 3 March 2026).

58. Biodiesel – Market Intelligence. *ProcurementIQ* https://www.procurementiq.com/market-intelligence/biodiesel (accessed 3 March 2026).

59. Daily Jet Fuel Price Comparison: Argus Sustainable vs. Conventional Jet Fuel. *Airlines For America* https://www.airlines.org/dataset/saf-vs-jet-fuel-comparison/ (accessed 3 March 2026).

60. The Hydrogen Transition is Here – A Simplified Glance at Major Factors and Concerns. *CSEG RECORDER Magazine* https://www.csegrecorder.com/articles/view/the-hydrogen-transition-is-here-a-simplified-glance-at-major-factors (accessed 3 March 2026).

61. Curcio, E. Techno-economic analysis of hydrogen production: Costs, policies, and scalability in the transition to net-zero. *Int. J. Hydrog. Energy* **128**, 473–487 (2025).

62. Electric Power Monthly - U.S. Energy Information Administration (EIA). https://www.eia.gov/electricity/monthly/epm_table_grapher.php?t=epmt_5_6_a (accessed 7 November 2025).

63. Average Price of Electricity to Ultimate Customers by End-Use Sector - U.S. Energy Information Administration (EIA). https://www.eia.gov/electricity/monthly/epm_table_grapher.php (accessed 27 August 2026).

64. Mike. Diesel price index. *businessanalytiq* https://businessanalytiq.com/procurementanalytics/index/diesel-price-index/ (2020) (accessed 3 March 2026).

65. The Price Of Liquid Nitrogen In The United States. *Rutherford & Titan* https://www.rutherfordtitan.com/liquid-nitrogen-generators/liquid-nitrogen-price-usa/ (accessed 3 March 2026).

66. United States Natural Gas Industrial Price (Dollars per Thousand Cubic Feet). https://www.eia.gov/dnav/ng/hist/n3035us3m.htm (accessed 3 March 2026).

67. Billion-Ton 2023 Data Portal | BioEnergy KDF. https://bioenergykdf.ornl.gov/bt23-data-portal (accessed 22 November 2025).

68. Wang, Z., Dunn, J. B., Han, J. & Wang, M. Q. *Material and Energy Flows in the Production of Cellulosic Feedstocks for Biofuels for the GREET Model*. https://www.osti.gov/biblio/1148669 (2013) (accessed 3 March 2026) doi:10.2172/1148669.

69. Iyer, R. K. & Kelly, J. C. *Updated Production Inventory for Lithium-Ion Battery Anodes for the GREET® Model, and Review of Advanced Battery Chemistries*.

https://www.osti.gov/biblio/1891640 (2022) (accessed 12 December 2025) doi:10.2172/1891640.

70. Sahoo, K., Bilek, E., Bergman, R. & Mani, S. Techno-economic analysis of producing solid biofuels and biochar from forest residues using portable systems. *Appl. Energy* **235**, 578–590 (2019).

71. Puettmann, M., Sahoo, K., Wilson, K. & Oneil, E. Life cycle assessment of biochar produced from forest residues using portable systems. *J. Clean. Prod.* **250**, 119564 (2020).

72. Banek, N. A., Abele, D. T., McKenzie, K. R. Jr. & Wagner, M. J. Sustainable Conversion of Lignocellulose to High-Purity, Highly Crystalline Flake Potato Graphite. *ACS Sustain. Chem. Eng.* **6**, 13199–13207 (2018).

73. Shi, Z. *et al.* Establishment of green graphite industry: Graphite from biomass and its various applications. *SusMat* **3**, 402–415 (2023).

74. Shi, Z. *et al.* Catalytic graphitization of engineered pyrolysis bio-oil for sustainable graphite and hydrogen Co-production. *Renew. Energy* **256**, 124149 (2026).

75. Dey, S. C. *et al.* Low-Temperature Processing of Pyrolysis Bio-Oil for Sustainable Biographite Production. *Energy Fuels* **39**, 11372–11387 (2025).

76. Frankenstein, L. *et al.* Revealing the Impact of Different Iron-Based Precursors on the 'Catalytic' Graphitization for Synthesis of Anode Materials for Lithium Ion Batteries. *ChemElectroChem* **10**, e202201073 (2023).

77. Gomez-Martin, A., Schnepp, Z. & Ramirez-Rico, J. Structural Evolution in Iron-Catalyzed Graphitization of Hard Carbons. *Chem. Mater.* **33**, 3087–3097 (2021).

78. Hanhart, V. *et al.* Insights into the Impact of Activators on the 'Catalytic' Graphitization to Design Anode Materials for Lithium Ion Batteries. *ChemElectroChem* **9**, e202200819 (2022).

79. U.S. Energy Information Administration (EIA) Independent Statistics and Analysis - Wood and biomass waste price and expenditure estimates. https://www.eia.gov/state/seds/data.php?incfile=/state/seds/sep_fuel/html/fuel_pr_ww.html&sid=US (accessed 31 October 2025).

80. *Default Heat Content for Energy Conversions*. https://www.epa.gov/system/files/documents/2022-10/Default%20Heat%20Content%20Ratios%20for%20Help%20and%20User%20Guide%20%281%29.pdf? (accessed 13 August 2026).

81. US EPA. Tool for Reduction and Assessment of Chemicals and Other Environmental Impacts (TRACI). https://www.epa.gov/chemical-research/tool-reduction-and-assessment-chemicals-and-other-environmental-impacts-traci (2015) (accessed 31 October 2025).

82. When is bulk best? https://www.linde-gas.com/products-and-services/supply-modes/bulk-supply (accessed 23 August 2026).

83. Where can I buy nitrogen? https://www.linde-gas.com/products-and-services/gases/nitrogen (accessed 23 August 2026).

84. Liquid Nitrogen Vaporizer Working Principle Explained. https://www.gaseschina.com/liquid-nitrogen-vaporizer-working-principle/ (2026) (accessed 23 August 2026).

85. Bioenergy Feedstock Library. https://bioenergylibrary.inl.gov/Sample/BiomassInfo.aspx (accessed 21 November 2025).

86. Negro, M. J. *et al.* Sugars Production from Municipal Forestry and Greening Wastes Pretreated by an Integrated Steam Explosion-Based Process. *Energies* **13**, (2020).

87. Sagues, W. J. *et al.* A simple method for producing bio-based anode materials for lithium-ion batteries. *Green Chem.* **22**, 7093–7108 (2020).

88. Béguerie, T., Weiss-Hortala, E., Lyczko, N. & Nzihou, A. The mechanisms of calcium-catalyzed graphenization of cellulose and lignin biochars uncovered. *Sci. Rep.* **13**, 11390 (2023).

89. Lee, K., Ryu, S.-K., Kim, H.-G., Kwac, L.-K. & Kim, Y.-S. Comparative Analysis of Graphitization Characteristics in Bamboo and Oak Charcoals for Secondary Battery Anodes. *Crystals* **14**, 914 (2024).

90. Shi, Z. Iron-catalyzed graphitization of biochar to produce graphitic carbon materials. (2021).

91. Wang, H. *et al.* Performance and techno-economic evaluations of co-processing residual heavy fraction in bio-oil hydrotreating. *Catal. Today* **365**, 357–364 (2021).

92. Khlewee, M., Gunukula, S., Wheeler, M. C. & DeSisto, W. J. Hydrotreating of reduced oxygen content bio-oils produced by formate-assisted pyrolysis. *Fuel* **254**, 115570 (2019).

93. Benedini, L., Dalsgaard Jensen, C., Ahrenfeldt, J. & Birk Henriksen, U. Production and characterization of two-step condensation bio-oil from pyrolysis. *J. Anal. Appl. Pyrolysis* **183**, 106744 (2024).

94. Jones, S. *et al. Process Design and Economics for the Conversion of Lignocellulosic Biomass to Hydrocarbon Fuels: Fast Pyrolysis and Hydrotreating Bio-Oil Pathway*. https://www.osti.gov/biblio/1115839 (2013) doi:https://doi.org/10.2172/1115839.

95. Bangalore Ashok, R. P., Oinas, P. & Forssell, S. Techno-economic evaluation of a biorefinery to produce γ-valerolactone (GVL), 2-methyltetrahydrofuran (2-MTHF) and 5-hydroxymethylfurfural (5-HMF) from spruce. *Renew. Energy* **190**, 396–407 (2022).

96. Puschnigg, S., Fazeni-Fraisl, K., Lindorfer, J. & Kienberger, T. Biorefinery development for the conversion of softwood residues into sustainable aviation fuel: Implications from life cycle assessment and energetic-exergetic analyses. *J. Clean. Prod.* **386**, 135815 (2023).

97. Luanwuthi, S. *et al.* Techno-economic and Environmental Assessment of Activated Carbon Electrodes for Supercapacitors from Oil Palm Leaves. *ACS Sustain. Chem. Eng.* **12**, 14446–14458 (2024).

98. Hawkins, T. *et al. The Role of Biofuels and Biomass Feedstocks for Decarbonizing the U.S. Economy by 2050 - (DECARB) Decarbonizing Energy Through Collaborative Analysis of Routes and Benefits*. NREL/TP--5100-87279, 2337775, MainId:88054 https://www.osti.gov/servlets/purl/2337775/ (2024) (accessed 21 August 2025) doi:10.2172/2337775.

99. Average Price of Electricity to Ultimate customers - U.S. Energy Information Administration (EIA). https://www.eia.gov/electricity/monthly/epm_table_grapher.php (accessed 28 August 2026).

100. Second Stage Steam Autothermal Reforming Catalyst For Methane Hydrogen Conversion Al2o3-nio Material 99% Purity Ammonia Systems - Buy Second Stage Steam Reforming Conversion From Methane To Hydrogen Autothermal Reforming Catalyst 17*17*9mm hydrogen Autothermal Reforming Catalyst second Stage Steam Reforming methane To Hydrogen Conversion Catalyst custom Made Reforming Conversion Catalyst al2o3-nio

Reforming Catalyst Product on Alibaba.com. https://www.alibaba.com/product-detail/Second-Stage-Steam-Reforming-Conversion-From_1601011392185.html?spm=a2700.7724857.0.0.e8bd3ddeHiTgok (accessed 3 March 2026).

101. US EPA, O. Data and Information Used by WaterSense. https://www.epa.gov/watersense/data-and-information-used-watersense (2020) (accessed 3 March 2026).

102. Sodium Hydroxide (LYE). *Bulk Apothecary* https://www.bulkapothecary.com/sodium-hydroxide-lye/ (accessed 3 March 2026).

103. Klein, B., McNamara, I., Davis, R., Mittal, A. & Johnson, D. *Techno-Economic Assessment for the Production of Hydrocarbon Fuels via Catalytic Upgrading of Furans*. NREL/TP-5100-80652, 1819430, MainId:66383 https://www.osti.gov/servlets/purl/1819430/ (2021) (accessed 23 August 2026) doi:10.2172/1819430.

104. Mike. Argon price index. *businessanalytiq* https://businessanalytiq.com/procurementanalytics/index/argon-price-index/ (2023) (accessed 3 March 2026).

105. Nickel Based Alumina Ni Al2o3 Catalyst Catalyst Used For Methanation Catalyst For Co Conversion Or Removal - Buy Nickel Ni Based Alumina Al2o3 Catalyst Ni Based Alumina Catalyst methanation Catalyst Gamma Alumina Catalyst Nickel Oxide Catalyst Alumina Catalyst Pellet nimo/al2o3 ni/al2o3 como/al2o3catalyst Product on Alibaba.com. https://www.alibaba.com/product-detail/Nickel-Based-Alumina-Ni-Al2o3-

Catalyst_1601233861264.html?spm=a2700.prosearch.normal_offer.d_title.743367afBkB0ZS&priceId=06b055ab307845269e56d4ca8ea9fa9b (accessed 3 March 2026).

106. Bhuwalka, K. *et al.* Securing the supply of graphite for batteries. Preprint at https://doi.org/10.48550/arXiv.2503.21521 (2025).

107. Lower, L. *et al.* Catalytic Graphitization of Biocarbon for Lithium-Ion Anodes: A Minireview. *ChemSusChem* **16**, e202300729 (2023).

108. Park, J. *et al.* Overview of graphite supply chain and its challenges. *Geosci. J.* **29**, 329–341 (2025).

109. Yu, S., Park, J., Kim, M., Ryu, C. & Park, J. Characterization of biochar and byproducts from slow pyrolysis of hinoki cypress. *Bioresour. Technol. Rep.* **6**, 217–222 (2019).

110. Wang, L. *et al.* Comparison of properties of biochar produced from different types of lignocellulosic biomass by slow pyrolysis at 600 °C. *Appl. Energy Combust. Sci.* **12**, 100090 (2022).

111. Gupta, G. K., Gupta, P. K. & Mondal, M. K. Experimental process parameters optimization and in-depth product characterizations for teak sawdust pyrolysis. *Waste Manag.* **87**, 499–511 (2019).

112. Altıkat, A., Alma, M. H., Altıkat, A., Bilgili, M. E. & Altıkat, S. A Comprehensive Study of Biochar Yield and Quality Concerning Pyrolysis Conditions: A Multifaceted Approach. *Sustainability* **16**, 937 (2024).

113. Chandrasekaran, S. R. *et al.* Antioxidants from Slow Pyrolysis Bio-Oil of Birch Wood: Application for Biodiesel and Biobased Lubricants. *ACS Sustain. Chem. Eng.* **4**, 1414–1421 (2016).

114. Safavi, A., Richter, C. & Unnthorsson, R. Mathematical Modeling and Experiments on Pyrolysis of Walnut Shells Using a Fixed-Bed Reactor. *ChemEngineering* **6**, 93 (2022).

115. M. Vilas-Boas, A. C. *et al.* Valorisation of residual biomass by pyrolysis: influence of process conditions on products. *Sustain. Energy Fuels* **8**, 379–396 (2024).

116. Ben, H. & Ragauskas, A. J. Comparison for the compositions of fast and slow pyrolysis oils by NMR characterization. *Bioresour. Technol.* **147**, 577–584 (2013).

117. Lower, L. *et al.* Sustainable Graphite and Jet Fuel from Biorefinery Residue. *ChemSusChem* **18**, e202402509 (2025).

118. Jahromi, H. & Agblevor, F. A. Upgrading of pinyon-juniper catalytic pyrolysis oil via hydrodeoxygenation. *Energy* **141**, 2186–2195 (2017).

119. Elliott, D. C. *et al.* Catalytic Hydroprocessing of Fast Pyrolysis Bio-oil from Pine Sawdust. *Energy Fuels* **26**, 3891–3896 (2012).
